# Recent advances in Phlebotomine sand fly research: a review based on studies presented at ISOPS XI

**DOI:** 10.1051/parasite/2025062

**Published:** 2025-10-29

**Authors:** Vladimir Ivović, Gioia Bongiorno, Petr Volf, Yara Traub Cseko, Jeffrey Shaw, Dia Elnaiem, Shaden Kamhawi, Eva Iniguez, Carla Maia, Suzana Blesić, Padet Siriyasatien, Vit Dvorak, Yusuf Ozbel, Jérôme Depaquit

**Affiliations:** 1 Faculty of Mathematics, Natural Sciences and Information Technologies, University of Primorska 6000 Koper Slovenia; 2 Department of Infectious Diseases, Vector-Borne Diseases Unit, Istituto Superiore di Sanità 00161 Rome Italy; 3 Department of Parasitology, Faculty of Science, Charles University 128 00 Prague 2 Czech Republic; 4 Laboratório de Biologia Molecular de Parasitos e Vetores, Instituto Oswaldo Cruz, Fiocruz 21040-360 Rio de Janeiro RJ Brazil; 5 Department of Parasitology, Institute of Biomedical Sciences, University of São Paulo 05508-000 São Paulo Brazil; 6 Department of Natural Sciences, University of Maryland Eastern Shore Princess Anne MD 21853 USA; 7 Vector Molecular Biology Section, Laboratory of Malaria and Vector Research, National Institute of Allergy and Infectious Diseases, National Institutes of Health Rockville MD 20852 USA; 8 Global Health and Tropical Medicine (GHTM), Associate Laboratory in Translation and Innovation Towards Global Health (LA-REAL), Instituto de Higiene e Medicina Tropical (IHMT), Universidade Nova de Lisboa (UNL) 1349-008 Lisboa Portugal; 9 Institute for Medical Research, University of Belgrade 11000 Belgrade Serbia; 10 Vector Biology and Vector Borne Disease Research Unit, Department of Parasitology, Faculty of Medicine, Chulalongkorn University 10330 Bangkok Thailand; 11 Department of Parasitology, Faculty of Medicine, Ege University 35100 Bornova Izmir Türkiye; 12 Faculté de Pharmacie, Université de Reims Champagne Ardenne, UR ESCAPE-USC ANSES PETARD 51097 Reims Cedex France

**Keywords:** Phlebotomine sand flies, Vector competence, Surveillance, Climate change, Taxonomy, ISOPS

## Abstract

The 11th International Symposium on Phlebotomine Sand flies (ISOPS XI) took place in Portorož, Slovenia, in September 2024 and brought together experts from around the world to discuss recent advances in the biology, ecology and control of phlebotomine sand flies and the pathogens they transmit. This report summarises the key findings of the symposium and is organised thematically by session. Key topics included the development of refined experimental models of *Leishmania* transmission, new insights into the interactions between vector, parasite and microbiota, and the detection of *Leishmania donovani* in new geographic regions. Advances in molecular diagnostics and surveillance technologies were emphasised, as were emerging concerns about insecticide resistance. The potential of paratransgenesis and symbiont-based vector control approaches was also emphasised. In a separate session, the CLIMOS project was presented, which integrates climate monitoring, ecological modelling and public health tools to develop an early warning system (EWS) for sand fly-borne diseases. Overall, the contributions to the symposium reflect the dynamic development of sand fly research in response to global environmental change and emphasise the importance of international collaboration in combating emerging vector-borne diseases.

## Abstract (fr)

Le 11e Symposium international sur les phlébotomes (ISOPS XI) s’est tenu à Portorož, en Slovénie, en septembre 2024. Il a réuni des experts du monde entier pour discuter des avancées récentes en biologie, en écologie et en lutte contre les phlébotomes et les agents pathogènes qu’ils transmettent. Cette revue résume les principaux résultats présentés lors du symposium, organisés par session thématique. Les principaux sujets abordés comprenaient le développement de modèles expérimentaux affinés de transmission de *Leishmania*, de nouvelles connaissances sur les interactions vecteur-parasite-microbiote et la détection de *Leishmania donovani* dans de nouvelles régions géographiques. Les avancées en matière de diagnostic moléculaire et de technologies de surveillance ont été mises en avant, ainsi que les préoccupations émergentes concernant la résistance aux insecticides. Le potentiel de la paratransgénèse et des approches de lutte antivectorielle basées sur les symbiotes a également été souligné. Une session dédiée a présenté le projet CLIMOS, qui intègre la surveillance du climat, la modélisation écologique et des outils de santé publique pour développer un système d’alerte précoce (SAP) pour les maladies transmises par les phlébotomes. Collectivement, les contributions du symposium reflètent l’évolution dynamique de la recherche sur les phlébotomes face aux changements environnementaux mondiaux et soulignent l’importance de la collaboration internationale pour faire face aux menaces émergentes de maladies vectorielles.

## Introduction

Phlebotomine sand flies (Diptera: Psychodidae, Phlebotominae) continue to be the subject of intense scientific interest due to their role as vectors of various pathogens of public health and veterinary importance, in particular *Leishmania* spp. and various phleboviruses. These small insects are responsible for the transmission of leishmaniasis, a group of neglected tropical diseases with complex zoonotic and anthroponotic transmission cycles whose dynamics are increasingly influenced by environmental and socio-economic changes.

Recognising the growing need for international collaboration and knowledge exchange in this field, the International Symposium on Phlebotomine Sand flies (ISOPS) has established itself as an important scientific forum since its inception in 1991. The ISOPS takes place approximately every four years and brings together researchers from the fields of entomology, parasitology, molecular biology, public health and related disciplines. Each symposium has proven to be a landmark event summarising recent advances in sand fly biology, vector-pathogen interactions, epidemiology and control strategies.

The 11th ISOPS Symposium, which took place in Portorož, Slovenia, from 9 to 13 September 2024, continued this tradition and gathered over 130 participants from all over the world. The Symposium, organised by the University of Primorska (Koper, Slovenia), covered a wide range of topics including sand fly systematics, vector competence, host-parasite interactions, pathogen detection and climate-induced changes in disease distribution. Of particular note, the symposium also included a session on the CLIMOS project, a European initiative focussing on the development of an early warning system (EWS) for sand fly-borne diseases.

This report summarises the key findings of ISOPS XI and is divided into the main thematic sessions of the meeting. It aims to provide an overview of current research trends, highlight methodological innovations and identify gaps and priorities for future sand fly and pathogen research. Numbers between parentheses refer to the communication in the Abstract Book (Supplementary file).

## Experimental models and vector interactions in *Leishmania* transmission

New findings on the mechanisms that determine vector competence were presented in several talks. Tiago Serafim (O44) showed that host IgM promotes the genetic exchange of *Leishmania* in sand flies, while Rodrigo Soares (O45) demonstrated the species-specific plasticity of salivary glands in *Nyssomyia neivai*. Cecilia Stahl Vieira (O46) and Erich Telleria (O47) showed how plant compounds and microbial components influence the immunity and gut physiology of sand flies.

Yara Traub Cseko (O48) compared Amazonian sand fly populations from endemic and non-endemic areas using proteomic analyses of the gut and found differences in the abundance of proteins related to immunity and gut function [43]. Metagenomics of the guts of the two vector populations revealed differences in the composition of the microbiota that could also help to explain differences in parasite transmission capacity [28]. Finally, Barbora Vojtková (O49) investigated the infectiousness of natural *Leishmania major* hosts and provided important insights into their role in parasite transmission.

Understanding the transmission dynamics of *Leishmania* parasites requires experimental models that faithfully reproduce natural infection. Several ISOPS XI presentations focused on optimising such models, particularly those based on transmission through sand fly bites, which more realistically reflect the complex parasite-vector-host interactions than needle inoculations.

Márcia Lauren (O1) developed *in vitro* and *in vivo* models of atypical cutaneous leishmaniasis (non-ulcerative cutaneous leishmaniasis) caused by *L. infantum* using salivary gland homogenates (SGH) of *Lutzomyia longipalpis*. While SGH increased infection rates in macrophages *in vitro*, it did not lead to pathology or detectable parasite loads in BALB/c mice, suggesting that additional factors are required for the establishment of infection *in vivo*. Similarly, Kristýna Jelínková (O2) investigated how the timing and frequency of exposure to *Phlebotomus duboscqi* saliva affects the immune response and disease progression in BALB/c mice during *L. major* infection. They showed that repeated exposure to saliva does not reduce the protective effect, but instead increases the infiltration of myeloid cells and the development of lesions.

An important advance was made by Tomáš Bečvář (O3), who showed that biting midges (*Culicoides sonorensis*) can promote the development and transmission of *Leishmania* (*Mundinia*) parasites, including *L.* (*Mundinia*) *martiniquensis* and *L.* (*Mundinia*) *orientalis*. In contrast, several sand fly species tested were refractory. In the subsequent discussion, it was emphasised that the vector competence of the Southeast Asian species needs to be investigated. These results suggest that biting midges may act as important *Mundinia* vectors, raising the possibility that sand flies are not the exclusive vectors of *Leishmania*, which requires further field confirmation.

Sarah Hendrickx and Guy Caljon (O4) described the optimisation of transmission models for drug and vaccine testing, with the aim of better mimicking natural infection in several *Leishmania* species (*L. major*, *L. tropica* and *L. braziliensis*). Using *Lu. longipalpis* in combination with bioluminescent and fluorescent parasites, they developed systems that allow dynamic tracking of infection progression and severity in rodents, depending on parasite strain, time of exposure and bite site. A robust transmission model for *L. major* has already been established, while work for *L. tropica* and *L. braziliensis* is still ongoing.

Finally, Petr Volf (O5) pointed out two mechanisms of attachment of *Leishmania* to the midgut and the stomodeal valve of the sand fly, each mediated by specific molecules. In *Ph. papatasi*, binding to the midgut depends on the recognition of a sugar unit on the lipophosphoglycan (LPG) of *L. major* by galectins. In permissive sand flies, attachment occurs *via* O-linked glycoproteins on the midgut epithelium. Haptomonads adhere strongly to the lining of the stomodeal valves, damaging them and facilitating parasite transmission by regurgitation. The authors identified kinetoplastid insect adhesion proteins (KIAPs) essential for attachment of haptomonads in *L. mexicana*; deletion of KIAPs resulted in disruption of transmission without affecting midgut colonisation [46]. These results highlight the crucial role of parasite molecules in the colonisation of the vector midgut and transmission to the vertebrate host.

Taken together, these studies illustrate the ongoing refinement of experimental systems that not only improve the understanding of vector competence and host-pathogen interactions, but also provide indispensable platforms for testing vaccines and strategies to prevent transmission.

## Advances in *Leishmania* research

New findings on the interactions between parasites, vectors and hosts were an important topic at ISOPS XI. Recently, there has been increasing evidence for the importance of microbiota in vector-parasite interactions. In the case of sand flies and *Leishmania* spp., it has already been shown that some bacteria act against *Leishmania* or affect the interaction between parasite and vector in some way. For example, *Serratia* has lytic activity against various *Leishmania* species [18, 30, 31, 39]. An opposite effect was observed when treatment of sand flies with antibiotics resulted in flies that were refractory to infection, highlighting the importance of microbiota for the successful establishment of *Leishmania* infection in the vector [27]. Pedro Cecilio (O6) reported that colonisation of *Ph. duboscqi* with *Delftia tsuruhatensis* TC1, a bacterium previously shown to inhibit the development of *Plasmodium* in mosquitoes [19], significantly reduced the development and transmissibility of *L. major*. This phenotype is probably an indirect consequence of colonisation with *D. tsuruhatensis*, which is related to the induction of dysbiosis in the gut of the sand fly, in which a *Serratia* bacterium grows excessively. This is consistent with the fact that the hydrophobic molecule harmane, which is responsible for the refractoriness of mosquitoes to the *Plasmodium* parasite, does not appear to be responsible for killing *Leishmania* in the vector, which has already been shown *in vitro* by Di Giorgio *et al.* [14]. Importantly, *Leishmania*-infected sand flies fed with *D. tsuruhatensis* are less able to transmit *L. major* parasites and cause disease in mice. This work was recently published [12]. Modelling supports the disruption of disease endemicity in the field when the vector is infected with this microorganism, highlighting *D. tsuruhatensis* as a promising agent for the control of leishmaniasis.

Kinetoplast DNA (kDNA) has long been recognised as an extremely sensitive and specific diagnostic target for the detection of *Leishmania* parasites and can be used, for example, for the detection of latent infections [1]. It was presented as a diagnostic innovation by Aïda Bouratbine (O7), who demonstrated the value of kinetoplast DNA-targeted qPCR for the detection of low-level *Leishmania infantum* infections in sand flies. This technique proved to be particularly useful in regions with low transmission and helped to distinguish vectors when classical methods failed. The group had already demonstrated the importance of using kDNA in areas of low transmission in Tunisia [48]. In the present studies, *Leishmania* kDNA screening was used for infection and identification of *L. infantum* vectors in hypoendemic foci of human leishmaniasis, and qPCR with mini-circle kDNA was shown to be a highly sensitive method for *Leishmania* identification, capable of detecting one flagellate per dissected sand fly gut, legitimising its use as a screening method for the investigation of sand fly infections. In addition, kDNA qPCR can be used to determine parasite load, with a high load correlating with clear evidence of *Leishmania* transmission.

As recently discussed in a review article by Kato [22], the saliva of sand flies is a complex mixture of molecules with anticoagulant, antiplatelet, vasodilator and anti-inflammatory effects on the host. Although some salivary components have been associated with exacerbation of *Leishmania* infection [44], host immunity to some salivary components has been shown to provide protection against *Leishmania* infection [5] (Belkaid *et al.*, 1998). A thought-provoking hypothesis was put forward by Pedro Cecilio (O8), who investigated whether repeated bites from uninfected sand flies could reactivate cutaneous leishmaniasis (CL) lesions in rodent models. Most forms of leishmaniasis are zoonotic, so animal reservoirs play an important epidemiological role in leishmaniasis. The group used a rodent model for CL, as these animals are important reservoirs in nature. When uninfected sand flies were allowed to bite into healed CL lesions in the ears of mice, there was an increase in ear thickness and, most importantly, a significant increase in the parasite load in the ear of the exposed animals. Remarkably, greater pathological changes were observed when healed CL lesions were repeatedly exposed to the bites of uninfected *Ph. duboscqi* sand flies. The authors hypothesise that uninfected sand flies may play an important role in maintaining competent *Leishmania* reservoirs.

Although reproduction in *Leishmania* is primarily clonal, there is evidence of hybridisation within and between species, and hybrids have been obtained in the laboratory. Genetic exchange appears to be relatively rare in *Leishmania* and mainly occurs in the midgut of the sand fly [10]. The specific mechanisms involved in hybridisation are still unknown. Marcela Fuentes-Carias (O9) investigated the role of the nuclear fusion factor Gex1 in hybridisation of *Leishmania* and demonstrated that it is essential for mating both *in vitro* and potentially in sand flies. Using CRISPR/Cas9 *GEX1*null mutants and addback strains, it was shown that *GEX1*mutants do not generate hybrids. When only one parental strain expressed *GEX1*, hybrid frequency decreased but was not abolished, suggesting that *GEX1* is required in only one parental cell for successful hybrid production. *In vivo* experiments are now being carried out on sand flies. *GEX1* has been demonstrated to be essential for hybridisation of *Leishmania*, and tools have been developed that will allow the mechanism of action to be elucidated *in vitro* and *in vivo*.

Previous studies have shown the prevalence of CL caused by *L. major* or *L. tropica* and visceral leishmaniasis caused by *L. infantum* in Israel [42] (Studentsky *et al.*, 2023). However, in recent studies presented by Liora Studentsky (O10), collection of *Phlebotomus* spp. in the Negev Desert in southern Israel aimed at identifying circulating *Leishmania* spp. revealed that 80% of the specimens were *Ph. alexandri*, of which 2.5% were *Leishmania* DNA positive, with 92% of infections attributed to *L. donovani*. *Leishmania* DNA isolated from a patient with CL in this region was identical to *L. donovani* DNA, confirming the prevalence of *L. donovani* in southern Israel. This demonstrates the importance of rapid diagnosis and accurate identification of the species to prevent progression of the disease.

Similar results were reported by Suha Kenan Arserim (O11), who provided the first evidence of *L. donovani* transmission in Türkiye. In her study, *Ph. alexandri* was identified as a potential vector, with *L. donovani* DNA detected in sand fly pools, including a positive *Ph. alexandri* pool, the first such detection in the country.

These findings have significant implications for public health surveillance and vector control strategies across the Mediterranean.

## Sand fly behaviour and symbiotic interactions

The session presented the latest research findings on various topics, including host-seeking behaviour, chemical ecology, microbial symbionts and environmental influences on sand fly activity. They highlight the complex biological and ecological interactions that influence sand fly vector competence and provide potential tools to improve surveillance, control and understanding of leishmaniasis transmission. Contributions came from researchers from different biogeographical regions, the Neotropics, the Old World and Asia, and offered comparative insights into the complex interplay between sand flies, their environments, hosts, microbiota and symbionts.

Jeffrey Shaw (O12) opened the session by addressing the vector competence of Neotropical sand flies and critically examining the use of the term *anthropophilic* to describe their attraction to humans and their feeding behaviour. He argued that this behaviour is better characterised as opportunistic and modulated by environmental factors and proposed the term “*anthropportunism”* to describe cases where sand flies are accidentally attracted to and feed on humans [40]. He further suggested that the available information on infections and host attractions indicates that the main leishmaniasis vectors are generalists that feed on a wide range of hosts, including humans, when available.

Marcos Bezzera Santos (O13) used *Ph. perniciosus* to investigate the role of the olfactory system in host preference by testing electrophysiological responses to volatile organic compounds (VOCs) of dogs and humans [6] (Bezerra-Santos *et al.*, 2024). Nonanal was identified as a major attractant and provides new evidence for disruption of host-seeking and surveillance systems, particularly in vectors of *L. infantum* in the Old World. Orin Courtney (O14) then shifted the focus to chemical ecology and introduced the use of synthetic sex pheromones to understand the movements of *Lu. longipalpis*, the vector of visceral leishmaniasis (VL) in the Americas. This work began in the 1980s, and recent developments include the use of the pheromone in conjunction with CDC light traps in seven Brazilian states. This allows the presence of this vector to be monitored, which serves as the basis for an early warning system (EWS) for VL transmission.

Gideon Wasserberg (O15) focused on egg-laying behaviour and strategies to “attract-and-kill”. His multidisciplinary approach to *Ph. papatasi* oviposition reveals important chemical and visual cues that influence female site selection. Semiochemicals such as dodecanoic acid and isovaleric acid, derived from conspecifics and associated bacteria, were identified as strong attractants. These findings contribute to the development of targeted “attract-and-kill” traps for vector control.

The study by Dia-Eldin Elnaiem (O16) on the nocturnal biting behaviour of *Ph. orientalis* in Sudan provided new insights into the behavioural ecology and the influence of the lunar cycle. A significant correlation was observed between moonlight intensity and biting activity, with most sand flies landing and searching for hosts in the late-night hours. These findings have direct implications for optimising the timing of vector control measures.

A better understanding of the potential of biocontrol based on endosymbionts was obtained from studies on insects collected in the field. Amanda Rosário (O17) investigated *Wolbachia* prevalence in *Lu. longipalpis* populations from endemic areas in Brazil. The strains of the endosymbiont were identified using the 16S rRNA and surface protein genes. Although the overall infection rate was low (2.2%), the characterisation of these strains provides a basis for future applications of *Wolbachia* in biological control. Complementary to this work, Kentaro Itokawa (O18) analysed the genome of *Sergentomyia squamirostris* from Japan and reported on dual infections with *Wolbachia* and *Candidatus Tisiphia*. Their localisation in reproductive tissues and evidence of vertical transmission indicate stable symbioses. Although *Se. squamirostris* is not a confirmed vector of *Leishmania* to humans, these findings contribute to a better understanding of symbiont–host interactions in sand flies.

The last presentation focused on the control of paratransgenes. Thais Campolina (O19) presented a detailed metagenomic analysis of the midgut microbiota of *Lu. longipalpis* caught at a Brazilian locality in Minas Gerais, based on 16S rRNA gene sequencing. Several bacterial taxa, including *Lysinibacillus*, *Serratia* and *Pseudocitrobacter*, significantly inhibited the growth of *Leishmania* both *in vitro* and *in vivo*, highlighting their potential for paratransgenesis strategies to disrupt parasite development in the vector.

The presentations highlighted that the behaviour of sand flies is influenced by a combination of intrinsic and environmental factors. The identification of behavioural targets, the deciphering of environmental factors and the use of symbionts are central strategies for establishing a solid basis for innovative control and monitoring tools.

## Natural infection of sand flies by pathogens

The detection of natural infection with *Leishmania* parasites and viruses in sand flies is crucial for the identification of vectors and for the surveillance and control of the diseases. Although such studies have been conducted in many parts of the world, information remains sparse and limited to a relatively small number of disease foci. The fourth oral session of ISOPS XI included six presentations on field and laboratory studies of sand fly infection with *Leishmania* parasites and viruses in East Africa, Europe and Asia.

Mattia Calzolari (O20) investigated the presence of phleboviruses and *Leishmania* infections in sand flies collected between 2021 and 2023 at more than 250 different locations in the northern Italian regions of Lombardy and Emilia-Romagna. Using CO_2_-baited CDC light traps, the authors caught a total of 119,389 sand flies, which consisted mainly of *Ph. perniciosus* (65%) and *Ph. perfiliewi* (33%) in Lombardy, and *Ph. perfiliewi* (92%) in Emilia-Romagna. Molecular analysis of 2,051 pools revealed the presence of *Leishmania* parasites and Toscana virus in 294 and 78 samples, respectively. The authors also detected Fermo virus, Ponticelli virus and Sicilia virus in sand flies. *Leishmania* characterisation using multilocus microsatellite typing (MLMT) revealed two distinct subpopulations of *Leishmania* parasites circulating in the area studied.

Using artificial membrane infection, viral particle titration and RT-qPCR, Nikola Polanska (O21) analysed the susceptibility of *Ph. papatasi*, *Ph. tobbi*, *Ph. sergenti* and *Se. schwetzi* to Toscana virus (TOSV) lineages A and B (TOSV-A and TOSV-B). The results showed that *Ph. tobbi* is highly susceptible to TOSV-B and can potentially serve as a TOSV vector in the eastern Mediterranean region, especially where *Ph. perniciosus* and *Ph. perfiliewi*, which are currently documented as vectors of the virus in this region, are absent.

As part of India’s nationwide vector surveillance of *L. donovani*, Prasanta Saini (O22) collected sand flies using CDC-modified light traps and mechanical aspirators in five Indian zones (north, south, west, east and central India) in domestic and non-domestic habitats and screened them for *L. donovani* infection using molecular methods. The authors found that 18.31% of individuals and 40.27% of pools were positive for infection with the parasite and suggested that the distinct distribution pattern of certain sand fly species and the wide distribution of *Ph. argentipes* facilitate the transmission of leishmaniasis throughout the country.

Eva Iniguez (O23) presented results from surveys of VL and its vectors in the East African countries of Ethiopia, Kenya and Sudan, which represent the largest burden of the disease worldwide. The authors found rK39-positive human cases in both treated VL patients and asymptomatic individuals in Kenya and Sudan. Using qPCR on individually analysed sand flies, they found 13/310 (4.2%) *Ph. orientalis* in Kenya and 1/67 (1.5%) *Ph. martini*/*celiae* in Ethiopia infected with *L. donovani*. In Sudan, 1/5 of 10 (20%) *Ph. orientalis* pools were infected with the parasite. Infected specimens were mainly collected from vegetation, *e.g.* from acacia trees associated with vertisol cracks and animal burrows in Kenya, from a termite mound in Ethiopia and near animal enclosures in Sudan, suggesting that transmission is focal and occurs in different microhabitats in the three countries.

Mariaelista Carbonara (O24) conducted a field study on *L. infantum* and *L. tarentolae* infection in unproven vectors (*i.e. Se. minuta* and *Ph. perniciosus*, respectively) at six sampling sites in southern Italy. The collected sand flies were identified as *Ph. perniciosus*, *Ph. perfiliewi* (*n* = 92), *Ph. neglectus* (*n* = 16) and *Se. minuta* (*n* = 259). Although flagellates were not observed in dissected females, *L. infantum* DNA was detected in specimens of *Ph. perniciosus* (*n* = 3/182; 1.6%), *Ph. perfiliewi* (*n* = 1/66; 1.5%) and *Se. minuta* (*n* = 5/248; 2%). In addition, DNA of the non-pathogenic *L. tarentolae* was detected in *Se. minuta* (*n* = 11/248; 4.4%). The authors suggested that when *L. infantum* and *L. tarentolae* occur in sympatry, the herpetophilic *Se. minuta*, the proven vector of *L. tarentolae*, may also be infected by *L. infantum* and feed on mammals.

Khalil Dachraouj (O25) conducted a study on *L. infantum* and Toscana virus infection in sand flies and dogs in a canine leishmaniasis focus in northern Tunisia. During the study period, sand flies were collected weekly with sticky traps and identified to species level. *Phlebotomus perniciosus* was the most abundant species (41.94%), followed by *Ph. perfiliewi* (31.36%), *Se. minuta parotti* (26.19%), *Ph. papatasi* (0.36%) and *Ph. longicuspis* (0.12%). The phenology of sand flies of the subgenus *Larroussius* showed two main peaks: a small one in June and a larger one in September–October. The infection rates of sand flies with *L. infantum* and TOSV were 0.053% (2/3730) and 0.09% (6/6211), respectively. TOSV-positive pools were detected in both female and male sand flies. The authors noted that *L. infantum* and TOSV were detected during the second main peak of sand fly activity. Furthermore, xenodiagnosis experiments showed that both *L. infantum* and TOSV RNA viruses are transmitted from dogs to *Ph. perniciosus* females. The results provided clear evidence that in addition to their role as the main reservoir for *L. infantum*, dogs also act as reservoir hosts for Toscana virus.

## Host and reservoir identification and sand fly control

**Figure 1:**
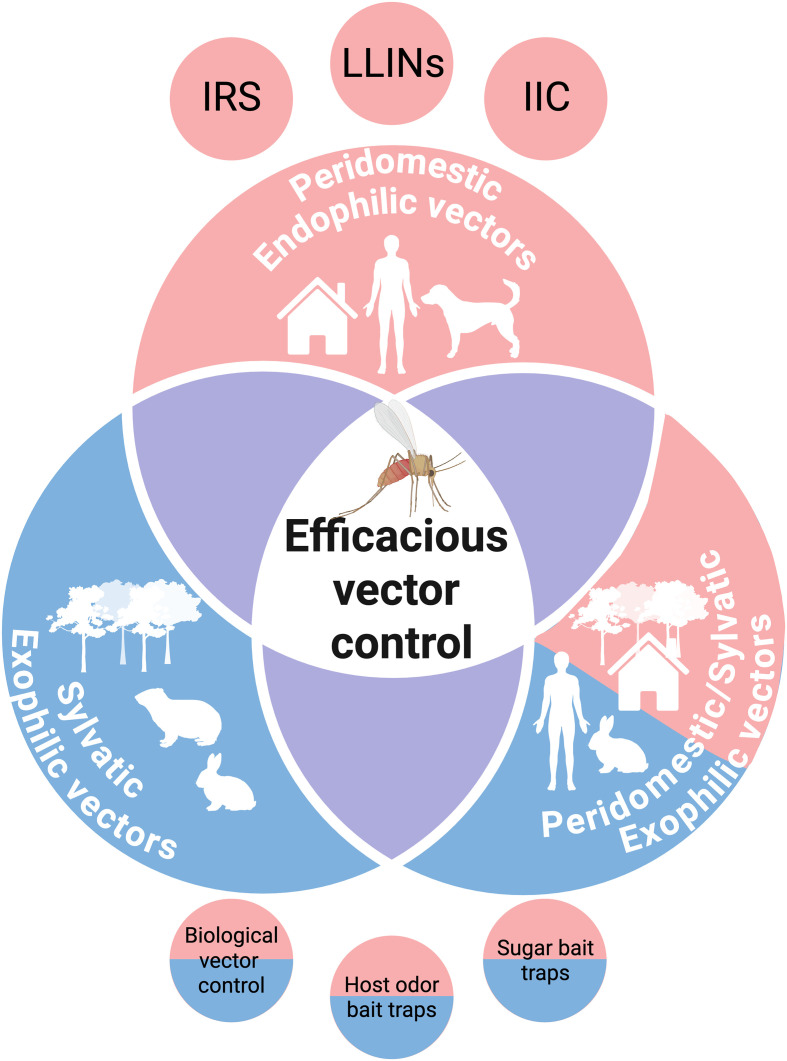
*Understanding transmission dynamics for the effective implementation of vector control*. In a peridomestic transmission cycle, endophilic vectors feed on disease reservoirs such as humans and dogs. In this scenario, vector control strategies such as IRS, LLINs and IIC are effective. In a sylvatic transmission cycle, exophilic sand flies feed on wild animals. In this case, innovative control tools such as insect bacterial commensals and host odour/sugar bait traps are required. In mixed peridomestic and sylvatic transmission cycles, where the vector is exophilic, the use of IRS, LLINs and IIC may be ineffective, requiring integration of control approaches to effectively interrupt transmission. The purple areas represent zones where there may be spillover between the projected scenarios due to potential changes in human and animal movement, behaviour and environmental drivers such as climate change. IRS: Indoor residual spraying; LLINs: Long-lasting insecticide-treated nets; IIC, Insecticide-impregnated collars. This figure was created with BioRender. Iniguez, E. (2025) https://BioRender.com/l2d3gpc.

The prevalence of CL and VL in zoonotic and anthroponotic transmission cycles is maintained by different epidemiological conditions (Fig. 1) [4, 24]. Nevertheless, it is known that leishmaniasis is mostly a zoonosis involving animals whose identity depends on the nature and biodiversity of the disease foci. Currently, only VL caused by *L. donovani* is still considered an anthroponosis [24].

Animal reservoirs are well characterised in certain foci and their diversity is exemplified by the incrimination of animals such as hyraxes and hares in CL and VL transmission [29, 37]. More established reservoirs include dogs, which are considered the main source of human infection with *L. infantum*, and *Psammomys obesus* and *Rhombomys opimus*, established principal reservoirs of *L. major*, all of which maintain peridomestic and sylvatic zoonotic transmission in different environments [8, 49]. However, in many regions of East Africa and Southeast Asia, the presence of reservoirs is still largely unknown. As such, unidentified reservoirs represent one of the most significant knowledge gaps in leishmaniasis research and hinder the development of effective vector control strategies to interrupt disease transmission.

At ISOPS XI, Shaden Kamhawi (O26) presented an innovative, field-applicable toolkit for identifying leishmaniasis reservoirs that focuses on in-depth analysis of DNA and RNA co-extracted from single blood-fed sand fly midguts. Kamhawi and colleagues showed that an adaptable combination of molecular tools can distinguish a first infected blood meal from subsequent blood meals through differences in gene expression of early and late parasite populations. In combination with the identification of blood meals by customised multiplex PCR and *CytB* sequencing, this toolkit can provide an accurate assessment of the identity of a reservoir animal. Although the toolkit is promising, it needs to be further validated in different field environments.

In the same session, Bruno Oliveira Cova (O27) and Sofia El Kacem (O28) presented alternative approaches for blood meal analysis of sand flies collected in the field. Bruno Oliveira Cova and his colleagues used next generation sequencing of three barcoded genes, including a broad vertebrate-specific 12S rRNA, a mammalian-specific 16S rRNA and the mitochondrial *CytB* gene. The authors demonstrated that sand flies feed on mammals and other hosts such as rodents and ungulates, and less frequently on reptiles and amphibians. Sofia El Kacem and colleagues used proteomics to identify the source of blood meals of the sand fly vector *Ph. sergenti* in an endemic leishmaniasis area in northern Morocco. The authors created a reduced in-house library of blood protein sequences enriched for potential Moroccan hosts. The library allowed rapid data comparison with proteins obtained from fed sand flies after analysis by liquid chromatography coupled with tandem mass spectrometry. Creating custom libraries of proteins or genes from animals suspected to be in the areas of interest is an effective tool to facilitate the identification of reservoir hosts.

Most studies focus on screening DNA from field-collected sand flies for *Leishmania* infection using high copy targets, in parallel or not with the identification of host blood meals [15, 23]. We believe that more attention should be paid to RNA screening for the detection of live parasites. Recent studies using RT-PCR in experimentally infected sand flies confirmed the expression of *sherp* as a stage-enriching metacyclic gene and it was suggested as a surveillance tool to predict transmission potential [33]. However, in this and other studies [17], this gene was not examined in the context of multiple blood meals given at 5- to 6-day intervals to mimic the natural feeding behaviour of sand flies, which is known to play a central role in parasite development and vector competence of sand flies and several other vectors [11].

This session at ISOPS XI focused on presentations of vector control tools, highlighting current preventive measures such as long-lasting insecticide-treated nets (LLINs) and dog collars. Mara Cristina Pinto (O30) investigated the efficacy of Interceptor^®^ G1 (alpha-cypermethrin) and Interceptor^®^ G2 (chlorfenapyr and alpha-cypermethrin) LLINs, commonly used against malaria [9], against sand flies. All sand flies were killed when exposed to G1 LLINs, while 40% survived exposure to G2 LLINs, although their feeding and oviposition behaviour were impaired. These preliminary results are important in view of the recommended implementation of G2 LLINs to control insecticide-resistant vectors [32, 41].

The emergence of insecticide resistance in sand flies poses a major challenge for global leishmaniasis control [3]. In Brazil, dog collars impregnated with deltamethrin are used as part of the national programme to control VL [35]. Rafaella Albuquerque e Silva (O31) demonstrated that sand flies from four of six endemic municipalities in Brazil, including Montes Claros in the state of Minas Gerais, were susceptible to deltamethrin, with one municipality classified as resistant and another as possibly resistant [13]. In another study, Fredy Galvis-Ovallos (O32) reported that dog collars impregnated with 4% deltamethrin had no effect on sand fly density in Montes Claros/MG and only a slight, non-significant effect on sand fly engorgement. Overall, these two studies suggest that the wide use of deltamethrin-impregnated dog collars may accelerate the emergence of insecticide resistance without affecting vector populations. Further studies are needed to evaluate the proper use of dog collars on a large scale in Brazil. Kardelen Yesilmis (O33) reported prolonged knockdown values for 0.05% deltamethrin and susceptibility to 1% permethrin in field-collected sand flies from Türkiye, further emphasising the importance of integrating resistance management strategies in sand fly control. In particular, the implementation of sand fly control measures, such as spraying with insecticides, LLINs and collars, have a significant impact on the control of sand flies in peridomestic disease foci [25, 34] (Fig. 1).

For biological control of sand flies, Marketa Stejskalova (O29) investigated the effect of bacterial commensals such as *Asaia siamensis* and *Asaia krungthepensis* by feeding the bacteria to *Ph. duboscqi* sand flies in their sugar meal. The group reported that *Asaia* spp. had a negative effect on the late stages of infection after infection with *L. major*. Another study showed that ingestion of *Delftia tsuruhatensis* TC1 bacteria by sand flies caused an intestinal dysbiosis that was detrimental to the development of *L. major* in *Ph. duboscqi*, with a significant reduction in midgut parasite load compared to control flies; this phenotype was maintained even after giving a second blood meal to rescue the infection. Importantly, sand flies fed with *D. tsuruhatensis* were 73% less efficient at transmitting and causing disease in mice compared to the control group [12]. These methods offer a major advantage as they can easily be used in both sylvatic and peridomestic niches, compared to indoor residual spraying and treated nets and collars, which mainly affect transmission in peridomestic niches. Ideally, vector control tools should be developed to target both scenarios and their deployment should be based on knowledge of transmission dynamics in the target focus (Fig. 1).

In summary, current advances in molecular surveillance together with innovative control methods promise to improve integrated vector control management. A comprehensive and versatile approach, specifically tailored to sylvatic or peridomestic transmission, is essential for the interruption of leishmaniasis. Filling the gaps in our understanding of leishmaniasis transmission, particularly the identification of infection reservoirs, is critical to the development of such targeted and effective control strategies.

## Climate-driven disease risk and EWS development

In a special session on the CLIMOS project (Climate Monitoring and Decision Support Framework for the Detection and Mitigation of Sand fly Diseases with Cost-Benefit and Climate Policy Measures; http://www.climosproject.eu), a multidisciplinary approach to EWSs for sand fly-borne infections and diseases was presented, enabling better preparedness for current and future impacts of climate and environmental change on human and animal health. Carla Maia and Suzana Blesić (O34) presented the framework for the integration of environmental and epidemiological data related to the presence and abundance of sand flies and the infection rate in humans and domestic animals at different geographic scales across Europe and neighbouring countries, while Vit Dvorak, Jorian Prudhomme and Gioia Bongiorno (O35–O36) presented detailed protocols for sampling and pathogen detection. From April to November 2023, coordinated and standardised sampling for the collection and processing of sand flies was carried out in 11 countries. A total of 117 sampling sites in Portugal, Spain, France, Italy, Slovenia, Croatia, Germany, Austria, Czechia, Türkiye and Israel were examined using CDC light traps for temporal data collection. Twenty-two sand fly species of the genera *Phlebotomus* (species of 7 subgenera) and *Sergentomyia* were recorded in the countries studied. In order to obtain comparable data on the detection in the sand flies studied, two external quality assessments were carried out at the participating partners, one focussing on phleboviruses [2] and the other on *Leishmania* [36]. The collected sand flies were then analysed for the presence of sand fly-borne pathogens using the standardised detection protocols. Jovana Sadlova (O37) described the experimental work being conducted to assess the vector competence of European sand flies against emerging *Leishmania* species in Europe and Toscana virus (TOSV). The experiments confirmed that *Ph. perniciosus* and *Ph. tobbi* are competent vectors for *L. donovani* and *L. major*, but refractory to *L.* (*Mundinia*) *martiniquensis* [38]. Experiments with wild-caught *Ph. perfiliewi* confirmed its susceptibility to *L. tropica*. *Phlebotomus tobbi* has been shown to support the development of TOSV [20].

Technological innovations played an important role. Orin Courtenay (O38) presented a semiochemical-based remote monitoring device, a simple, affordable and sensitive trap for detecting key sand fly vectors. Semiochemicals will be firstly tested as potential attractants for *Ph. perniciosus*, *Ph. papatasi* and *Lu. longipalpis* sand fly colonies in laboratory experiments using a Y-tube olfactometer. Candidate compounds identified in the laboratory will then be evaluated under field conditions in Spain, Italy, Türkiye and Portugal. There are also plans to develop a prototype monitoring device that combines the chemicals on sticky traps with a widely available high-quality camera and IoT modules for automatic remote counting of trapped sand flies. Since host antibody levels against sand fly salivary antigens reflect the intensity of host exposure to sand fly bites and can be used as a marker of exposure [26], Iva Kolarova (O39) presented the development of recombinant salivary antigens to be used to measure exposure to vector bites in sentinel dog populations from Portugal, Spain, Italy and Türkiye as an early warning surveillance tool for circulating *Leishmania* parasites and to evaluate the effectiveness of the protective measures developed. Vladan Gligorijević and Sergio Natal (O41–O42) presented user-friendly visual tools and EWS interfaces.

Yoni Waitz (O40) presented the work carried out during a project on data analysis and modelling of climate-induced sand fly dynamics. This included the presentation of the preliminary results of environmental niche modelling [47] to explain and predict sand fly presence in Europe, Türkiye and Israel, as well as an introduction to time series analysis of long-term sand fly population trends. The time series analysis mainly involves the use of wavelet transform spectral analysis (WTS) to understand the intrinsic temporal dynamics of sand fly data (from monitoring) and the patterns and time intervals of their cross-correlations with meteorological and soil moisture data, which are studied as their climatic and environmental drivers. Wavelet analysis has been shown in the past to be a very useful tool for understanding and modelling climatic factors for vector-borne diseases [21]. In particular, the use of the local wavelet cross-correlation functions (lcWTS) provides clear insight into the time lags between changes in climate and environmental drivers and changes in sand fly density, on the one hand, and the occurrence of these changes in real time, on the other [45], allowing additional real-time tracking of other variables of interest as well. The data used for the presented lcWTS analysis within the CLIMOS project include historical sand fly monitoring records from Portugal, Israel and Greece (Crete) as well as meteorological data (average temperature and precipitation) and soil moisture data from the same locations. The meteorological data were taken from the ERA5 Land dataset and downscaled to 1 km x 1 km averages over the trap sites, while the soil moisture data were taken from the mHM hydrological model at the same spatial resolution [16]. The analysis approach and the preliminary results presented included two steps. First, the group examined the lcWTS spectra of sand fly numbers and selected hydro-meteorological drivers for a single sand fly species at different geographical locations to confirm whether the behaviour of a single species is universal or not. Subsequently, the lcWTS spectra for different sand fly species at the same and different geographical locations were examined to confirm whether the behaviour is or is not universal for different species and different locations. These results will be complemented with the findings from the land-use data, which could explain the variability in sand fly abundance that is not due to the observed dependence on hydroclimatic drivers. The aim of the CLIMOS project is to investigate, understand and model, through data analysis and modelling, the universal relationship between sand fly abundance and hydro-meteorological and environmental variables at the species level and, if possible, at the level of all species, to use the same approach to model the presence of pathogens for sand fly-borne diseases and finally to project all these variables with climatic and environmental data.

At the end of this session, Diana Guardado (O43) emphasised the role of public engagement in promoting the adoption of EWS.

## Sand fly taxonomy, distribution and surveillance

A rich series of presentations emphasised recent advances in taxonomy and monitoring of sand flies. Jérôme Depaquit (O50) reviewed the complex taxonomy of Southeast Asian sand flies, while Huicong Ding (O51) and Thanapat Pataradool (O52) described species new to science from Singapore and Thailand. Khamsing Vongphayloth (O53) and Stavroula Gouzelou (O54) reported unexpected species diversity in Laos and Cyprus.

This session highlighted that many sand fly species remain undescribed in Southeast Asia. Even in highly urbanised areas such as Singapore, where mosquito surveillance is routine, Huicong Ding (O51) recorded the country’s first Phlebotomine sand flies and described four new species hidden in plain sight. In addition, underexplored regions continue to reveal unrecognised diversity. Jérôme Depaquit (O50) underlined the importance of sharing biological material and coordinating research to resolve species complexes such as the *Se. barraudi* group, the *Se. iyengari* group and *Ph. kiangsuensis*. A revision of Southeast Asian sand fly taxonomy is expected, involving collaborations with colleagues from China and India, where many species were originally described.

Surveillance efforts in Europe were presented by Alessandro Alvaro and Ina Hoxha (O55–O56), while Fátima Amaro (O57) described Portugal’s national sand fly network. Eduardo Berriatua (O58) highlighted the large-scale, standardised surveillance carried out within the CLIMOS project across Europe and neighbouring countries. In Spain, sampling was conducted at 23 localities on two consecutive nights each month between April and November 2023. More than 6,600 specimens were collected, representing six species, with *Ph. perniciosus* the dominant one. Several proven and suspected *Leishmania* vectors were detected, with marked variation in species composition and density across regions. Results demonstrated strong seasonality and dependence on climatic and environmental conditions at both local and regional scales.

In Tunisia, Jamila Ghraib (O59) investigated the sand fly fauna in a focus of CL in Sidi Bouzid. *Phlebotomus papatasi* was the dominant species, found to be abundant indoors and in rodent burrows, confirming its role as the main vector of *L. major*. However, the diversity of *Sergentomyia* spp. was notable, with five species recorded, including one unidentified taxon. qPCR detection of *Leishmania* DNA not only confirmed infections in *Ph. papatasi*, but also revealed positives in two *Sergentomyia* species, raising once again the question of their possible involvement in human transmission.

From Algeria, Kamal Benallal (O60) presented results from several transmission foci in the northern and central Sahara. Using an integrative approach combining morphology, sequencing and matrix-assisted laser desorption/ionization time-of-flight (MALDI-TOF) MS protein profiling, eight species of *Sergentomyia* and *Phlebotomus* were identified. This work led to the description of a new species, *Se. imihra*, demonstrating the added value of integrative taxonomy [7].

The usefulness of MALDI-TOF MS for taxonomy was further illustrated by Petr Halada (O61) in the *Ph. perniciosus/longicuspis* complex. This group includes two morphotypes of *Ph. perniciosus* (typical PN and atypical PNA), which differ in male genitalia but not in females, and two distinct species of *Ph. longicuspis* (typical LC and atypical LCx), one undescribed. Their morphological similarity complicates species identification and obscures distribution and epidemiological roles, particularly in endemic areas of the Maghreb. Protein profiling of specimens from Ouazzane province, Morocco, where all members occur sympatrically, allowed reliable discrimination of both males and females. These results were consistent with *CytB* gene sequencing, highlighting MALDI-TOF MS as a powerful, potentially high-throughput tool for routine identification.

Unexpected ecological findings were reported by Ognyan Mikov (O62). Studies in Orlova Chuka cave, Bulgaria, revealed overwintering and possibly breeding populations of *Ph. neglectus* living in complete darkness and feeding on bats, whose guano may support larval development.

Citizen science was also shown to contribute. Milagres Tascísio (O63) introduced the FLEBOCOLLECT project (https://www.flebocollect.com/), which engaged more than 1,900 participants in Spain to collect sand flies using do-it-yourself (DIY) traps. Four species were recorded, and the results were displayed in a public monitoring map. The project illustrates how simple tools, combined with mobile applications and educational materials, can expand surveillance and awareness of sand fly-borne pathogens.

Further taxonomic contributions included work by Bruno Rodrigues (O64), who demonstrated hidden diversity within the genus *Pintomyia* (subgenus *Pifanomyia*, Monticola series) using morphometrics. Katharina Platzgummer (O65) and Jorian Prudhomme (O66) reviewed sand fly distributions in Central and Western Europe, showing range expansions linked to climate change. Finally, Ilaria Bernardini (O67) presented evidence from Tuscany, Italy, supporting the role of *Ph. perfiliewi* in the transmission of *L. infantum* and TOSV.

## Conclusions and prospects

The 11th International Symposium on Phlebotomine Sand flies (ISOPS XI) showcased the remarkable breadth of current sand fly research, ranging from laboratory experiments to taxonomy, ecology, field monitoring and control strategies. The various sessions of the Symposium emphasised both the scientific complexity of sand fly biology and the urgent public health importance of sand fly-borne diseases.

A central theme of ISOPS XI was the recognition that climate change and environmental pressures significantly influence the spread of vectors and the incidence of vector-borne diseases. Several presentations highlighted the northward expansion of suitable sand fly habitats in Europe, the emergence of novel vector-pathogen associations and the role of ecological niches and microhabitats in shaping transmission dynamics. These findings challenge traditional paradigms of leishmaniasis epidemiology and emphasise the need for adaptable and flexible surveillance systems.

The integration of molecular biology, omics technologies and ecological modelling is transforming the field. Advances in genomics, transcriptomics and proteomics, coupled with modelling approaches such as environmental niche modelling and wavelet analysis, are providing new insights into vector competence, pathogen evolution and environmental determinants of transmission. New research on the microbiota and symbionts as well as on hybridisation and sexual reproduction of *Leishmania* has broadened the spectrum of investigations and opened up new avenues for intervention.

Progress has also been made in diagnostics and control. Sensitive molecular assays, recombinant saliva biomarkers and the use of insecticide-treated dog collars and nets offer promising tools for early detection and prevention. At the same time, reports of insecticide resistance emphasise the urgent need for integrated vector management strategies and resistance monitoring to ensure effectiveness.

The CLIMOS project was an outstanding example of how transdisciplinary and cross-sectoral collaboration can be used to tackle these challenges. By combining climate science, entomology, epidemiology, public health and citizen engagement, CLIMOS has pioneered a framework for an operational EWS for sand fly-borne diseases. Such collaborative models will be essential for preparing and mitigating the effects of global climate change.

**Figure 2:**
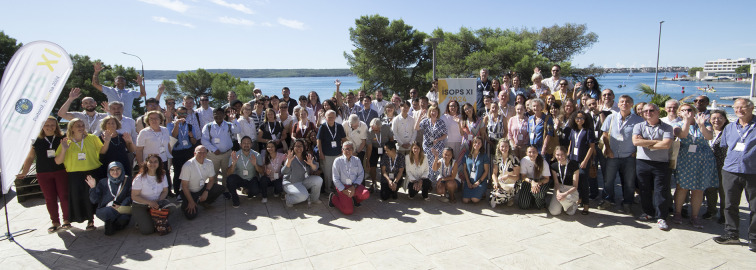
Photo of ISOPS XI participants.

Looking to the future, several priority areas for research and policy were highlighted in ISOPS XI discussions (Fig. 2):

- Standardisation of vector competence assays and transmission models.
- Greater inclusion of non-traditional vectors and reservoir hosts in surveillance.
- Expansion of long-term, cross-border and citizen science surveillance initiatives.
- Strengthening of institutional and community capacity for the development and implementation of early warning systems.
- Continued application of integrative taxonomy and novel tools (*e.g.* MALDI-TOF MS) to resolve species complexes of epidemiological importance.

ISOPS XI again confirmed the importance of sustained international co-operation and knowledge exchange. Future symposia will continue to be important platforms to guide science, inform policy and coordinate global responses to sand fly-borne diseases.

Following the alternation rule between the Old and New Worlds, ISOPS XII will be held in Brazil in 2028, hosted by the Universidade Federal de Mato Grosso do Sul, Laboratório de Parasitologia Humana-INBIO (contact person: Alessandra Gutierrez de Oliveira).

## Supplementary Files

ISOPS XI programme and abstract book. The supplementary material of this article is available at https://www.parasite-journal.org/10.1051/parasite/2025062/olm.
