# Supplementary material for: Recent advances in Phlebotomine sand fly research: a review based on studies presented at ISOPS XI
Source: Parasite. 2025 Oct 29;32:69. doi: 10.1051/parasite/2025062 (PMC12571480; doi:10.1051/parasite/2025062)
Supplement: Supplementary file 1 — ISOPS XI programme and abstract book. [file parasite-32-69-s1.pdf]

ISOPS XI

Portorož, Slovenia 2024

PROGRAMME AND ABSTRACT BOOK

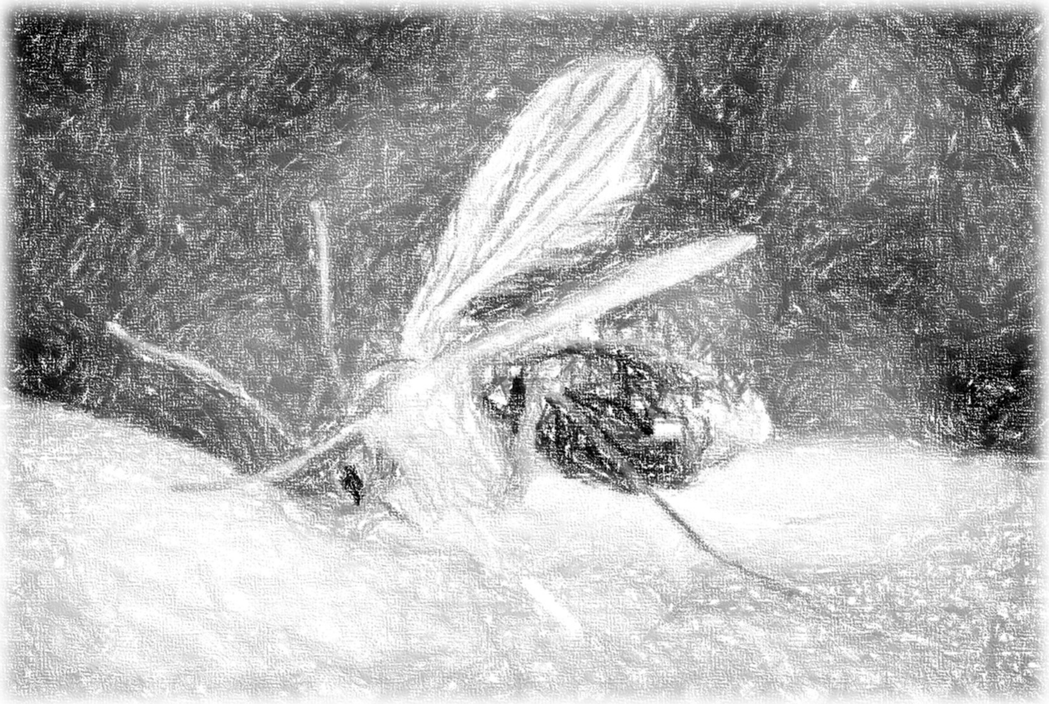

ISOPS XI
International Symposium on Phlebotomine Sandflies
9-13 September 2024. Portorož, Slovenia

Edited by Vladimir Ivović

Revised by Members of the Scientific Programme Committee

Technical support Katja Adam, Alenka Babič, Sanja Sandić, Tanja Labus

Cover photo: CDC/PHIL - 10277

Organised by Department of Biodiversity, Faculty of Mathematics, Natural Sciences and Information Technologies, University of Primorska - Koper, Slovenia

&

Society of Conservation Biologists (BIODIVA) - Koper, Slovenia

Koper, Slovenia | September 2024

©2024 Authors

Printing sponsored by BIODIVA

Print-run

Honorary Committee

Michele Maroli (Italy)
Oscar Daniel Salomon (Argentina)
Yusuf Özbel (Turkey)
Jérôme Depaquit (France)
Sonia Elizabeth Zapata Mena (Ecuador)
Nicole Léger (France)
Jeffrey Shaw (Brazil)

Scientific Committee

Eunice Galati (Brazil)
Petr Volf (Czech Republic)
Shaden Kamhawi (USA)
Carla Maia (Portugal)
Nagila Secundino (Brazil)
Mary Cameron (UK)
Meryem Lemrani (Morocco)
Salih Bulent Alten (Turkey)
Bongiorno Gioia (Italy)
Reginaldo Brazil (Brazil)

Organizing Committee

Vladimir Ivović (Slovenia)
Katja Adam (Slovenia)
Jure Jugovic (Slovenia)
Sara Zupan (Slovenia)
Ozge Erisoz Kasap (Turkey)
Vit Dvorak (Czech Republic)

WELCOME ADDRESS

Esteemed colleagues and honoured guests,

It is a great honour and pleasure to welcome you all to the 11th International Symposium on Phlebotomine Sandflies (ISOPS XI). We are thrilled to have gathered such a distinguished assembly of scientists and practitioners from around the world, united by our shared commitment to advancing the field of phlebotomine sandfly research.

Research on phlebotomine sandflies, the exclusive vectors of leishmaniasis, is now more important than ever. Given the unprecedented changes in our global climate, robust and innovative research on these vectors and the diseases they transmit is essential. Climate change is altering ecosystems, expanding the habitats of these vectors and consequently increasing the incidence and spread of leishmaniasis and other vector-borne diseases. Understanding the dynamics of phlebotomine sandflies in this changing environment is crucial for the development of effective control and prevention strategies.

This symposium provides an important platform for the exchange of knowledge, ideas and the latest research findings. Only through our collective efforts can we advance science and develop innovative solutions to the challenges posed by phlebotomine sandflies and the pathogens they transmit. Your presence here is a testament to your commitment to this cause and your contributions are invaluable.

I would like to express my deepest gratitude to the members of the Organising committee: Katja Adam, Sara Zupan, Jure Jugovic, Vit Dvorak and Ozge Erisoz Kasap, whose hard work and dedication have made this Symposium possible. Their tireless efforts in planning and coordination have ensured that we have a conducive environment for fruitful discussions and networking.

Once again, welcome to ISOPS XI. I look forward to the insightful presentations, stimulating discussions and new collaborations that will result from this meeting. Together we can make significant progress in the field of phlebotomine sandfly research and public health.

Thank you and have a wonderful time in Slovenia,

Vladimir

ISOPS XI PROGRAMME & TIMETABLE

9 MONDAY

14:00 – 19:00	**REGISTRATION (ISOPS XI venue)**
19:00 – 21:00	**WELCOME COCKTAIL (News Café Bernardin)**

10 TUESDAY

8:45 – 9.15	**ISOPS XI OPENING**
9:15 – 10.30	**Experimental models of *Leishmania* transmission**<br>Chair: Gioia Bongiorno

O1	9:15 – 9.30	Márcia Laurenti	*In vitro* and *in vivo* experimental model of atypical cutaneous leishmaniasis by *Leishmania infantum* using salivary gland homogenate of *Lutzomyia longipalpis*
O2	9:30 – 9:45	Kristýna Jelínková	The effect of *Phlebotomus duboscqi* saliva on the ongoing *Leishmania major* infection in a murine model
O3	9:45 – 10:00	Tomáš Bečvář	Experimental transmission of *Mundinia* by biting midges and sand flies
O4	10:00 – 10:15	Sarah Hendrickx	Application and optimization of a *Lutzomyia longipalpis* transmission model to initiate various forms of cutaneous leishmaniasis in laboratory animals
O5	10:15 – 10:30	Petr Volf	Mechanisms of *Leishmania* attachment to the sand fly midgut and the stomodeal valve

**10:30 – 11:00**		**COFFEE BREAK**	
**11:00 – 12.30**		**Advances in *Leishmania* Research**<br>Chair: Yara Traub-Cseko	
**O6**	**11:00 – 11:15**	Pedro Cecilio	*Leishmania* transmission is disrupted in sandflies colonized by *Delftia tsuruhatensis* TC1 bacteria
**O7**	**11:15 – 11:30**	Aida Bouratbine	Contribution of real-time PCR targeting kinetoplast DNA to access sandfly infection rate in areas with low Leishmania infantum transmission
**O8**	**11:30 – 11:45**	Fabiano Oliveira	Are bites of non-infected sand flies important for the maintenance of cutaneous leishmaniasis in animal reservoirs?
**O9**	**11:45 – 12:00**	Marcela Fuentes Carias	*Leishmania* hybridization in their sand fly vectors: investigation of the role of Gex1
**O10**	**12:00 – 12:15**	Liora Studentsky	New evidence indicating the endemic transmission of *Leishmania donovani* in Israel
**O11**	**12:15 – 12:30**	Suha Kenan Arserim	The first detection of *Leishmania donovani* DNA in Türkiye, in its proven vector *Phlebotomus alexandri*
**12:30 – 14:00**		**LUNCH (News Café Bernardin)**	
**14:00 – 16:00**		**Sand fly behaviour & Symbiotic interactions**<br>Chair: Jeffrey Shaw	
**O12**	**14:00 – 14:15**	Jeffrey Shaw	Neotropical sand fly anthropophily – a natural or unnatural behaviour
**O13**	**14:15 – 14:30**	Marcos Antonio Bezzera Santos	Electrophysiological and behavioural responses of *Phlebotomus perniciosus* to volatile organic compounds of dogs and humans
**O14**	**14:30 – 14:45**	Orin Courtenay	The efficacy of a synthetic sex-aggregation pheromone to detect *Lutzomyia longipalpis*

**O15**	**14:45 – 15:00**	Gideon Wasserberg	Oviposition ecology of *Phlebotomus papatasi* sandflies: patterns, processes, and applications
**O16**	**15:00 – 15:15**	Dia-Eldin Elnaiem	Effects of the Lunar Cycle on the Nocturnal Activity rhythm and Hourly Man-biting rates of *Phlebotomus orientalis*, the vector of visceral leishmaniasis in Sudan
**O17**	**15:15 – 15:30**	Amanda Andrade do Rosário	Assessing *Wolbachia* circulation in field populations of Lutzomyia *longipalpis* in and endemic area of visceral leishmaniasis in Brazil
**O18**	**15:30 – 15:45**	Kentaro Itokawa	Dual infection of *Wolbachia* and *Candidatus* Tisiphia to *Sergentomyia squamirostris* in Japan
**O19**	**15:45 – 16:00**	Nagila Secundino	Role of the microbioma in *Lutzomyia longipalpis* Vector competence: What do we know?
	**16:00 – 16:30**	**COFFEE BREAK**	
	**16:30 – 18:00**	**Natural Infection of sand flies by pathogens**<br>Chair: Dia Elnaiem	
**O20**	**16:30 – 16:45**	Mattia Calzolari	Phleboviruses and *Leishmania* detection in sandflies: three years of monitoring in Lombardy and Emilia-Romagna Regions (Northern Italy)
**O21**	**16:45 – 17:00**	Nikola Polanská	Susceptibility of various sand fly species to Toscana virus
**O22**	**17:00 – 17:15**	Saini Prasanta	Detection of natural infection of *Leishmania donovani* among wild caught phlebotomine sandflies in India: an endemic focus of Leishmaniasis
**O23**	**17:15 – 17:30**	Eva Iniguez	Comparative analysis of *Leishmania*-infected sand flies in their microhabitats reveals the complexity of visceral leishmaniasis transmission in East Africa
**O24**	**17:30 – 17:45**	Mariaelisa Carbonara	Sympatric occurrence of *Leishmania infantum* and *Leishmania tarentolae* in *Sergentomyia minuta* sand flies

**O25**	**17:45 – 18:00**	Elyes Zhioua	Co-circulation of *Leishmania infantum* and Toscana virus in sandflies and dogs in a focus of canine leishmaniasis of Northern Tunisia

11 WEDNESDAY

		**FREE DAY**
		**EXCURSIONS**
**1.**	**9:00 – 17:00**	**ŠKOCJAN CAVES - LJUBLJANA**
**2.**	**9:00 – 17:00**	**LJUBLJANA - BLED**

12 THURSDAY

**8:30 – 10:30**		**Host and reservoir identification & Sand fly control**<br>Chair: Shaden Kamhawi	
**O26**	**8:30 – 8:45**	Shaden Kamhawi	An innovative toolbox aims to identify leishmaniasis reservoirs through analysis of individual field-collected blood fed sand flies
**O27**	**8:45 – 9:00**	Bruno Oliveira Cova	Invertebrate-derived DNA (iDNA) to identify sandflies' bloodmeal
**O28**	**9:00 – 9:15**	Sofia El Kacem	Establishment of an In-house library for host blood identification and blood meal analysis of a natural population of Moroccan *Phlebotomus sergenti* using a proteomic approach
**O29**	**9:15 – 9:30**	Marketa Stejskalova	*Asaia* bacteria in sand flies and their impact on *Leishmania* transmission

**O30**	**9:30 – 9:45**	Mara Cristina Pinto	Evaluation of biological parameters of female *Lutzomyia longipalpis* (Diptera:Psychodidae) after contact with insecticide-impregnated screens
**O31**	**9:45 – 10:00**	Rafaella Albuquerque e Silva	Insecticide impregnated collars for the control of visceral leishmaniasis: evaluation of the susceptibility of *Lutzomyia longipalpis* to deltamethrin
**O32**	**10:00 – 10:15**	Fredy Galvis Ovallos	Assessment of the impact of deltamethrin-impregnated collars on density of *Lutzomyia longipalpis* in an endemic area of visceral leishmaniasis in Brazil
**O33**	**10:15 – 10:30**	Kardelen Yetismis	Preliminary results of Insecticide resistance bioassay against synthetic pyrethroids on sandflies in west part of Türkiye
**10:30 – 11:00**		**COFFEE BREAK**	
**11:00 – 12:30**		**POSTER SESSION**	
**12:30 – 14:00**		**LUNCH (News Café Bernardin)**	
**14:00 – 16:00**		**CLIMOS project - Climate Monitoring and Decision Support Framework for Sand Fly-borne Diseases Detection and Mitigation**<br>Chair: Ozge Erisoz Kasap	
**O34**	**14.00 – 14:15**	Carla Maia & Suzana Blesić	Providing a better knowledge and comprehension of climate and environmental drivers of sand fly-borne diseases - the CLIMOS project
**O35**	**14.15 – 14:25**	Vit Dvorak	Sand fly sample collection and analysis
**O36**	**14.25 – 14:35**	Gioia Bongiorno	*Leishmania* and *Phlebovirus* detection in sand flies and canine sera samples
**O37**	**14.35 – 14:45**	Jovana Sadlova	Vector competence of European sand flies to *Leishmania* and phleboviruses
**O38**	**14.45 – 14:55**	Orin Courtenay	Development of a sand fly semio-chemical attractant remote monitoring device

**O39**	**14.55 – 15:05**	Iva Kolarova	Development of recombinant salivary antigens as risk markers
**15.05 – 15:10**		DISCUSSION	
**O40**	**15.10 – 15:20**	Yoni Waitz	Large-scale analysis of long-term historical data of sand fly populations
**O41**	**15.20 – 15:30**	Vladan Gligorijević	Originality, simplicity and reusability of visual tools developed for CLIMOS EWS
**O42**	**15:30 – 15:40**	Sergio Natal	The development of an Early Warning System under the framework of CLIMOS project
**O43**	**15.40 – 15:50**	Diana Guardado	Promoting and disseminating sand fly research to various audiences
**15.50 – 16:00**		DISCUSSION	
**16:00 – 16:30**		**COFFEE BREAK**	
**16:30 – 18:00**		**Leishmania Vector Interactions**<br>Chair: Petr Volf	
**O44**	**16.30 – 16:45**	Tiago Serafim	IgM promotes genetic exchange of *Leishmania* inside the sand fly vector
**O45**	**16.45 – 17:00**	Rodrigo Pedro Pinto Soares	Salivary glands of *Nyssomyia neivai* during *in vivo* infection by different Amazonian *Leishmania* (*Viannia*) species
**O46**	**17.00 – 17:15**	Cecilia Stahl Vieira	Azadirachtin disrupts ecdysone signaling and alters *P. perniciosus* immunity
**O47**	**17.15 – 17:30**	Erich Telleria	Surface molecules from *Leishmania* and bacteria increase the expression of sand fly genes coding for antimicrobial peptides and gut surface proteins
**O48**	**17:30 – 17:45**	Yara Traub-Cseko	Uncovering the secrets of vector competence focusing on two Amazonian sand fly populations from leishmaniasis endemic and non-endemic areas
**O49**	**17:45 – 18:00**	Barbora Vojtková	Infectiousness of natural hosts of *Leishmania major* to sand flies at micro- and macro-scale
		**18:45 BUS transfer to the restaurant**	
**19:30**		**GALA DINNER**	

13 FRIDAY

8:30 – 10:30			**Sand fly taxonomy, distribution, surveillance 1**<br>Chairs: Padet Siriyasatien /Jerome Depaquit
**O50**	8:30 – 8:45	Jerome Depaquit	Thoughts about the very complicated taxonomy of South-East Asian sandflies
**O51**	8:45 – 9:00	Huicong Ding	Hidden in plain sight: discovery of phlebotomine sandflies in Singapore and description of four species new to science
**O52**	9:00 – 9:15	Thanapat Pataradool	Suspected novel species of Phlebotomine sand flies (Diptera: Psychodidae) from Uthai Thani province, Thailand, and its potential role in *Trypanosoma* sp. transmission
**O53**	9:15 – 9:30	Khamsing Vongphayloth	Analysis of phlebotomine sandflies (Diptera: Psychodidae) in Laos from 2012-2024 identifies new species, and diverse taxa
**O54**	9:30 – 9:45	Stavroula (Evi) Gouzelou	Sand fly fauna investigation in refugee camps and emerging *L. tropica* foci in Cyprus
**O55**	9:45 – 10:00	Alessandro Alvaro	Investigations on the presence of sand fly species (Diptera: Psychodidae) in northern Italy and on the prevalence of *Leishmania* spp. in sand flies and reptile hosts
**O56**	10:00 – 10:15	Ina Hoxha	Sand flies (Diptera: Phlebotominae) in the Republic of Kosovo: distribution, ecology and pathogen circulation
**O57**	10:15 – 10:30	Fátima Amaro	National Surveillance Network for sandflies in Portugal: the importance of monitoring a less known vector

10:30 – 11:00		**COFFEE BREAK**	
11:00 – 12:30		**Sand fly taxonomy, distribution, surveillance 2**<br>Chair: Vit Dvorak	
**O58**	11:00 – 11:15	Eduardo Berriatua	Assessing Sand Fly Vector Distributions in Spain to Predict *Leishmania* and *Phlebovirus* Infection Risks: Insights from the CLIMOS Project
**O59**	11:15 – 11:30	Jamila Ghrab	*Sergentomyia ssp* in the Tunisian *Leishmania major focus* of Sidi Bouzid: Diversity and *Leishmania* infection
**O60**	11:30 – 11:45	Kamal Eddine Benallal	Sand fly fauna in leishmaniasis foci in Algeria: Species composition and blood meal sources studied by complementary techniques
**O61**	11:45 – 12:00	Petr Halada	MALDI-TOF MS protein profiling: A promising tool for identification of sibling species within the *Phlebotomus perniciosus/ longicuspis* complex
**O62**	12:00 – 12:15	Ognyan Mikov	First report of a cavernicolous population of *Phlebotomus neglectus* (Diptera: Psychodidae) breeding at suboptimal temperatures in a karst tourist cave in Bulgaria
**O63**	12:15 – 12:30	Tarcísio Milagres	Flebocollect project: citizen science as a tool to enhance sand fly surveillance and community engagement in Spain
12:30 – 14:00		**LUNCH (News Café Bernardin)**	
14:00 – 15:30		**Sand fly taxonomy, distribution, surveillance 3**<br>Chair: Yusuf Ozbel	
**O64**	14:00 – 14:15	Bruno Rodrigues	Hidden diversity in Monticola Series (Diptera, Psychodidae, Phlebotominae)
**O65**	14:15 – 14:30	Katharina Platzgummer	Sand fly research in Central Europe – the past, the present and the future
**O66**	14:30 – 14:45	Jorian Prudhomme	Phlebotomine sandflies distribution and abundance in France: a systematic review

**O67**	**14:45 – 15:00**	Ilaria Bernardini	*Phlebotomus perfiliewi* as incriminated vector species in endemic area of Tuscany region (Central Italy)
**15:30 – 16:00**		FAREWELL CLOSING	

ORAL PRESENTATIONS

O1 *In vitro* and *in vivo* experimental model of atypical cutaneous leishmaniasis by *Leishmania infantum* using salivary gland homogenate of *Lutzomyia longipalpis*

Flores Gabriela[1,2,3], Tomokane Thaise[1], Ovallos Fredy[4], Passero Felipe[2,3], Laurenti Márcia[1]*

[1]*Laboratório de Patologia das Moléstias Infecciosas, Faculdade de Medicina, Universidade de São Paulo, São Paulo, SP, Brasil*
[2]*Departamento de Ciências Biológicas e Ambientais, Instituto de Biociências, Campus do Litoral, Universidade Estadual Paulista, São Vicente, SP, Brasil*
[3]*Instituto de Estudos Avançados do Mar, Universidade Estadual Paulista, São Vicente, SP, Brasil*
[4]*Departamento de Epidemiologia, Faculdade de Saúde Pública, Universidade de São Paulo, São Paulo, SP, Brasil*

***Corresponding author:** mdlauren@usp.br

Abstract

In Central America, *Leishmania infantum* causes non-ulcerated cutaneous leishmaniasis (NUCL) beside to visceral leishmaniasis in the same geographical area. The skin lesion is characterized by small papule which do not ulcer with the time of infection; and large amount of CD8 T-lymphocytes IFN-γ^+ followed by M1-macrophages with scarce parasites is observed showing a pro-inflammatory response. However, little is known about the immunopathogenesis of this atypical clinical presentation. So, to deepen knowledge about this unusual parasite-host relationship, the present objective was to characterize an *in vitro* and *in vivo* experimental model using *Leishmania infantum* promastigotes isolated form NUCL plus *Lutzomyia longipalpis* salivary gland homogenate (SGH) for macrophage and BALB/c mice infection. BALB/c peritoneal macrophages were infected with 10 promastigotes per 1 macrophage add to SGH; and after 48 hours, the infection index was determined. BALB/c were infected intradermal with 10^6 promastigotes into right ear in the presence of SGH. Animals were euthanized at 20th and 40th days PI, fragments of skin and viscera were collected for histopathological studies and determination of parasite load. The presence of SGH increases the macrophages infection index; however, BALB/c mice did not show ear swelling and viable parasites in the skin, spleen and liver at 20th and 40th days PI, in the presence of SGH. Discreet mononuclear inflammatory infiltrate was observed in the dermis, and it was focal in the absence and diffuse in the presence of SGH. The results showed that *Lutzomyia longipalpis* SGH exacerbated the *in vitro* infection; however, *in vivo* infection was not evident even in the presence of *Lutzomyia longipalpis* SGH. Supported by FAPESP #2014/50315-0, #2022/15834-3, CNPq and LIM50 HC-FMUSP

Keywords: Atypical cutaneous leishmaniasis, *Leishmania (L.) infantum*, *Lutzomyia longipalpis*, experimental model

O2 The effect of *Phlebotomus duboscqi* saliva on the ongoing *Leishmania major* infection in a murine model

Jelinkova Kristyna[1]*, Dvorakova Barbora[1], Brezina Jiri[1], Pacakova Lenka[1], Becvar Tomas[1], Vojtkova Barbora[1], Ruzickova Katerina[1], Bardunek Valigurova Andrea[2], Kolarova Iva[1], Volf Petr[1]

[1]*Faculty of Science, Charles University, Prague, Czech Republic*
[2]*Faculty of Science, Masaryk University, Brno, Czech Republic*

***Corresponding author**: kristyna.jelinkova@natur.cuni.cz

Abstract

The timing and frequency of exposure to sand fly saliva can affect the host immune response, leading to different outcomes of *Leishmania* infection. This study aims to investigate the effect of various exposure schemes of BALB/c mice to *Phlebotomus duboscqi* prior and/or during *Leishmania major* infection, with specific focus on the conditions affecting the induction and maintenance of protective effect of sand fly saliva. Exposed mice were intradermally infected with *L. major* obtained from the midguts of infected sand flies. After 11 weeks of infection, mice were examined for disease outcome characterised by (i) lesion size and (ii) *L. major* parasites load in the tissues by qPCR and further subjected to a thorough immunological analysis by (iii) determination of the cellular immune response including myeloid and lymphoid cell populations by flow cytometry and immunohistochemistry and (iv) levels of antigen-specific antibodies against *L. major* and P*. duboscqi* by ELISA test. The preliminary results showed that continuous exposure to sand fly saliva after the infection does not diminish the protective effect induced by exposure prior to the infection, evaluated by the lesion size and parasite load in the infected ear and the corresponding lymph node. Moreover, an immunological analysis of the microenvironment of infected tissue showed the effect of sand fly saliva on myeloid cells populations such as eosinophils, macrophages, and monocytes, correlating also with the lesion size and its phenotype.

Keywords: sand fly saliva, *Phlebotomus duboscqi*, *Leishmania major*, immune response

Funding: The study was funded by Grant Agency of Charles University (GAUK), project no. 33622.

O3 Experimental transmission of *Mundinia* by biting midges and sand flies

Becvar Tomas[1]*, Vojtkova Barbora[1], Siriyasatien Padet[2], Votypka Jan[1], Modry David[3,4,5], Jahn Petr[6], Bates Paul[7], England Marion[8], Volf Petr[1], Sadlova Jovana[1]

[1]Department of Parasitology, Faculty of Science, Charles University, Prague, Czech Republic
[2]Vector Biology and Vector Borne Disease Research Unit, Department of Parasitology, Faculty of Medicine, Chulalongkorn University, Bangkok, Thailand
[3]Biology Centre, Institute of Parasitology, Czech Academy of Sciences, České Budějovice, Czech Republic
[4]Department of Botany and Zoology, Faculty of Science, Masaryk University, Brno, Czech Republic
[5]Department of Veterinary Sciences/CINeZ, Faculty of Agrobiology, Food and Natural Resources, Czech University of Life Sciences Prague, Prague, Czech Republic
[6]Equine Clinic, University of Veterinary Sciences, Brno, Czech Republic
[7]Division of Biomedical and Life Sciences, Faculty of Health and Medicine, Lancaster University, Lancaster, United Kingdom
[8]Entomology Group, The Pirbright Institute, Pirbright, Surrey, United Kingdom

***Corresponding author:** becvart@natur.cuni.cz

Abstract

The *Leishmania* subgenus *Mundinia* was established in 2016 and includes six species distributed on all continents, except of Antarctica. While the natural vectors of the other *Leishmania* are exclusively sand flies (Diptera: Psychodidae), the vectors of *Mundinia* remain uncertain; however, observations over the past decade have raised the possible involvement of biting midges (Diptera: Ceratopogonidae). The three sand fly species (*Phlebotomus argentipes*, *P. duboscqi*, and *Lutzomyia migonei*) and the biting midge *Culicoides sonorensis* were infected through a chicken skin membrane and dissected by day 3, 6, 10 post bloodmeal (PBM). Infected insects were also allowed to feed on the ear pinnae of anaesthetized BALB/c mice and the presence of parasite DNA in host tissues was immediately assessed by PCR. Our study demonstrated that *C. sonorensis* supports the development of all five *Mundinia* species tested, including those infecting humans, *L. martiniquensis*, *L. orientalis,* and *L. chancei,* which were subsequently transmitted to the naïve host by bite. On the other hand, sand flies with the same geographical distribution as the tested *Mundinia* did not transmit parasites to mammals, and only *L. martiniquensis* and *L. orientalis* developed late-stage infections in *P. argentipes*. These data support the hypothesis of the involvement of biting midges in the circulation of *Mundinia*, but do not exclude the involvement of sand flies. If confirmed by field studies, these findings may ultimately lead to a redefinition of the genus *Leishmania*, where sand flies are dogmatically considered to be the sole vectors.

Keywords: *Leishmania*, *Mundinia*, biting midges, sand flies

O4 Application and optimization of a *Lutzomyia longipalpis* transmission model to initiate various forms of cutaneous leishmaniasis in laboratory animals

Hendrickx Sarah[1], Caljon Guy[1]

[1]*Laboratory of Microbiology, Parasitology and Hygiene, University of Antwerp (LMPH UA), Belgium*

***Coresponding author:** sarah.hendrickx@uantwerpen.be

Abstract

Current *in vivo* animal models for cutaneous leishmaniasis often use artificial inoculation of high parasite numbers in the dermis to start infection. However, over the last decades, researchers have shown that artificial needle-initiated infections differ immunologically from natural parasite infections with phlebotomines and are therefore less suited to study vaccine efficacy for example. To include the immunological features of natural infection in our experimental animal models for drug discovery and vaccine evaluation, *Lu. longipalpis* (*LuLo*) was used to develop and optimize transmission models for *Leishmania major, L. tropica* and *L. braziliensis.* Strains of these parasite species were transfected with the bioluminescent *PpyRE9* and the fluorescent *DsRed* reporter genes to allow a semi-quantitative follow-up of parasite burdens upon transmission in parallel with the assessment of disease pathology. Given the different developmental patterns of the various parasite strains in the sand fly, a range of experimental approaches have been explored to increase establishment and transmissibility in *LuLo*. In addition, the exposure of rodents to various quantities of *LuLo*, at different time points after sand fly infection and at several dermal sites, revealed site- and time-specific differences in infection onset and disease severity. Our laboratory already successfully established a transmission model for *L. major*, and ongoing research is aiming to further optimize *L. tropica* and *L. braziliensis* models.

Keywords: cutaneous leishmaniasis; transmission; rodent infection models

O5 Mechanisms of *Leishmania* attachment to the sand fly midgut and the stomodeal valve

Pružinová Katerina[1], Yanase Ryuji[2,3], Moreira-Leite Flávia[2], Owino Barrack[2], Sádlová Jovana[1], Sunter D. Jack[2], Volf Petr[1]*

[1]*Department of Parasitology, Faculty of Science, Charles University, Prague, Czech Republic*
[2]*Department of Biological and Medical Sciences, Oxford Brookes University, Oxford, United Kingdom*
[3]*Department of Genetics and Genome Biology, College of Life Sciences, University of Leicester, Leicester, United Kingdom*

***Corresponding author**: volf@cesnet.cz

Abstract

The critical steps *Leishmania* life cycle within the vector include two different types of attachment, each facilitated by specific molecules. Nectomonads and leptomonads, parasite forms found in the middle phase of development in the sand fly, bind to the midgut epithelium by inserting their flagella between microvilli. In *P. papatasi*, this attachment is controlled by galectin, serving as a receptor for the terminal galactose on *L. major* lipophosphoglycan (LPG). In contrast, in permissive sand flies the attachment does not require LPG and is mediated by sand fly O-linked glycoproteins; in *Lutzomyia longipalpis* this molecule was characterized as a novel mucin. Specialized haptomonad forms attach to the cuticular lining of the stomodeal valve and destroy the valve, enhancing the parasite transmission by regurgitation. The flagellum of haptomonads is modified to mediate this attachment and is characterized by an attachment plaque which contains distinct structural elements. This strong and stable attachment to the insect vector is conserved across kinetoplastid parasites and is mediated by a specific set of proteins called KIAPs (kinetoplastid-insect adhesion proteins) which are localised in the attached haptomonad flagellum. In *Leishmania mexicana*, deletion of these KIAPs impaired adhesion *in vitro* and prevented parasites from colonising the stomodeal valve in *Lutzomyia longipalpis*, without affecting parasite growth and development in the sand fly midgut. Remarkably, deletion of the KIAPs caused a reduction in the amount of promastigote secretory gel, with concomitant reduction in midgut swelling. We expect that these KIAP proteins have an important function on disease transmission across the kinetoplastid parasites.

Keywords: *Phlebotomus*, *Lutzomyia*, *Leishmania*, midgut, stomodeal valve, host-parasite interactions

O6 *Leishmania* transmission is disrupted in sandflies colonized by *Delftia tsuruhatensis* TC1 bacteria

Cecilio Pedro[1]*, Rogerio Luana A.[2], Serafim Tiago D.[2], Tang Kristina[2], Willen Laura[2], Iniguez Eva[2], Meneses Claudio[2], Chaves Luis F.[3], Zhang Yue[4], Huang Wei[5], Castañeda-Casado Pablo[6], Jacobs-Lorena Marcelo[5], Valenzuela Jesus G.[2], Rodrigues Janneth[6]*, Oliveira Fabiano[2,7]*

[1]*Vector Biology Section, Laboratory of Malaria and Vector Research, National Institute of Allergy and Infectious Diseases, National Institutes of Health; Rockville, MD, 20852, USA*
[2]*Vector Molecular Biology Section, Laboratory of Malaria and Vector Research, National; Institute of Allergy and Infectious Diseases, National Institutes of Health; Rockville, MD, 20852, USA*
[3]*Department of Environmental and Occupational Health, School of Public Health-Bloomington, Indiana University; Bloomington, IN, 47405, USA*
[4]*Integrated Data Sciences Section (IDSS), Research Technologies Branch, National Institute of Allergy and Infectious Diseases, National Institutes of Health; Rockville, MD, 20892, USA*
[5]*Department of Molecular Microbiology and Immunology, Malaria Research Institute, Johns Hopkins Bloomberg School of Public Health; Baltimore, MD, 21205, USA*
[6]*Global Health Medicines R&D, GSK; Tres Cantos, Madrid, 28760, Spain*

***Corresponding authors:** pedro.amadocecilio@nih.gov, janneth-fatima-indira.x.rodrigues@gsk.com, loliveira@niaid.nih.gov

Abstract

Most human pathogenic *Leishmania* species are zoonotic agents; therefore, sandfly-based control strategies are essential to prevent parasite circulation. Here, we used a *Delftia tsuruhatensis* strain that inhibits the development of *Plasmodium* in mosquitoes, but in the context of *Leishmania*-infected sandflies. Using GFP-expressing *D. tsuruhatensis* TC1, we show that this bacterium colonizes the midgut of *Phlebotomus duboscqi* sandflies. Such colonization impacts the development of *L. major* parasites in the vector, as per the significantly lower number of both total and infectious metacyclic parasites detected in the midguts of bacteria-fed *versus* control sandflies (90% reduction). This phenotype was consistently observed, regardless of the timing of bacterial feeding (from 1 week prior to infection to 8 days after infection), and was even stronger in sandflies given a second, uninfected, bloodmeal. Curiously, our data suggest this phenotype is likely an indirect effect of TC1 colonization, related with the induction of sandfly gut dysbiosis. These results have biological significance, since we observed that *Leishmania*-infected, bacteria-fed sandflies are less able to transmit *Leishmania major* parasites and cause disease in a mouse model of cutaneous leishmaniasis (parasites detected in 27% of animals bitten by bacteria-fed flies *versus* 100% of animals in the control group). Relevantly, modelling studies based on our results support the disruption of disease endemicity in the field. Altogether, these results highlight TC1 as a promising vector-based approach for the control of leishmaniasis in the field.

Keywords: *Leishmania*, sandfly, vector refractoriness, *Delftia tsuruhatensis*, gut dysbiosis

O7 Contribution of real-time PCR targeting kinetoplast DNA to access sandfly infection rate in areas with low *Leishmania infantum* transmission

Bouratbine Aïda[1]*, Weslati Marwa[1], Benabid Meriem[1], Ghrab Jamila[1,2], Souissi Olfa[1], Aoun Karim[1]

[1]*Lab of Medical Parasitology, Biotechnology and Biomolecules LR20-IPT-06, Institut Pasteur of Tunis, Manar University, Tunisia*
[2]*High Institute of Sciences and Technology of Environnement, Borj-Cedria, Carthage University, Tunisia*

***Corresponding author**: aida.bouratbine@pasteur.rns.tn

Abstract

Real-time PCR (qPCR) targeting minicircle kinetoplast DNA (kDNA) is a highly sensitive method that allows *Leishmania* identification. It could detect one flagellate per dissected sandfly gut which legitimates its use as a screening method for the study of sandfly infection. Furthermore, kDNA qPCR can determine parasite load, a high burden being correlated with strong evidence of *Leishmania* transmission.
The objective of this work was to access the usefulness of kDNA qPCR in screening *Leishmania* infection and determining *L. infantum* vectors in an area with low *Leishmania (L.) infantum* transmission.
A sample of 878 females was collected from a focus of low incidence of visceral leishmaniasis. DNA was extracted separately from each female individual and subjected to kDNA qPCR using a TaqMan probe. *Leishmania* infection was first screened by pools of 5 sandflies' DNA. Then, DNAs from sandflies grouped into each positive pool were analyzed individually to determine the positive specimens and to quantify the parasite load of each specimen. All positive kDNA qPCR specimens were systematically amplified by ITS1-PCR and *Leishmania* species were determined by ITS1 sequencing. Sandfly species were determined by barcoding.
Real-time PCR identified four positive specimens (0.45%). ITS1-PCR sequencing allowed *L. infantum* identification in only one kDNA qPCR-positive specimen out of 4. This was a *P. perniciosus* female with thousands of parasites. For the other 3 specimens, all were *P. perniciosus* harboring less than 20 parasites for which *Leishmania* species identification was not possible using the ITS1 target.
This work highlights the usefulness of kDNA qPCR in screening *Leishmania* infection.

Keywords: Real-time PCR, kinetoplast DNA, *Leishmania*, *L. infantum*, sandfly

O8 Are bites of non-infected sand flies important for the maintenance of cutaneous leishmaniasis in animal reservoirs?

Cecilio Pedro[1], Tang Kristina[2], Felix Luiza dos Santos[2], Disotuar Maria M.[2], Meneses Claudio[2], Serafim Tiago D.[1], Valenzuela Jesus G.[2], Oliveira Fabiano[1]*

[1]*Vector Biology Section, Laboratory of Malaria and Vector Research, National Institute of Allergy and Infectious Diseases, National Institutes of Health, Rockville, MD, USA*
[2]*Vector Molecular Biology Section, Laboratory of Malaria and Vector Research, National Institute of Allergy and Infectious Diseases, National Institutes of Health, Rockville, MD, USA*

***Corresponding author**: loliveira@nih.gov

Abstract

Sandflies transmit a variety of pathogens, of which the *Leishmania* parasites are the most important. More than twenty species of *Leishmania* cause disease in humans on at least five continents. It is striking that all but one of these parasite species are zoonotic pathogens. Therefore, animal reservoirs play an important epidemiological role in leishmaniasis. Since rodents in the wild are potential reservoirs for *Leishmania major* parasites, we used two rodent models of cutaneous leishmaniasis to investigate whether the bites of uninfected *Phlebotomus duboscqi* sandflies influence the course of cutaneous leishmaniasis. When uninfected sandflies were allowed to feed once on active lesions of cutaneous leishmaniasis, this had no effect on disease progression, and the parasite loads in the mice ears were similar to control animals (not exposed to sandfly bites) throughout the follow-up period. However, when uninfected sand flies were allowed to bite healed cutaneous leishmaniasis ear lesions, a transient pathological response was observed, as evidenced by an increase in ear thickness in the exposed animals compared to the control animals. Importantly, this phenotype was accompanied by a significant increase in parasite load in the ear of the exposed animals compared to control animals. Remarkably, greater pathological changes were observed when healed cutaneous leishmaniasis lesions were repeatedly exposed to the bites of uninfected *P. duboscqi* sandflies. These preliminary results suggest that uninfected sandflies may play an important role in maintaining competent *Leishmania* reservoirs.

Keywords: sandfly, *Leishmania*, saliva

O9 *Leishmania* hybridization in their sand fly vectors: investigation of the role of Gex1

Fuentes-Carias Marcela[1]*, Späth Gerald F.[1], Louradour Isabelle[1]

[1]*Institut Pasteur, Université Paris Cité, Unité de Parasitologie moléculaire et Signalisation, Paris, France*

***Corresponding author:** marcela.fuentes-carias@pasteur.fr

Abstract

Leishmania parasites can produce hybrid genotypes through a cryptic sexual reproductive cycle which occurs in the gut of their sand fly vectors. *Leishmania* experimental hybridization can be achieved *in vivo* (by performing artificial sand fly coinfection) or *in vitro*. The frequency of hybrid formation is higher *in vivo* than *in vitro*, indicating that the conditions within the sand fly's gut are crucial to promote hybridization. Our research focuses on the study of Gex1, a protein recognized in other organisms for its role in nuclear fusion, via an approach combining genome editing, sand fly infections and the generation and use of specific polyclonal antibodies.

Using CRISPR/Cas9, we engineered *Leishmania* Gex1 *null* mutant and addback strains. While the wild type and addback controls successfully produce *in vitro* hybrids, Gex1 mutants failed to generate any, showing that Gex1 function is required for *Leishmania* sexual reproduction *in vitro*. In asymmetrical crosses - where only one parent expresses Gex1 - hybrid frequency strongly decreases but is not abolished, indicating that Gex1 is required in only one parental cell. We are currently characterizing the phenotype of these parasites and performing *in vivo* assays to test their mating competence in the sand fly. Additionally, we generated antibodies targeting different domains of Gex1, with the aim to track the protein expression and to identify key interaction partners.

Altogether, we demonstrated that Gex1 is essential in *Leishmania* hybridization and developed tools that will allow us to elucidate its mechanism of action *in vitro* and in the sand fly's gut.

Keywords: Vector/parasite interaction, *Leishmania*, hybrids, CRISPR/Cas9

O10 New evidence indicating the endemic transmission of *Leishmania donovani* in Israel

Studentsky Liora[12]*, Orshan Laor[1], Akad Fouad[3], Ben-Avi Irina[1], Diaz Debora[1], Elbaz Shirly[1], Sagi Orly[4], Davidovich-Cohen Maya[1], Kirstein Oscar David[1], Baneth Gad[2]

[1]*Ministry of Health, Central Laboratories for Public Health, Jerusalem, Israel*
[2]*The Hebrew University of Jerusalem, Rehovot, Israel*
[3]*Ministry of Health, National Laboratory for Public Health, Tel Aviv, Israel*
[4]*Soroka University Medical Center, Beer-Sheba, Israel*

***Corresponding author:** liora.studenetsky@moh.gov.il

Abstract

Three different cycles of leishmaniasis transmission have been documented in Israel: Cutaneous leishmaniasis, caused by *Leishmania major* or *Leishmania tropica*, and visceral leishmaniasis, caused by *Leishmania infantum*. This study examines the existence of a fourth cycle involving *Leishmania donovani*, the causative agent of visceral leishmaniasis, and the possible involvement of sand fly species and reservoirs. Sand flies were collected in the southern Negev desert using CDC traps. Female sand flies were analyzed for *Leishmania* DNA using specific gene targets. Molecular analysis differentiated sand fly species and identified blood meal sources. Real-time PCR and high-resolution melting (HRM) confirmed sand fly species, blood meal source, and *Leishmania* infection. Out of 22,636 collected sand flies, *Phlebotomus alexandri* comprised 80%. Analysis of female sand flies (n=5,019) revealed a 2.5% infection rate with *Leishmania* DNA, with 92% identified as *L. donovani*. Phylogenetic analysis confirmed distinct *Leishmania donovani* and *Leishmania infantum* clusters. Female engorge *Phlebotomus* alexandri sand fly positives for L. donovani had blood meals from European hares (*Lepus europaeus*). Additionally, *Leishmania* DNA from a human cutaneous leishmaniasis case in southern Israel matched the *Leishmania donovani* found in *Phlebotomus alexandri*.
This study reveals the presence of *Leishmania donovani* infection in the Negev desert, suggesting the potential establishment of a fourth leishmaniasis transmission cycle in Israel. The association with cutaneous lesions in humans, the identification of *Phlebotomus alexandri* as a putative vector, and the presence of hares as potential reservoirs highlight the need for further investigation. This finding has significant public health implications.

Keywords: cutaneous leishmaniasis, *Leishmania donovani*, *Leishmania infantum*, *Phlebotomus alexandri*, zoonoses

O11 The first detection of *Leishmania donovani* DNA in Türkiye, in its proven vector *Phlebotomus alexandri*

Arserim Suha Kenan[1]*, Özbilgin Ahmet[2], Limoncu M. Emin[1], Omondi Zeph N.[3], Demir Samiye[4], Çavuş Ibrahim[2], Töz Seray[5], Özbel Yusuf[5], Balcioğlu I. Cüneyt[2]

[1]*Vocational School of Health Services, Manisa Celal Bayar University, Manisa, Türkiye*
[2]*Department of Parasitology, Faculty of Medicine, Manisa Celal Bayar University, Manisa, Türkiye*
[3]*Department of Biology, Graduate School of Natural and Applied Sciences, Ege University, İzmir, Türkiye*
[4]*Department of Biology, Faculty of Science, Ege University, İzmir, Türkiye*
[5]*Department of Parasitology, Faculty of Medicine, Ege University, İzmir, Türkiye*

***Corresponding author:** suhaarserim@hotmail.com

Abstract

Sand flies serve as vectors for the *Leishmania* parasites in Türkiye. In this study, it was aimed to identify sand fly species present in endemic areas in Manisa province in the West part of country and investigate the presence of *Leishmania* parasites in sand flies.
Sand fly samples were collected in leishmaniasis endemic areas in Manisa and identified morphologically. Using the real-time ITS1 PCR, the presence of *Leishmania* parasite was detected and the melting point was used to differentiate *Leishmania* species. Additionally, to distinguish *L. donovani* and *L. infantum*, the cysteine protease B (*cpb*) gene region was amplified and visualized under UV light by performing 3% agarose gel electrophoresis with SYBR Green nucleic acid dye.
A total of 2050 sand fly samples were collected. Six sand fly species of the genus *Phlebotomus* (*P. papatasi, P. major s.l., P. tobbi, P. alexandri, P. sergenti s.l.* and *P. simici*) and two species of the genus *Sergentomyia* (*S. minuta* and *S. dentata*) were detected. A total of 178 pools were prepared from the female samples obtained, and *Leishmania* positivity was detected in 12 pools. Five of them (three *P. tobbi*, one *P. major* s.l. and one *P. alexandri*) were found positive for *L. donovani.*
Leishmania donovani DNA was detected for the first time in Türkiye, in its proven vector *P. alexandri.* These results will contribute to the assessment of potential risks for the management of vector-borne diseases in Turkey.

Keywords: Sand fly, *Phlebotomus alexandri*, *Leishmania donovani*, Türkiye

This study was supported by the Manisa Celal Bayar University Scientific Research Projects Coordination Unit. Project Number: 2018-117.

O_{12} Neotropical sand fly anthropophily – a natural or unnatural behaviour

Shaw Jeffrey*

Parasitology Department, São Paulo University, São Paulo, Brazil

***Corresponding Author** jayusp@hotmail.com

Abstract

One the major factors in evaluating a sand fly's epidemiological importance is determining if it attracted is to man. Early studies used human bait. Due to ethical considerations this was abandoned and replaced with Shannon trap catches. Within today's ethical network identifying blood meals is considered as the best option for establishing feeding habits that can be used to assess anthropophily. There are weaknesses with all these methods and anthropophily is not an evolutionary selected behaviour. So, can basic behaviour be used to predict and assess anthropophily? Accumulated information on neotropical sand flies suggests it can. Leishmaniinae Infections, trapping methods, population density and blood meal analyses indicate that species can be broadly grouped as "Generalists" that feed on a wide range of hosts or "Specialists" that do not. Within this grouping anthropophilic species are normally generalists. Epidemiological importance also relates to adaptation to anthropogenic environments. There are generalists that have specific environmental preferences that do not adapt to peri-domestic situations. Many generalist species of *Psychodopygus* and *Lutzomyia* are anthropophilic. Species of the former genera primarily occur in forests and do not adapt to anthropogenic environments. However, species of the second genus occur in many natural biomes and do adapt to peri-domestic situations. Under exceptional conditions a non-anthropophilic species may bite man and transmit a parasite. Such a species can be considered as an anthropportunist.

Keywords: anthropophily, anthropogenic, Generalist, Specialist, feeding habits, vector

Financial support: FAPESP grants 2012/05388-4; 2022/08550-9.

O13 Electrophysiological and behavioural responses of *Phlebotomus perniciosus* to volatile organic compounds of dogs and humans

Bezerra Santos Marcos Antonio[1]*, Zeni Valeria[2], Pistillo O. Marco[3], Bedini Stefano[2], Benelli Giovanni[2], Germinara G. Salvatore[3], Volf Petr[4], Otranto Domenico[1, 5]

[1]*Department of Veterinary Medicine, University of Bari, Italy*
[2]*Department of Agriculture, Food and Environment, University of Pisa, Italy*
[3]*Dipartimento di Scienze Agrarie, Alimenti, Risorse Naturali E Ingegneria, University of Foggia*
[4]*Department of Parasitology, Faculty of Science, Charles University, Czech Republic*
[5]*Department of Veterinary Clinical Sciences, City University of Hong Kong, China*

***Corresponding author:** marcos.bezerrasantos@uniba.it

Abstract

The olfactory response of phlebotomine sand flies is a key facet for studying their interactions with vertebrate hosts and associated vector-borne pathogens. Such studies are performed by assessing the electrophysiological and behavioural responses of these insects towards host-borne volatile organic compounds (VOCs). Nonetheless, scanty studies on this topic are available only for species of the subgenera *Lutzomyia* and *Nyssomyia* in South America, leaving a void for Old World sand fly species of the genus *Phlebotomus*. Herein we evaluated the olfactory responses of *Phlebotomus perniciosus*, one of the most important vectors of *L. infantum* in the Old World. To test the behavioural responses of sand flies to VOCs, 28 compounds isolated from humans and dogs were assessed using electrophysiological (i.e., electroantennogram, EAG) and behavioural assays (i.e., Y-tube olfactometer). In EAG trials, 14 compounds (i.e., acetic acid, nonanoic acid, 2-propanol, 2-butanol, pentanal, hexanal, nonanal, (*E*)-2-nonenal, decanal, myrcene, *p*-cymene, verbenone, 2-ethyl-1-hexanol, and acetonitrile) elicited significant antennal responses (i.e., ≥0.30 mV) in female sand flies. Six VOCs (i.e., acetic acid, 2-butanol, pentanal, hexanal, nonanal, (*E*)-2-nonenal) were selected for the behavioural assays. At the dose tested, nonanal was significantly attractive for *P. perniciosus* females, whereas the other compounds did not elicit any significant chemotaxis in sand flies. The attraction indexes varied from 0.53 for nonanal (most attractive) to -0.33 to pentanal (less attractive). Overall, our results contribute to understanding the role of olfactory cues routing host seeking behaviour in *P. perniciosus*, with implications to develop sustainable sand fly monitoring and management in leishmaniasis endemic areas.

Keywords: sand flies; leishmaniasis; VOCs; electrophysiology; olfactory cues

O14 The efficacy of a synthetic sex-aggregation pheromone to detect *Lutzomyia longipalpis*

Courtenay Orin[1]*, Gurgel-Gonçalves Rodrigo[2], Goto Hiro[3], Vianna Elisa[2], Hamilton James[4]*

[1]*Zeeman Institute and School of Life Sciences, University of Warwick, UK*
[2]*Laboratório de Parasitologia Médica e Biologia de Vetores, Universidade de* Brasília*, Brazil*
[3]*Instituto de Medicina Tropical de São Paulo, Universidade de São Paulo, Brazil*
[4]*Biomedical and Life Sciences, University of Lancaster, UK*

***Corresponding authors:** orin.courtenay@warwick.ac.uk, j.g.hamilton@lancaster.ac.uk

Abstract

Vector surveillance is an important element of mitigating vector-borne infections. CDC light traps are a convenient tool to monitor Phlebotomine sand fly vectors of *Leishmania* and *Phleboviruses*, however their accuracy in measurements of sand fly presence and/or relative abundance can be poor.

Here we review the potential deployments of a synthetic copy of the sex-aggregation pheromone produced by males of *Lutzomyia longipalpis*, the predominant sand fly vector of *L. infantum* causing visceral leishmaniasis in the Americas.

Cluster randomise trials have demonstrated that the synthetic pheromone deployed in controlled-release dispensers and co-located with insecticide, can reduce *L. infantum* infection incidence in the canine reservoir, and to reduce vector numbers in treated and non-treated nearest neighbour houses.

We also report results on the efficacy of the synthetic pheromone to attract the vector to CDC light traps field tested in widely distributed locations across 7 states in Brazil, where *Lu. longipalpis* is known to occur, and importantly in locations where it has not been previously recorded.

The recent results demonstrate the potential of the synthetic pheromone to be used for monitoring the geographical presence of *Lu. longipalpis*, which could form the basis of an early warning system (EWS) against pathogen transmission.

Keywords: *Lutzomyia longipalpis*, leishmaniasis, vector, pheromone, control, surveillance, Brazil

O_{15} Oviposition ecology of *Phlebotomus papatasi* sandflies: patterns, processes, and applications

Wasserberg Gideon[1]*, Kowacich-Swaney Dannielle[1], Ponnusamy Loganathan[2], Schal Coby[2]

[1]*Department of Biology, University of North Carolina at Greensboro, Greensboro, NC*
[2]*Department of Entomology and Plant Pathology, North Carolina State University, Raleigh, NC*

***Corresponding author:** g_wasser@uncg.edu

Abstract

A sustainable alternative to the delivery of an insecticide to the vector is to bring the vector to the insecticide using attractants. In this project, we applied a multi-disciplinary approach for bioassay-guided fractionation of semiochemicals from organic matter and conspecific origin for developing an optimal oviposition lure for the control and surveillance of *Phlebotomus papatasi* sand flies (a vector of Old-World cutaneous leishmaniasis [CL]). We identified larval conditioned rearing medium as a potent source for oviposition attractants and stimulants. We showed that this attraction was mediated by a sub-set of highly attractive bacterial strains that produce several volatile attractive compounds. We also screened various conspecific stages as a potential source of oviposition attractants. We found that conspecific eggs and first instar larvae were highly attractive and stimulated oviposition whereas older stages were not. Conspecific eggs had a dose dependent effect on sand fly behavior inducing attraction at low-intermediate doses and repellence at high doses. We found that this attraction pattern was mediated by dodecanoic acid as an egg and larval pheromone. We also identified Isovaleric acid as an important attractant produced by both young conspecific stages as well as by the most attractive bacterial isolate. In addition, we discovered that attraction to potential oviposition sites is mediated by visual cues with gravid females attracted to dark oviposition cups. Finally, we described the circadian rhythm of *P. papatasi* oviposition behavior. These findings are instrumental in guiding us through the effort of developing an attract-and-kill oviposition trap.

Keywords: Disease ecology, old world cutaneous leishmaniasis, oviposition attractants

O16 Effects of the Lunar Cycle on the Nocturnal Activity rhythm and Hourly Man-biting rates of *Phlebotomus orientalis*, the vector of visceral leishmaniasis in Sudan

Dakein Osman[1], Elnaiem Dia-Eldin[2]*

[1]*Kala azar Research Center, University of Gedarif, Gedarif state, Sudan*
[2]*Department of Natural Sciences, University of Maryland Eastern Shore, Maryland USA*

***Correspondence author:** daelnaiem@umes.edu

Abstract

Proper understanding and quantitation of sandfly nocturnal activity rhythm and hourly man-biting rates is an important requirement for designing effective control measures against leishmaniasis. Although several studies attempted to determine these parameters for different sandfly species and under different ecological conditions, little is known about the effect of the lunar cycle on the timing of sandfly activity and man-biting rates. This study was conducted in a visceral leishmaniasis endemic village (Helat-Belo), Gedarif state, Sudan, where the vector is *Phlebotomus orientalis*. Sandflies captured by 2 CDC light Traps and 3 human landing volunteers on an hourly basis, throughout a full lunar month (Ramadhan, 16 May – 12 June 2018), were identified and subjected to statistical analysis to determine the hourly nocturnal activity and man-biting rates of *P. orientalis* at different phases of the moon. The overall monthly average results showed that the human landing frequency of *P. orientalis* dropped from a median of 35 females at 6-8 pm to 1 female at 10-12 am and then peaked at 99 females at 2-4 am. Similarly, the light trap collections dropped from the median number of 84 females per trap night at 6-8 pm to 6 females per trap night at 10-12 am and then increased to 71 females per trap night at 2-4 am. The moon phase showed significant influence ($p<0.001$), not only on the total number of females per night but also on the specific timing of sandflies activity and man-biting rates. The results have important implications for the control of sandflies and leishmaniasis.

Keywords: sandflies, activity rhythm, lunar cycle

O17 Assessing *Wolbachia* circulation in field populations of *Lutzomyia longipalpis* in and endemic area of visceral leishmaniasis in Brazil

Andrade do Rosário Amanda[1]*, Posada-Lopez Laura[1], Rocha Marília Fonseca[2], Bandeira Maia Gledson[1], Werneck L. Guilherme[2], Galvis-Ovallos Fredy[3]

[1]*Postgraduate-program in Public Health, Faculty of Public Health, University of São Paulo, Brazil*
[2]*Zoonosis Control Center, Montes Claros – Minas Gerais, Brazil*
[3]*Departament of Epidemiology, Faculty of Public Health, University of São Paulo, Brazil*

***Corresponding author**: amandarandrade@yahoo.com

Abstract

Vector-borne diseases have a significant impact on global mortality and morbidity. While chemical control with insecticides is common for this purpose, resistance to these compounds limits their effectiveness. Thus, biological control emerges as a promising alternative, including the endosymbiotic bacterium *Wolbachia pipientis*, which infects 20 to 75% of insect species, including sand flies. *Wolbachia* influences insect development by causing reproductive alterations such as cytoplasmic incompatibility, male killing, feminization, and parthenogenesis. Due to these attributes, it has been suggested as a potential biological control tool. Accordingly, this study aimed to investigate the circulation of *Wolbachia* infection in *Lutzomyia longipalpis* populations in a visceral leishmaniasis (VL) endemic area in Brazil. Sand fly captures were carried out in Montes Claros, Minas Gerais, using light traps between August 2021 and July 2022. From the captured insects, a random sample of females was selected, identified morphologically, and subjected to individual DNA extraction. The 16S gene was used as a target for *Wolbachia* identification, and the surface protein gene (*wsp*) was used to determine the circulating *Wolbachia* strains. Of the 939 females analyzed, 21 tested positive for *Wolbachia* DNA, representing 2.2% of the sample, of which 14 samples were sequenced for strain identification. In this context, our results suggest a low prevalence of *Wolbachia* infection in the field population of *Lu. longipalpis*. This strain identification in *Leishmania* sp. vector populations and the evaluation of the bacterium's genetic diversity in sandflies may be the first step towards using this bacterium as a potential biological control agent.

Keywords: Wolbachia strains, biological control, Lutzomyia longipalpis, Brazil

O18 Dual infection of *Wolbachia* and *Candidatus* Tisiphia to *Sergentomyia squamirostris* in Japan

Kentaro Itokawa[1#], Kanako Minagawa[2#], Akihiro Kuroki[2], Daisuke Kobayashi[1], Haruhiko Isawa[1], Shinji Kasai[1], Kyoko Sawabe[1], Chizu Sanjoba[2]*

[1]*Department of Medical Entomology, National Institute of Infectious Diseases, Tokyo, Japan*
[2]*Graduate School of Agricultural and Life Sciences, The University of Tokyo, Japan*
[#]*Those authors were equally contributed*

***Corresponding author**: asanjoba@g.ecc.u-tokyo.ac.jp

Abstract

Maternally transmitted endosymbiotic bacteria exhibit various strategies to manipulate host reproduction or mutualism, ensuring their propagation and persistence within host populations. Understanding the diversity of symbiotic species present in populations of disease-transmitting vectors, as well as the impact of these infections on host ecologies, is crucial for devising effective control measures. The sand fly population in the main island of Japan constituted of a single species, *Sergentomyia squamirostris*. Although we have previously detected phlebovirus from wild population of this species, their vectoral status for human and animal diseases is not yet known. In our study, we conducted whole-genome sequencing and *de novo* assembly of the *S. squamirostris* genome using DNA isolated from individual female fly directly collected from filed. During this attempt, we unexpectedly obtained complete genomes of two bacteria, wSSQ and RiSSQ which were identified as *Wolbachia* and *Candidatus* Tisiphia (a.k.a "Torix *Rickettsia*"), respectively, among the assembled contigs. Sequencing reads of both wSSQ and RiSSQ genomes demonstrated high coverage compared to the depth of the host chromosomes, indicating their status as endosymbionts of *S. squamirostris*. Indeed, both bacteria were detected in both eggs and larvae of infected females, suggesting vertical transmission as the primary mode of infection for RiSSQ, similar to *Wolbachia*. The infection rates of wSSQ and RiSSQ varied across different localities in Japan, with co-infection by both bacteria being commonly observed. Notably, wSSQ was detected in both male and female *S. squamirostris*, while RiSSQ was detected only in female sand flies in all populations.

Keywords: *Wolbachia*, *Candidatus* Tisiphia, symbionts, *Sergentomyia squamirostris*

O_{19} Role of the microbioma in *Lutzomyia longipalpis* Vector competence: What do we know?

Campolina Thais[1], Pimenta Paulo[1], Secundino Nagila[1]*

[1]*Instituto René Rachou, Fundação Oswaldo Cruz, Brazil*

***Corresponding author:** secundinon@gmail.com

Abstract

The bacterial components of the microbiota can disrupt the growth of *Leishmania* inside the sand fly carrier. There is still a lack of comprehensive research on the diversity of microbiota and the interactions between host-microbiota-pathogen in wild New World sandflies, especially in the case of *Lutzomyia longipalpis*. The composition and number of microbiota differ depending on the source of food, life stages, and physiological factors. The presence of bacteria in the midgut of sandflies can have an impact on the development and survival of the parasite. We conducted a metagenomic analysis, cultivating and sequencing the 16s rRNA gene to determine the composition and abundance of the microbiota in *L. longipalpis* from Lapinha Cave in Brazil. By aggregating the taxonomic indices from all experimental circumstances, we identified a core composition consisting of Enterobacter, Serratia, Stenotrophomonas, Enhydrobacter, Pseudomonas, and Chryseobacterium. The culture-dependent 16s rRNA sequencing identified the presence of Bacillus, Enterococcus, Erwinia, Enterobacter, Escherichia, Klebsiella, Lysinibacillus, Pseudocitrobacter, Providencia, Pseudomonas, Serratia, Staphylococcus, and Solibacillus. We examined the impact of individual indigenous bacteria from *Lu. longipalpis* both in laboratory settings and in living organisms by co-cultivating them with promastigotes of *L. infantum chagasi*, *L. major*, *L. amazonensis*, and *L. braziliensis*. Following 24 hours of co-cultivation, a decrease in growth was noted in all species of parasites. Significant differences were seen between the groups co-infected with the bacterial species Lysinibacillus, Pseudocitrobacter, and Serratia in the in vivo co-infection of *L. infantum chagasi*, *L. major*, and *L. amazonensis*, as well as in their supernatants. Based on these findings, the core microbiota and the symbiotic bacteria (Lysinibacillus, Serratia, and Pseudocitrobacter) are potentially suitable for paratransgenesis. Funding was provided by the Conselho Nacional de Desenvolvimento Científico e Tecnológico (CNPq), INCT-EM, and Fiocruz.

Keywords: Sand flies, microbiota, *Leishmania*

O20 Phleboviruses and *Leishmania* detection in sandflies: three years of monitoring in Lombardy and Emilia-Romagna Regions (Northern Italy)

Calzolari Mattia[1]*, Defilippo Francesco[1], Carrera Maya[1], Grisendi Annalisa[1], Scremin Mara[1], Salvato Sara[1], Lelli Davide[1], Manarolla Giovanni[2], Taddei Roberta[1], Rugna Gianluca[1], Carra Elena[1], Dottori Michele[1], Puggioli Arianna[3], Colonna Roberta[3], Angelini Paola[4], Lavazza Antonio[1]

[1]*Istituto Zooprofilattico Sperimentale della Lombardia e dell'Emilia-Romagna "B. Ubertini", Brescia, Italy*
[2]*Welfare Department, Lombardy Region, Milan, Italy*
[3]*Centro Agricoltura Ambiente "G. Nicoli", Crevalcore, Italy*
[4]*Regional Health Authority of Emilia-Romagna, Bologna, Italy*

***Corresponding author:** mattia.calzolari@izsler.it

Abstract

Phlebotomine sandflies represent a group of small hematophagous insects belonging to the *Psychodidae* family. These dipterans are relevant for public health since they play a pivotal role in transmitting several microorganisms, including *Leishmania* parasites, the causative agents of the neglected disease leishmaniasis, and phleboviruses. This study was conducted to investigate and characterize the phleboviruses and *Leishmania* infections in vector sandflies collected in the Lombardy and Emilia-Romagna regions (Northern Italy). From 2021 to 2023, more than 250 sites in semi-natural and suburban environments around villages were sampled using modified CDC traps baited with CO_2, sampling 119,389 sandflies. The most collected species in Lombardy was *Phlebotomus perniciosus* (65%), followed by *Phlebotomus perfiliewi* (33%); in Emilia-Romagna, this last species was the most abundant (92%). We examined pools of 25-50 females using biomolecular methods to search for phleboviruses and *Leishmania* spp. Overall, we tested 2051 pools by using specific real-time PCRs, a pan-phlebovirus PCR, and MinON sequencing. Of these, *Leishmania* was detected in 294 pools, Toscana virus in 78. Moreover, other phleboviruses were detected as Fermo virus, Ponticelli virus, and Sicilia virus. The Leishmania characterization with MLMT identifies two different sub-population of parasites circulating in the surveyed area. The quality-quantitative analysis of sandflies was used to obtain information on the composition and density distribution of sandflies between seasonal periods and surveillance years in the surveyed area. These results may be useful for future monitoring and control programs aimed at reducing the risk of *Leishmania* and phleboviruses infections.

Keywords: *Phlebotomus perniciosus*, *Phlebotomus perfiliewi, Leishmania*, *Phlebovirus*

O21 Susceptibility of various sand fly species to Toscana virus

Polanska Nikola[1]*, Thiesson Adrien[2], Arnaud Frédérick[2], Stejskalova Marketa[1], Rehbergerova Marketa[1], Kohl Alain[3], Volf Petr[1], Ratinier Maxime[2], Jancarova Magdalena[1]

[1]*Laboratory of Vector Biology, Dpt. of Parasitology, Faculty of Science, Charles University, Prague, Czech Republic*
[2]*IVPC UMR754, INRAE, Universite Claude Bernard Lyon 1, EPHE, PSL Research University, F-69007 Lyon, France*
[3]*Departments of Tropical Disease Biology and Vector Biology, Liverpool School of Tropical Medicine, UK*

***Corresponding author**: nikola.polanska@natur.cuni.cz

Abstract

Phlebotomine sand flies transmit numerous viral pathogens, including Toscana virus (TOSV, *Phenuiviridae*). This arbovirus is spread in the Mediterranean area and causes a wide range of clinical symptoms (from non-symptomatic to serious CNS infection). While only *Phlebotomus pernicious* and *P. perfiliewi* have been confirmed as TOSV vectors, infected or TOSV-seropositive humans and animals have been identified in regions lacking these sand fly species. Given the limited understanding of TOSV and its spread, we aimed to assess the susceptibility of other sand fly species to TOSV, potentially expanding our knowledge of its transmission in nature. The susceptibility of *P. papatasi*, *P. tobbi*, *P. sergenti,* and *Sergentomyia schwetzi* to TOSV was tested by membrane feeding with blood mixed with TOSV strains belonging to the genetic lineage A or B (referred to as TOSV-A or TOSV-B). Blood-fed females were dissected at days 4, 8, and 14 post-infection for virus quantification using both infectious viral particle titration and RT-qPCR. We show that the TOSV-A did not infect any tested sand fly species. Contrarily, TOSV-B infected *P. tobbi* at relatively high rates (66% and 53% at D4 and D8, respectively). *Phlebotomus sergenti* showed lower infection rates (5.5%) but 100% dissemination rate. *Phlebotomus papatasi* and *S. schwetzi* were 100% refractory to TOSV-B. Overall, our data indicate that *P. tobbi* is highly susceptible to TOSV and potentially serves as the TOSV vector in the Eastern part of the Mediterranean basin, when *P. sergenti* is less susceptible, but its role in TOSV circulation should be also considered.

Keywords: Phlebotomine sand flies, Toscana virus, infection, vector, TOSV

O22 Detection of natural infection of *Leishmania donovani* among wild caught phlebotomine sandflies in India: an endemic focus of Leishmaniasis

Saini Prasanta[1]*, Shah Harish Kumar[1], Fathima PA[1]

[1]*Indian Council of Medical Research- Vector Control Research Centre, Field Station, Kerala, India*

***Corresponding author:** prasantasaini09@gmail.com

Abstract

Sandflies are the only proven vectors of leishmaniasis, a neglected tropical disease. *Leishmania donovani* is one of the *Leishmania* species that cause both visceral and cutaneous leishmaniasis (CL, VL) in the Indian subcontinent. The dual tropism of the parasite and the widespread distribution of *L. donovani* infection indicate the abundance of sandfly vectors. As part of the nationwide vector surveillance of L. donovani in India, sandflies were collected in five Indian zones (North, South, West, East and Central India) from 2021 to 2023 based on leishmaniasis case reports. Using CDC-modified light traps and mechanical aspirators, sandflies were collected from peri-domestic and domestic habitats and identified using standard taxonomic keys. *Phlebotomus* species were subjected to molecular detection of *Leishmania donovani* using PCRs targeting kinetoplast DNA and Internal Transcribed Spacer-I markers. The ITS-I products were subjected to restriction fragment length polymorphism and custom-sequenced for species characterisation. nBLAST and phylogenetic analyses confirmed the species, and the ITS-I sequences were submitted to NCBI-GenBank. Barcoding of the mitochondrial cytochrome oxidase I gene confirmed the sandfly species.
A total of 6,278 specimens belonging to 29 species from 3 genera (*Phlebotomus*, *Sergentomyia* and *Grassomyia*) were morphologically identified. 18,31% individual and 40,27% pool positivity were observed among the 6 species of *Phlebotomus* analysed for infection with *Leishmania donovani*.
The distinct distribution pattern of certain sandfly species and the wide distribution of *Phlebotomus argentipes* facilitate the transmission of leishmaniasis throughout the country. The current findings can be incorporated into leishmaniasis vector control programmes. These findings can be further explored to determine the vector competence of these naturally infected sandflies.

Keywords: Phlebotomine sandflies, Leishmaniasis, *Leishmania donovani*, PCR, Vector

O23 Comparative analysis of *Leishmania*-infected sand flies in their microhabitats reveal the complexity of visceral leishmaniasis transmission in East Africa

Iniguez Eva[1], Tuluso Mercy[2], Kiplagat Steve[3], Gebresilassie Araya[4], Aklilu Esayas[5], Battistoni Olivia[6], Ingonga Johnstone[2], Makwatta John Mark[3], Dakien Osman M.[7], Chebet Alphine[2], Kaunda Esther[2], Huffcutt Patrick[1], Doh Serena[1], Osman Omran F.[7], Younis Brima M.[7], Zhou Guofa[8], Masiga Dan[3], Musa Ahmed M.[9], Hailu Asrat[10], Satoskar Abhay[6], Matoke Damaris[2], Kamhawi Shaden[1]*

[1]Vector Molecular Biology Section, Laboratory of Malaria and Vector Research, National Institute of Allergy and Infectious Diseases, National Institutes of Health, Rockville, MD, USA
[2]Centre for Biotechnology Research and Development, Kenya Medical Research Institute, Nairobi, Kenya
[3]International Centre of Insect Physiology and Ecology (icipe), Nairobi, Kenya
[4]Department of Zoological Sciences, College of Natural and Computational Sciences, Addis Ababa University, Addis Ababa, Ethiopia
[5]Vector Biology & Control Research Unit, Aklilu Lemma Institute of Pathobiology, Addis Ababa University, Addis Ababa, Ethiopia
[6]Departments of Pathology and Microbiology, Wexner Medical Center, The Ohio State University, Columbus, OH, USA
[7]Department of Zoology, University of Khartoum, Khartoum, Sudan
[8]Program in Public Health, University of California at Irvine, Irvine, CA, USA
[9]Department of Clinical Pathology and Immunology, Institute of Endemic Diseases, University of Khartoum, Khartoum, Sudan
[10]College of Health Sciences, Addis Ababa University, Addis Ababa, Ethiopia

***Corresponding author:** skamhawi@nih.gov

Abstract

East Africa is the epicenter of global visceral leishmaniasis (VL) transmission, particularly affecting Sudan, Kenya, and Ethiopia which have reported 60.4% of cases worldwide. In these countries, VL is caused by *Leishmania donovani* and is transmitted by multiple sand fly species with divergent ecologies. Significant knowledge gaps related to ecoepidemiology, vector biology, and disease reservoirs retard control efforts in this region. To understand the main drivers of VL transmission in East Africa, we undertook clinical and entomological studies in Sudan, Kenya, and Ethiopia. In Kenya and Sudan, preliminary findings confirmed the prevalence of antibodies to *Leishmania* rK39 antigen in recently treated VL cases, as well as in asymptomatic subjects in our study sites. Importantly, using qPCR on individually screened sand flies, we found 13/310 (4.2 %) *Ph. orientalis* in Kenya, and 1/67 (1.5%) *Ph. martini/celiae* in Ethiopia, infected with *Leishmania*. In Sudan, 1/5 pools of 10 (20%) *Ph. orientalis* were infected. Notably, infected specimens were predominantly collected from vegetation such as *Acacia* trees that were associated with vertisol cracks and animal burrows in Kenya, from a termite hill in Ethiopia, and near animal enclosures in Sudan, indicating that transmission is focal and occurs in distinct microhabitats within the three countries. We are continuing to conduct comprehensive longitudinal surveys of sand fly microhabitats to map hot beds of active transmission and identify reservoir hosts towards facilitating a rapid response to disease outbreaks and a targeted VL control strategy.

Keywords: East Africa, visceral leishmaniasis, infected sand flies, transmission

O24 Sympatric occurrence of *Leishmania infantum* and *Leishmania tarentolae* in *Sergentomyia minuta* sand flies

Carbonara Mariaelisa[1], Bezerra Santos Marcos Antonio[1], Mendoza-Roldan Jairo Alfonso[1], Perles Lívia[1], Indraccolo Francesca[2], Iatta Roberta[2], Volf Petr[3], Otranto Domenico[1,4]*

[1]*Department of Veterinary Medicine, University of Bari, Italy*
[2]*Interdisciplinary Department of Medicine, University of Bari, Bari, Italy*
[3]*Department of Parasitology, Faculty of Science, Charles University, Czech Republic*
[4]*Department of Veterinary Clinical Sciences, City University of Hong Kong, China*

***Corresponding author:** domenico.otranto@uniba.it

Abstract

Phlebotomine sand flies are insects of medical and veterinary importance being vectors of several zoonotic pathogens, with *Leishmania* spp. as the most relevant. Despite studies about the sand fly vector role are available, knowledge gaps persist on the epidemiological dynamics driven by the sympatric occurrence of *Leishmania infantum* and *Leishmania tarentolae* in canine leishmaniasis endemic areas (i.e., Southern Italy). Therefore, this study aimed to detect and isolate *L. infantum* and *L. tarentolae* from unproven vectors (i.e., *Sergentomyia minuta* and *Phlebotomus perniciosus,* respectively). From May to October 2023, sand flies were collected in six sampling sites in Southern Italy using CDC light traps. Alive females were dissected to determine the presence of flagellates. All the specimens were morphologically identified, females were screened for *Leishmania* DNA, and engorged ones for blood-meal detection. Sand flies collected (i.e., n=642; n=503 females; n=139 males) were identified as *Ph. perniciosus* (n=275), *Phlebotomus perfiliewi* (n=92), *Phlebotomus neglectus* (n=16), and *S. minuta* (n=259). Though flagellates were not observed at the dissection of alive females, *L. infantum* DNA was detected in *Ph. perniciosus* (n=3/182; 1.6%), *Ph. perfiliewi* (n=1/66; 1.5%) and *S. minuta* (n=5/248; 2%). In addition, *L. tarentolae* DNA was detected in *S. minuta* (n=11/248; 4.4%) specimens, along with human DNA in engorged *S. minuta* females. Data suggest that when *L. infantum* and *L. tarentolae* occur in sympatry, the herpetophilic *S. minuta*, the proven vector of *L. tarentolae,* may be infected by *L. infantum* and feed on mammals. Hence, future efforts, focusing on the protozoa isolation, will provide a better understanding on the new potential *Leishmania* spp. pathways.

Keywords *Sergentomyia minuta*, *Phlebotomus perniciosus*, *Leishmania infantum*, *Leishmania tarentolae*

O25 Co-circulation of *Leishmania infantum* and Toscana virus in sandflies and dogs in a focus of canine leishmaniasis of Northern Tunisia

Dachraoui Khalil[1,2], Ben Osman Raja[3], Labidi Imen[1], Sayadi Aida[3], Chelbi Ifhem[1,2], Boussoffara Thouraya[4], Trimèche Malek[4], Maachach Yassine[1], Ben Said Mourad[5], Cherni Saifedine[1,2], Abdo Saghir Abbas Mohammed[1], Zhioua Elyes[1,2]*

[1]*Institut Pasteur de Tunis, Unit of Vector Ecology, Tunis, Tunisia*
[2]*Institut Pasteur de Tunis, Laboratory of Epidemiology and Veterinary Microbiology, Tunis, Tunisia*
[3]*Vaccine Control Unit, National Drug Control Laboratory, Tunis, Tunisia*
[4]*Laboratory of Transmission, Control, and Immunobiology of Infections. Pasteur Institute of Tunis. University of Tunis El Manar, Tunis, Tunisia*
[5]*Department of Basic Sciences, Higher Institute of Biotechnology of Sidi Thabet, University of Manouba, Manouba, Tunisia*

***Corresponding author:** elyes.zhioua@gmail.com

Abstract

We investigated *Leishmania infantum* and Toscana virus infection in sandflies and in dogs within a focus of canine leishmaniasis located in Northern Tunisia from June to October 2020. Sandflies were collected on a weekly basis during the study period by sticky traps and identified to species level. *Phlebotomus perniciosus* was the most abundant species (41.94%), followed by *P. perfiliewi* (31.36%), *Sergentomya minuta parotti* (26.19%), *P. papatasi* (0.36%), and *P. longicuspis* (0.12%). The phenology of sandflies of the subgenus *Larroussius*, showed two main peaks: a small one in June and a second larger one in September–October. A total of 6,211 live sandflies were captured by CDC light traps and tested for the presence of *L. infantum* and TOSV by PCR. The infection rates of sandflies with *L. infantum* and TOSV were 0.053% (2/3730) and 0.09% (6/6211), respectively. TOSV-positive pools were detected in both females and males sandflies. It is important to note that *L. infantum* and TOSV were detected during the second main peak of sandfly activity. At the end of the exposition period, dogs were examined for TOSV and *L. infantum* infections by xenodiagnosis and by serology. *Leishmania infantum* DNA and TOSV RNA were detected in *P. perniciosus* females fed on two infected dogs. Isolation of TOSV in Vero cells was achieved from two pools containing *P. perniciosus* fed on infected dog. These findings provide strong evidence that in addition to their role as the main reservoir of *L. infantum*, dogs act as reservoir hosts for Toscana virus.

Keywords: co-circulation, *Leishmania infantum*, Toscana virus, reservoir, dogs

O26 An innovative toolbox aims to identify leishmaniasis reservoirs through analysis of individual field-collected blood fed sand flies

Iniguez Eva[1], Huffcutt Patrick[1], Meneses Claudio[1], Doh Serena[1], Pugh Aaron[1], Serafim Tiago D.[1], Valenzuela Jesus G.[1], Kamhawi Shaden[1]*

[1]*Vector Molecular Biology Section, Laboratory of Malaria and Vector Research, National Institute of Allergy and Infectious Diseases, National Institutes of Health, Rockville, MD, USA*

***Corresponding author**: skamhawi@nih.gov

Abstract

In many active foci of leishmaniasis, the identity of reservoirs and their relative significance in maintenance and dissemination of disease remains uncertain. To enable unmanipulated identification of leishmaniasis reservoirs, and to elucidate mechanics of parasite transmission, we are developing a field-applicable toolbox based on analysis of DNA and RNA co-extracted from single blood fed sand fly midguts. Presently, we have optimized coextraction of an average of 858ng DNA and 867ng RNA per midgut preserved on an FTA card. We used DNA to identify low level *Leishmania* infections, using a sensitive probe-based qPCR targeting the kinetoplast, and the blood source, using a multiplex PCR based on several mitochondrial genes. The current blood meal host panel includes up to 10 species and can be tailored to domestic and sylvatic animals of interest. Using in vivo experimental infections, we validated the use of the constitutively expressed *ssrRNA* gene to accurately quantify live parasites from the extracted RNA. Using a bulk-RNA seq approach, we discovered novel hypothetical *Leishmania* genes with potential as stage-specific targets that can distinguish the early parasite forms present in the initial infected blood meal from those prevalent in subsequent sand fly feeds. We are currently validating the toolbox on field-collected sand flies. This adaptable combination of molecular tools, used in the context of sand fly behavior, enables reliable identification of leishmaniasis reservoirs, and the accurate assessment of the focality and intensity of ongoing transmission towards deployment of a more targeted control strategy in active and emerging foci of leishmaniasis.

Keywords: *Leishmania*-infected sand fly, Blood fed, transmission, reservoir host

O27 Invertebrate-Derived DNA (iDNA) to identify sandflies' bloodmeal

Cova Bruno Oliveira[1]*, Saranholi Bruno[2], Gestich Carla[2], Monte-Alegre Adriano[3], Machado Paulo Roberto[1], Schriefer Albert[1]

[1]Immunology Service, Professor Edgard Santos Hospital Complex (COM-HUPES), Federal University of Bahia (UFBA), Brazil
[2]Laboratory of Molecular Biodiversity and Conservation, Center for Biological and Health Sciences, Federal University of São Carlos, São Paulo, Brazil (LBMC-UFSCar)
[3]Laboratory of Hematophagous Insects, Health Sciences Institute, Federal University of Bahia (UFBA), Brazil

***Corresponding author:** bruno_cova@yahoo.com.br

Abstract

Metabarcoding data obtained by Next Generation Sequencing (NGS) has been used to determine the species in mixed biological samples, like DNA obtained through the gut content of invertebrates that feed on vertebrates (invertebrate-derived DNA, iDNA). In the present study, we identified the vertebrate species from iDNA samples of female sand flies using the metabarcoding approach. We also compared the results among a mammal-specific (16SrRNA), broader vertebrate-specific (12SrRNA) mini-barcodes, and a barcode of the *CytB* mitochondrial gene. Sand flies were collected in an endemic area of Cutaneous Leishmaniasis (CL) located in the Cacao Region, southeastern of Bahia, Brazil. We analyzed forty female sand flies distributed in thirteen samples of seven different sand fly species including CL vectors in Brazil. The Operational Taxonomic Units (OTUs), obtained after metabarcoding sequencing, were compared with the sequences available in the GenBank NCBI® for species identification. The criteria of high percentage of matches (98%-100%) was used for OTU assignment at species level, combined to reference databases of the expected species occurrence in the study area. Metabarcoding results revealed twenty-one OTUs of vertebrate's species, distributed in forty GenBank® sequences, including primates (four OTUs), rodents (four) and ungulates (six). Surprisingly, non-mammal species, such as reptiles (one OTU) and amphibians (three) were also detected. The mini-barcode 16SrRNA identified twelve OTUs in 69 detections, 12SrRNA and CytB eleven OTUs in 30 and 38 detections, respectively. *Canis lupus*, *Equus asinus*, *Equus caballus* and *Homo sapiens* were the sandflies' bloodmeal identified by the three targets used, presenting the highest ratio of detection.

Keywords: Phlebotominae, metabarcoding, *Leishmania*

O28 Establishment of an In-house library for host blood identification and blood meal analysis of a natural population of Moroccan *Phlebotomus sergenti* using a proteomic approach

El Kacem Sofia[1,4]*, Marchand Lucien[1], Ouali Radouane[1], Sadlova Jovana[2], Vojtkova Barbora[2], Dvořák Vít[2], Volf Petr[2], Boussaa Samia[3], Lemrani Meryem[4], Bousbata Sabrina[1]

[1]*Laboratory of Microbiology, Department of Molecular Biology, Université Libre de Bruxelles, 6041 Gosselies, Belgium.*
[2] *Department of Parasitology, Faculty of Science, Charles University, Prague, Czech Republic.*
[3]*Higher Institute of Nursing and Technical Occupations Health, Rabat, Morocco.*
[4]*Laboratory of Parasitology and Vector-Borne Diseases, Institut Pasteur du Maroc, Casablanca, Morocco.*

***Corresponding author:** sofiaelkacem@gmail.com

Abstract

Phlebotomus sergenti is the main vector of *Leishmania tropica*, the causative agent of anthroponotic cutaneous leishmaniasis (ACL) in Morocco. ACL represents the most common form in the country, with a wide geographical distribution. Controlling leishmania's transmission cycle requires knowledge of the potential hosts and trophic preferences of sandfly vectors. The main objective of this study was to create a database that catalogs the major blood proteins of Moroccan hosts and query it to identify the blood meal source of *Ph. sergenti* engorged females collected in Taza, an endemic area of leishmaniasis in northern Morocco. We developed a streamlined library of major blood proteins of potential Moroccan host animals by compiling blood protein sequences from 30 Moroccan hosts. Protein sequence extraction from UniProt was automated using Python and Shell scripts then Emboss. Blood meals in engorged females were analyzed by Liquid Chromatography coupled with Tandem Mass Spectrometry (LC-MS/MS) and data analysis was performed with MaxQuant software. Spectra were searched against the different hosts proteins in the in-house database which contains 7138 sequences. This was done instead of querying the entire UniProt database, which contains over 245 million protein sequences. Preliminary results of the analyzed blood meals of *Ph. sergenti* indicate that chicken (*Gallus gallus*) is a predominant host with 46% of species-specific sequences out of 146 identified sequences. The reduction in database size significantly shortened analysis time in MaxQuant from several days to a few hours. The database was validated using laboratory-reared sandflies.

Keywords: *Phlebotomus sergenti*, blood meal, in-house library, Mass Spectrometry, Morocco

*O*29 *Asaia* bacteria in sand flies and their impact on *Leishmania* transmission

Stejskalova Marketa[1]*, Jancarova Magdalena[1], Varotto-Boccazzi Ilaria[2], Epis Sara[2], Volf Petr[1]

[1]*Department of Parasitology, Faculty of Science, Charles University, Prague, Czech Republic*
[2]*Department of Biosciences and Pediatric Clinical Research Center "Romeo ed Enrica Invernizzi", University of Milan, Milan, Italy*

***Corresponding author**: marketa.stejskalova@natur.cuni.cz

Abstract

Midgut microbiome was demonstrated to affect the transmission of vector-borne pathogens. In sand flies, *Asaia* bacteria was found as a part diet and midgut microbiome. Here, we have investigated the effect of two *Asaia* species on the development of *Leishmania major* in *Phlebotomus duboscqi.* The sand flies were first infected with bacteria via sugar meal and then membrane-fed on blood containing *Leishmania* promastigotes. Following this superinfection, the development of *Leishmania* infection was examined. Particularly, we studied changes in localization and intensity of infection and examined *Leishmania* morphological forms on midgut smears. Both tested bacteria species, *Asaia siamensis*, and *Asaia krungthepensis*, colonized the intestine of female *Ph. Duboscqi* for up to 8 days after infection and were transmitted vertically to the next generation through contamination of the egg surface. The presence of *Asaia* within *Ph. Duboscqi* negatively affects the intensity of *Leishmania* late-stage infections. In addition to the wild type, we tested a strain of *Asaia* engineered for the expression of a protein of *Wolbachia* (WSP). This strain of *Asaia* also readily survives in *Ph. duboscqi* midgut and experiments on its effect on *Leishmania* infection are in progress.

Keywords: *Phlebotomus*, *Asaia*, superinfection, microbiome

O30 Evaluation of biological parameters of female *Lutzomyia longipalpis* (Diptera: Psychodidae) after contact with insecticide-impregnated screens

Cardoso de Oliveira Augusto[1], Bacelar Paixão Ignacio Matheus[1], Ferreira Bonfim Isabel[1], Liu Kevin[1], Albuquerque e Silva Rafaella[1], Pinto Mara Cristina[1]*

[1]*São Paulo State University (UNESP), School of Pharmaceutical Sciences, Araraquara, Brazil*

***Corresponding author:** mara.pinto@unesp.br

Abstract

In Brazil, nets impregnated with long-lasting insecticides (LLINs) are used to protect against malaria, but there are few studies on leishmaniasis. Previous studies have shown that sandflies penetrate these LLINs. We want to evaluate the effect of two meshes on mortality, blood feeding and oviposition of *Lutzomyia longipalpis* females. The inner parts of the plastic pots were covered with the meshes: Interceptor® G1 (alpha-cypermethrin), Interceptor® G2 (chlorfenapyr and alpha-cypermethrin) and without insecticide (control). In triplicate for each group, 10 *Lu. longipalpis* females were placed in the pots for 15 minutes and at the end the females were transferred to three cages. Blood feeding was performed with mice and oviposition was observed for up to seven days. After 15 minutes, the insects in the control group showed no behavioural changes. Females exposed to G1 became lethargic and 30 insects suffered a knock-down, making it impossible to observe blood feeding in this group; in G2, only four insects did not suffer a knock-down. Blood feeding was performed on the insects in the control group and group G2 that had not suffered a knock-down. In the control group, 19 females had fed blood (63%). Of the four females in the G2 group, only one was able to feed (25%). In the control group 48 eggs were laid and in the G2 group there was no oviposition. Future tests will be carried out to find the shortest exposure time at which the biological parameters of the females are still unchanged.

Keywords: control, bednets, insecticides

O_{31} Insecticide impregnated collars for the control of visceral leishmaniasis: evaluation of the susceptibility of *Lutzomyia longipalpis* to deltamethrin

Sousa Félix de Lima Mayra[1], Albuquerque Rafaella e Silva[1,2]*, Almeida Rocha Douglas[2], Oliveira Mosqueira Gabriela[1], Gurgel-Gonçalves Rodrigo[1], Takashi Obara Marcos[1]

[1]*University of Brasilia, Center for Tropical Medicine, Brasília, DF, Brazil*
[2]*Ministry of Health, Health Surveillance Secretariat, Brasília, DF, Brazil*

***Corresponding author:** rafaas28@gmail.com

Abstract

The use of collars impregnated with 4% deltamethrin as a tool for controlling visceral leishmaniasis may contribute to the emergence of resistance in sandflies. The objective was to characterize the susceptibility profile of different *Lu. longipalpis* populations to deltamethrin to pyrethroid in areas using impregnated collars for VL control. The field populations came from the municipalities of Foz do Iguaçu/PR (FOZ), Teresina/PI (TER), Fortaleza/CE (FOR), Caucaia/CE (CAU), Montes Claros/MG (MOC) and Cavalcante/GO (CAV), which were collected with CDC traps installed in the peridomicile for three consecutive nights. Wheaton bottles of 250 ml with Diagnostic Dose (DD) of 21.9 µg/bottle and 30 µg/bottle and doses of 1, 3, 5, 7, 9 and 11 µg/bottle were used for Diagnostic Dose (DD) and Dose-Response (DR) bioassays, respectively. The controls were impregnated only with acetone. An average of 20 sandflies (♂♂ and ♀♀ mixed) were used per bottle and the exposure time was 60 minutes. The mortality reading was performed 24 hours by a single researcher. A total of 4,094 sandflies were used in the CDC bottle bioassays. *Lu. longipalpis* was the most collected species in all localities (94%). For DD of 21.9 µg/bottle, the populations from the municipalities of FOZ, MOC, CAV and TER were susceptible, while CAU presented a mortality of 87.1%, indicating resistance and FOR showed a value of 94.9%, suggesting possible resistance. It is concluded that the majority of *Lu. longipalpis* were susceptible to the pyrethroid deltamethrin in areas with the use of impregnated collars.

Keywords: *Lutzomyia longipalpis*, visceral leishmaniasis, Resistance

O_{32} Assessment of the impact of deltamethrin-impregnated collars on the density of *Lutzomyia longipalpis* in an endemic area of visceral leishmaniasis in Brazil

Galvis-Ovallos Fredy[1]*, Andrade do Rosario Amanda[2], Fonseca Rocha Marilia[3], Posada Lopez Laura[2], Oliveira Alessandra[4], Galati Eunice[1], Socorro Cruz Maria[5], Figueredo Fabiano[6], Werneck Guilherme[7]

[1]*Department of Epidemiology, Faculty of Public Health, University of São Paulo, Brazil*
[2]*Postgraduate-program in Public Health, Faculty of Public Health, University of São Paulo, Brazil*
[3]*Zoonosis control center, Montes Claros-MG, Brazil*
[4]*Federal University of Mato Grosso do Sul, Brazil*
[5]*Federal University of Piaui, Brazil*
[6]*Carlos Chagas Institute – Fiocruz/Paraná, Brazil*
[7]*Rio de Janeiro State University and Federal University of Rio de Janeiro, Brazil*

***Corresponding author:** fgalvis@usp.br

Abstract

Deltamethrin-Impregnated Collars have been integrated into Brazil's national program for preventing and controlling visceral leishmaniasis since 2021, particularly targeting priority areas according the Pan-American Health Organization (PAHO) stratification. This study aimed to evaluate the impact of this intervention on the overall density and the proportion of engorged females of *Lutzomyia longipalpis* populations. The research was conducted across two regions in Montes Claros, MG, selected for their similar environmental and social characteristics. Each area was georeferenced and 10 blocks were randomly chosen, with one domicile selected per block using convenience sampling. Two light traps were set up monthly for three consecutive days in each chosen domicile between August/2021 and August/2023. A total of 21,322 specimens were captured, with 9,902 (8,015 males and 1,887 females) in the control area and 11,420 (9,347 males and 2,073 females) in the intervention area, indicating a male-to-female ratio of 4.4:1. No significant differences in female density were observed between the regions (U = 303, p = 0.854). The number of engorged females was lower in the intervention area; however, the difference was not statistically significant (U = 303, p = 0.854). Our findings suggest that the intervention with DM4% collars does not influence the density of Lu. longipalpis nor the prevalence of engorged females. Our results suggest that the intervention with DM4% does not affect the density of *Lu. longipalpis*, nor the number of engorged females. This information, along with other ecological parameters, could be useful for evaluating the implementation of DM4% collars aiming to improve the planning of prevention and control programs for VL.

Keywords: *Lutzomyia longipalpis*, DM4% collar, intervention, visceral leishmaniasis, Brazil

O33 Preliminary results of Insecticide resistance bioassay against synthetic pyrethroids on sandflies in the west part of Türkiye

Yetismis Kardelen[1,4]*, Arserim Suha Kenan[2]., Pekagirbas Metin[3], Toz Seray[4], Ozbel Yusuf[4], Sanjoba Chizu[5]

[1]*Department of Parasitology, Institute of Health Science, Ege University, Izmir, Türkiye*
[2]*Vocational School of Health Sciences, Manisa Celal Bayar University, Manisa, Türkiye*
[3]*Department of Parasitology, Faculty of Veterinary Medicine Aydın Adnan Menderes University, Aydın, Türkiye*
[4]*Department of Parasitology, Faculty of Medicine Ege University, Izmir, Türkiye*
[5]*Laboratory of Molecular Immunology, Graduate School of Agriculture and Life Sciences University of Tokyo, Tokyo, Japan*

***Corresponding author:** kardelenyetismis@gmail.com

Abstract

Synthetic pyrethroids are known to be used for a long time in vector control programmes in Turkey. Here we present the preliminary results of a susceptibility test conducted on wild-caught adult sand flies in Akbük town.
Filter papers were impregnated with permethrin 1% and deltamethrin 0.05% and control papers were impregnated with the suggested solvent to be used in the tube tests according to WHO guidelines. Sand flies were collected using CDC light traps. Active females were placed into the test and control tubes in groups of 25±5 using a mouth aspirator. For each insecticide, tests were repeated four times, and a control group was used for each insecticide. Samples were held in the test tubes for one hour, then transferred to holding tubes and observed for 12 hours. Live, dead and knocked-down individuals were counted hourly until the sixth hour and finally at the twelfth hour. Live samples are being stored in a -80°C freezer for future mutation screening on the voltage-gated sodium channel (VGSC) gene responsible for resistance to pyrethroids.
The sand flies tested against permethrin 1% all died within the first three hours of exposure. On the other hand, it was found that some of the samples exposed to deltamethrin 0.05% were knocked-down by the 6th hour but were still alive on the 12th hour.
The results obtained indicate that; there is a need to perform mutation analyses for prolonged knock-down values for Deltamethrin, and Permethrin is still a valid insecticide for vector control applications in this region.

Keywords: Sand fly, insecticide, resistance, pyrethroid

O34 Providing a better knowledge and comprehension of climate and environmental drivers of sand fly-borne diseases - the CLIMOS project

Maia Carla[1]*, Altug Unal[2], Athanatos Manos[3], Berriatua Eduardo[4], Blesic Suzana[5], Bongiorno Gioia[6], Charrel Remi[7], Courtenay Orin[8], Davidovich-Cohen Maya[9], Depaquit Jerome[10], Dvorak Vit[11], Erisoz Ozge[12], Ferraro Federica[13], Fleming Padraic[14], Foglia-Manzillo Valentina[15], Ferreira Maia Maria Joao[16], Gligoric Nenad[17], Gligorijevic Vladan[18], Guardado Diana[19], Hamilton Gordon[20], Hempelmann Nils[21], Hatzakis Tally[14], Ivovic Vladimir[22], Kirstein Oscar[9], Kniha Edwin[23], Kolarova Iva[11], Ozbel Yusuf[24], Paz Shlomit[25], Robert-Gangneux Florence[26], Sadlova Jovana[11], Samaniego Luis[27], San Martin Daniel[28], Van Langevelde Frank[29], Volf Petr[11], Wright David[14]

[1]University Nova of Lisbon, Lisbon, Portugal, [2]Turkish Ministry of Health, Ankara, Turkey, [3]Polytechneio Kritis, Chania, Greece, [4]University of Murcia, Murcia, Spain, [5]Institute for Medical Research, University of Belgrade, Belgrade, Serbia, [6]Italian Institute of Health, Rome, Italy, [7]Aix-Marseille University, Marseille, France, [8]University of Warwick, Coventry, United Kingdom, [9]Israeli Ministry of Health, Jerusalem, Israel, [10]University of Reims Champagne-Ardenne, Reims, France, [11]Charles University, Prague, Czech Republic, [12]Hacettepe University, Ankara, Turkey, [13]Ministry of Health, Rome, Italy, [14]Trilateral Research, Waterford, Ireland, [15]University of Naples Federico II, Naples, Italy, [16]Karlsruhe Institute of Technology, Karlsruhe, Germany, [17]Zentrix Lab, Pancevo, Serbia, [18]CubexLab, Amsterdam, Netherlands, [19]F6S Network, Ireland Limited, Dublin, Ireland, [20]Lancaster University, Lancaster, United Kingdom, [21]Open Geospatial Consortium, Leuven, Belgium; [21]University of Primorska, Koper, Slovenia, [23]Medical University of Vienna, Vienna, Austria, [24]Ege University, Izmir, Turkey, [25]University of Haifa, Haifa, Israel, [26]University of Rennes 1, Rennes, France, [27]Helmholtz Centre for Environmental Research, Leipzig, Germany, [28]Predictia, Santander, Spain, [29]Wageningen University, Wageningen, Netherlands

***Corresponding author:** CarlaMaia@ihmt.unl.pt

Abstract

Sand fly-borne diseases, including leishmaniasis and phleboviruses, represent a major public health and veterinary concern. The spread of sand fly vector populations and the pathogens they transmit has induced in recent decades several research consortia to improve knowledge, surveillance and control in Europe and neighbouring countries.

The CLIMOS project (Climate Monitoring and Decision Support Framework for the Detection and Mitigation of Sand Fly Diseases with Cost-Benefit and Climate Policy Measures; http://www.climos-project.eu), brings together 29 partners, including universities, institutes, research centers and ministries of health from 16 countries within and outside Europe. It aims to characterize the climatic, environmental, demographic, and epidemiological characteristics associated with the presence and abundance of sand flies and domestic animal infection rates at different geographic scales across Europe and neighbouring countries.

These data will feed into mathematical epidemiological-climate prediction models of realistic human-induced climate change scenarios to help develop an early warning system for infection and disease designed for public use seeking to better prepare for current and future impacts of climate and environmental change on human and animal health.

Funding: The CLIMOS consortium is co-funded by the European Commission grant 101057690 and UKRI grants 10038150 and 10039289. The six Horizon Europe projects, BlueAdapt, CATALYSE, CLIMOS, HIGH Horizons, IDAlert, and TRIGGER, form the climate change and health cluster.

Keywords: Phlebotomine sand flies; *Leishmania*, phleboviruses, climate changes, early warning system

O35 Sand fly sample collection and analysis

Erisoz Kasap Ozge[1], Adam Katja[2], Alcover Magdalena[3], Arserim Suha Kenan[4], Bañuls Anne-Laure[5], Barandika Jesús[6], Basseville François[5], Bernardini Ilaria[7], Berriatua Eduardo[8], Bianchi Riccardo[7], Bisia Marina[9], Bongiorno Gioia[7], Bouhsira Emilie[10], Cevidanes Miranda Aitor[6], Cuadrado-Matías Raúl[11], Dardé Marie-Laure[12], Delaunay Pascal[13], Delacour Estrella Sarah[14], Depaquit Jerome[15], Díaz-Sáez Victoriano[16], Falchi Alessandra[17], Favennec Loïc[18], Fernández Guillermo[14], Fisa Roser[3], Robert-Gangneux Florence[19], Ivovic Vladimir[2], Izri Arezki[20], Jiménez Maribel[21, 22], Kirstein Oscar[23], Kniha Edwin[24], La Russa Francesco[25], Liénard Emmanuel[10], Lucientes Curdi Javier[14], Mangiapelo Claudia[7], Martín-Martín Inés[23], Martín-Sánchez Joaquina[16], Martínez-Barciela Yasmina[26], Mathieu Bruno[27], Mekarnia Nalia[15], Mercier Aurélien[12], Mhaidi Idris[5], Michaelakis Antonios[9], Molina Ricardo[21, 22], Morales-Yuste Manuel[16], Ozbel Yusuf[28], Paz Adolfo[29], Pekagirbas Metin[30], Pérez-Cutillas Pedro[8], Platzgummer Katharina[24], Polina Alejandro[26], Pomares Christelle[13], Prudhomme Jorian[19], Randrianambinintsoa Fano[15], Risueño José[8] Roca-Geronès , Xavier[3], Ruiz-Fons Francisco[11], Sánchez Andrade Rita[29], Sevila Julie[17], Toz Seray[28], Yetismis Kardelen[30], Maia Carla[31], Dvorak Vit[32]*

[1]Department of Biology, Faculty of Science, Hacettepe University, Ankara, Turkey; [2]Department of Biodiversity, FAMNIT, University of Primorska, Koper, Slovenia; [3]Departament de Biologia, Sanitat i Medi Ambient, Facultat de Farmàcia i Ciències de l'Alimentació, Universitat de Barcelona, Barcelona, Spain; [4]Department of Parasitology, Faculty of Medicine, Ege University, Izmir, Turkey; [5]UMR MIVEGEC, IRD-CNRS, University of Montpellier, Montpellier, France; [6]Basque Institute for Agricultural Research and Development (NEIKER), Derio, Spain; [7]Department of infectious diseases, Istituto Superiore di Sanità, Rome, Italy; [8]Department of Animal Health, Faculty of Veterinary Science, University of Murcia, Murcia, Spain; [9]Laboratory of Insects and Parasites of Medical Importance, Benaki Phytopathological Institute, Attica, Greece; [10]InTheres, University of Toulouse, INRAE, ENVT, Toulouse, France; [11]Spanish Game & Wildlife Research Institute (IREC), Ciudad Real, Spain; [12]EpiMaCT,,Institute of Epidemiology and Tropical Neurology, OmegaHealth, Limoges, France; [13]Department of Parasitology, University of Nice Côte d'Azur, Nice, France; [14]Department of Animal Pathology, Faculty of Veterinary Medicine, University of Zaragoza, Spain; [15]UR Escape, University of Reims Champagne-Ardenne, Reims, France; [16]Department of Parasitology, Faculty of Pharmacy, University of Granada, Granada, Spain; [17]Department of Emerging Viruses, IRD-INSERM-IRBA, University of Aix-Marseille, University of Corsica, France; [18]UR Escape, French National Cryptosporidiosis Reference Center, University of Rouen Normandy, Rouen, France; [19]Université de Rennes, Inserm, EHESP, Irset (Institut de Recherche en Santé Environnement Travail), Rennes, France; [20]Department of Parasitology-Mycology, Avicenne Hospital, Bobigny, France; [21]Medical Entomology Laboratory, National Center for Microbiology, Instituto de Salud Carlos III, Majadahonda, Madrid, Spain; [22]Collaborative Biomedical Research Center in Infectious Diseases (CIBERINFEC), Instituto de Salud Carlos III, Madrid, Spain; [23]Medical Entomology and Parasitology, Laboratory, Israeli Ministry of Health, Jerusalem, Israel; [24]Institute of Specific Prophylaxis and Tropical Medicine, Medical University of Vienna, Vienna, Austria; [25]Laboratory of Entomology, Istituto Zooprofilattico Sperimentale della Sicilia, Palermo, Italy; [26]Departamento de Ecoloxía e Bioloxía Animal, Universidade de Vigo, Pontevedra, Spain; [27]Institutes of Bacteriology and Parasitology, Medical Faculty, University of Strasbourg, Strasbourg, France; [28]Department of Parasitology, Faculty of Medicine, Ege University, Izmir, Turkey; [29]Control of Parasites Group (COPAR, GI-2120), Department of Animal Pathology, Faculty of Veterinary, University of Santiago de Compostela, Lugo, Spain; [30]Department of Parasitology, Faculty of Veterinary Medicine, Adnan Menderes University, Aydin, Turkey; [31]Instituto de Higiene e Medicina Tropical, University Nova of Lisbon, Lisbon, Portugal; [32]Faculty of Science, Department of Parasitology, Charles University, Prague, Czech Republic

***Corresponding author:** vit.dvorak@natur.cuni.cz

Abstract

CLIMOS project studies climatic, environmental, demographic, and epidemiologic characteristics associated with sand fly presence and abundance, as well as animal infection rates, at different geographical scales across Europe and neighbouring countries. It uses historical datasets (EDEN, EDENext, VBORNET, and VectorNet) and active sand fly field surveillance in a coordinated and standardized effort, followed by screening for sand fly-borne pathogens (*Leishmania* sp. and phleboviruses). This data feeds into epidemiological-climatic predictive mathematical models of realistic human-induced climatic changes scenarios to help develop an early warning system for infection and disease, designed with the input from partner public health ministries for public use.
A coordinated and standardized sampling effort using a common protocol for sample acquisition and processing based on the Standard Operating Procedures was performed in 11 countries during the 2023 and 2024 active sand fly seasons (April-November). In 2023, 117 sampling sites were surveyed with the total trapping effort 3790 trap/nights for temporal data collections in Portugal, Spain, France, Italy, Slovenia, Croatia, Germany, Austria, Czech Republic, Turkey and Israel using a common trapping method by CDC light traps. Moreover, sand fly bycatch was obtained from a partner project, IDAlert, which sampled 65 sites in Greece by BG traps. Climatic and environmental data at the sites were collected and spatial sand fly data was collected from additional prospective sites in several countries. Twenty-two sand fly species of the genera *Phlebotomus* (species of 7 subgenera) and *Sergentomyia* were recorded among the surveyed countries and identified by standard protocol integrating morphological and molecular approaches.

Keywords: sand fly, field surveillance, spatial data, temporal data, species identification

O36 *Leishmania* and Phlebovirus detection in sand flies and canine sera samples

Bongiorno Gioia[1]*, Ayhan Nazli[2], Robert Gangneux Florence[3], Adam Katja[4], Alcover M. Magdalena[5], Alten Bulent[6], Anne-Laure Bañuls[7], Barandika, Jesús F[8], Basseville François[7], Bernardini Ilaria[1], Berriatua Eduardo[9], Bouhsira Emilie[10], Cevidanes Miranda Aitor[8], Charrel Remi[2], Cristóvão Jose[11], Cuadrado-Matías Raúl[12], Dardé Marie-Laure[13], Delabarre Aymeric[3], Delacour Estrella Sarah[15], Delaunay Pascal[16], Depaquit Jerome[14], Di Muccio Trentina[1], Díaz-Sáez Victoriano[17], Erisoz Kasap Ozge[6], Falchi Alessandra[18], Favennec Loïc[19], Fernández Guillermo[15], Fiorentino Eleonora[1], Fisa Roser[5], Foglia Manzillo Valentina[20], Fortuna Claudia[21], Ivovic Vladimir[4], Izri Arezki[22], Jiménez Maribel[23,24], Kenan Arserim Suha[25], Kirstein Oscar[26], Kniha Edwin[27], Liénard Emmanuel[10], Lucientes Curdi Javier[15], Mangiapelo Claudia[1], Marsili Giulia[21], Martínez-Barciela Yasmina[28], Martín-Martín Inés[26], Martín-Sánchez Joaquina[17], Mathieu Bruno[29], Mekarnia Nalia[14], Mercier Aurélien[13], Molina Ricardo[23,24], Morales-Yuste Manuel[17], Oguz Gizem[6], Oliva Gaetano[20], Orsini Stefania[1], Ozbel Yusuf[30], Parreira Ricardo[11], Paz Adolfo[31], Pekagirbas Metin[32], Pérez-Cutillas Pedro[9], Platzgummer Katharina[27], Polat Ceylan[33], Polina Alejandro[28], Pomares Christelle[16], Prudhomme Jorian[3], Randrianambinintsoa Fano[14], Risueño José[9], Roca-Geronès Xavier[5], Ruiz-Fons Francisco[13], Sánchez Andrade Rita[31], Scalone Aldo[1], Sevila Julie[18], Sevim Kivanc[6], Toz Seray[30], Yetismis Kardelen[32], Yilmaz Ayda[6], Maia Carla[11], Dvorak Vit[34]

[1]Department of infectious diseases, Vector-borne diseases unit, Istituto Superiore di Sanità, Rome, Italy; [2]Unité des Virus Emergents, Aix Marseille University, IRD 190, INSERM U1207, 13005 Marseille, France; [3]CHU Rennes, Inserm, EHESP IRSET (Institut de Recherche en Santé Environnement et Travail), University of Rennes, France; [4]Department of Biodiversity, FAMNIT, University of Primorska, Koper, Slovenia; [5]Departament de Biologia, Sanitat i Medi Ambient, Facultat de Farmàcia i Ciències de l'Alimentació, Universitat de Barcelona, Barcelona, Spain; [6]Department of Biology, Faculty of Science, Hacettepe University, Ankara, Turkey; [7]UMR MIVEGEC, IRD-CNRS, University of Montpellier, Montpellier, France; [8]Basque Institute for Agricultural Research and Development (NEIKER), Derio, Spain; [9]Department of Animal Health, Faculty of Veterinary Science, University of Murcia, Murcia, Spain; [10]InTheres, University of Toulouse, INRAE, ENVT, Toulouse, France; [11]Instituto de Higiene e Medicina Tropical, University Nova of Lisbon, Lisbon, Portugal; [12]Spanish Game & Wildlife Research Institute (IREC), Ciudad Real, Spain; [13]Inserm U1094, IRD UMR270, Univ. Limoges, CHU Limoges, EpiMaCT - Epidemiology of chronic diseases in tropical zone, Institute of Epidemiology and Tropical Neurology, OmegaHealth, Limoges, France; [14]UR Escape, University of Reims Champagne-Ardenne, Reims, France; [15]Department of Animal Pathology, Faculty of Veterinary Medicine, University of Zaragoza, Spain; [16]Department of Parasitology, CHU Nice, University of Nice Côte d'Azur, Nice, France; [17]Department of Parasitology, Faculty of Pharmacy, University of Granada, Granada, Spain; [18]Department of Emerging Viruses, IRD-INSERM-IRBA, University of Aix-Marseille, University of Corsica, France; [19]University of Rouen Normandy, Rouen, France; [20]Department of Veterinary Medicine and Animal Production, University of Naples Federico II, Naples, Italy; [21]Department of infectious diseases, National reference laboratory for Arboviruses, Istituto Superiore di Sanità, Rome, Italy; [22]Department of Parasitology-Mycology, Avicenne Hospital, Bobigny, France; [23]Medical Entomology Laboratory, National Center for Microbiology, Instituto de Salud Carlos III, Majadahonda, Madrid, Spain; [24]Collaborative Biomedical Research Center in Infectious Diseases (CIBERINFEC), Instituto de Salud Carlos III, Madrid, Spain; [25]Vocational School of Health Sciences, Celal Bayar University, Manisa, Turkey; [26]Medical Entomology and Parasitology, Laboratory, Israeli Ministry of Health, Jerusalem, Israel; [27]Institute of Specific Prophylaxis and Tropical Medicine, Medical University of Vienna, Vienna, Austria; [28]Departamento de Ecoloxía e Bioloxía Animal, Universidade de Vigo, Pontevedra, Spain; [29]Institutes of Bacteriology and Parasitology, Medical Faculty, University of Strasbourg, Strasbourg, France; [30]Department of Parasitology, Faculty of Medicine, Ege University, Izmir, Turkey; [31]Control of Parasites Group (COPAR, GI-2120), Department of Animal Pathology, Faculty of Veterinary, University of Santiago de Compostela, Lugo, Spain; [32]Department of Parasitology, Faculty of Veterinary Medicine, Adnan Menderes University, Aydin, Turkey; [33]Department of Medical Microbiology, Faculty of Medicine, Hacettepe University, Ankara, Turkey; [34]Faculty of Science, Department of Parasitology, Charles University, Prague, Czech Republic

***Corresponding author:** gioia.bongiorno@iss.it

Abstract

Aiming to support policy making and risk assessment of sand fly-borne pathogens through the distribution and prevalence of vector species and the pathogen detection analysis, CLIMOS partners involved in qualitative data collection in 2023 season. Sand flies sampling has been conducted in eleven countries, coupled by environmental (temperature, humidity) and biological (animal presence) variables recording. Collected sand flies were then screened for the presence of sand fly-borne pathogens using standardized detection protocol.
For this reason, prior to pathogen screening start, an external quality assessment (EQA) was performed among participating partners to obtain comparable data on the detection of *Leishmania* and Phlebovirus in surveyed sand flies. Laboratory molecular performance reliability has been tested by following the same protocols outlined in Standards Operating Procedures (SOPs) agreed by all partners, available on Climos website in "Research Plan and Methodology". A Real time PCR assay was performed to detect *Leishmania* by amplifying a kDNA sequence and for phleboviruses detection two Real Time qRT-PCR and one conventional RT-PCR were used.
Established reference laboratories, for phleboviruses and *Leishmania* screening and identification, supplied participating partners with quality-controlled, ready-to-use standard samples, primers and probes. Submitted results have been evaluated and applied protocols optimised based on the reported results.
Canine sera screening for sand fly-borne pathogens presence in same sand flies surveyed sites has been planned to be performed after sand flies' activity season end to detect *Leishmania* prevalence in its reservoir host and screen phleboviruses eventually present in dogs by IFATest and Seroneutralisation assay, respectively.

Keywords: *Leishmania,* Phlebovirus, pathogens detection, sand fly, canine sera

O_{37} Vector competence of European sand flies to *Leishmania* and phleboviruses

Sadlova Jovana[1]*, Bongiorno Gioia[2], Jancarova Magdalena[1], Vojtkova Barbora[1], Hoskova Anna[1], Bernardini Ilaria[2], Bianchi Riccardo[2], Becvar Tomas[1], Stejskalova Marketa[1], Polanska Nikola[1], Volf Petr[1], Dvorak Vit[1]

[1]*Charles University, Prague, Czech Republic.*
[2]*Instituto Superiore di Sanita, Roma, Italy*

***Corresponding author**: sadlovaj@natur.cuni.cz

Abstract

To assess whether European sand fly species support the development of *Leishmania* species newly emerging in Europe and Toscana phlebovirus (TOSV), we tested vector competence in a series of laboratory experiments. Sand flies were infected through the chick skin membrane, *Leishmania* infections were evaluated by light microscopy and qPCR and the representation of morphological forms was assessed from Giemsa-stained gut smears. Sand fly species were considered competent if (i) *Leishmania* survived defecation of blood meal remnants, (ii) colonized the stomodeal valve and (iii) metacyclic stages developed in mature infections. Detection of phleboviruses was performed using TCID 50 and RT-qPCR.
In total*,* more than 40 infection experiments were conducted and more than 2000 sand fly females were dissected and evaluated. We confirmed that *P. perniciosus* and *P. tobbi* are competent vectors of *L. donovani* and *L. major* while they both do not support the development of *L. martiniquensis.* Experiments with field-captured *P. perfiliewi* confirmed its susceptibility to *L. tropica,* parasite loads and the proportion of metacyclic forms increased with time post bloodmeal. *Phlebotomus tobbi* was proved to be susceptible to TOSV B, while *P. perniciosus* colony maintained in Prague was resistant which might be explained by the presence of *Wolbachia* and different midgut microbiome. The vector competence of *S. minuta* could not be tested because females refused to feed blood through membranes; the colonization of *P. mascittii* is ongoing.

Keywords: vector competence, *Phlebotomus*, *Leishmania*, Toscana virus

O38 Development of a sand fly semio-chemical attractant remote monitoring device

Courtenay Orin[1*], Chelbi Ifhem[2], Hernandez-Colina Arturo[1], Hamilton Gordon[2], Gligorić Nenad[3], Urošević Vladimir[3], Berriatua Eduardo[4], Risueño José[4], Bongiorno Gioia[5], Oliva Gaetano[6], Ozbel Yusuf[7], Seray Toz[7], Maia Carla[8]

[1]Zeeman Institute and School of Life Sciences, University of Warwick, UK
[2]Faculty of Health and Medicine, University of Lancaster, UK
[3]Zentrix Lab, Pančevo, Serbia
[4]University of Murcia, Murcia, Spain
[5]Scegli l'Istituto Superiore di Sanità, Rome, Italy
[6]University of Naples Federico II, Naples, Italy
[7]EGE University, Izmir, Turkey
[8]University NOVA of Lisbon, Lisbon, Portugal

***Corresponding author:** orin.courtenay@warwick.ac.uk

Abstract
In pursuit of a technologically simple, affordable and sensitive trap to detect key sand fly vectors of *Leishmania infantum* and/or *Phleboviruses* in sentinel sites, the CLIMOS project is approaching this goal in three main steps: (i) to identify candidate semio-chemicals that are attractive to multiple vectors; (ii) to field test the chemicals' attractiveness when deployed in controlled-release dispensers in multiple endemic regions; and (iii) to incorporate the loaded dispensers alongside sticky papers in a configuration tailored specifically for optimal trapping of sand flies, into a novel modular developed trap device with incorporated remote monitoring.
We will test chemicals identified in the literature as potential attractants to *P. perniciosus*, *P. papatasi*, and *Lu. longipalpis* sand fly populations in laboratory "choice" experiments using a Y-tube olfactometer. Candidate compounds identified in the laboratory will be formulated in controlled release dispensers, tested in the laboratory, and then evaluated under field conditions in Spain, Italy, Turkey and Portugal. Semio-chemical dispensers will be co-located with CDC light traps (no-light) in replicated controlled experiments. Alongside, a prototype monitoring device will be designed to incorporate the chemical dispensers with a widely available high-quality camera, LED lighting, and IoT modules to automatically remotely count sand flies captured on sticky surfaces, using optical recognition on the edge.
The work contributes to the CLIMOS objective of developing an Early Warning System against *Leishmania* and Phlebovirus transmission.

Key words: CLIMOS, sand flies, EWS, semio-chemicals, traps, surveillance, lures.

O39 Development of recombinant salivary antigens as risk markers

Kolářová Iva[1]*, Arserim K. Suha[2], Pekagirbas Metin[3], Yetismis Kardelen[4], Berberoglu Umut[5], Altug Unal[5], Dvořák Vít[1], Jelínková Kristýna[1], Özbel Yusuf[4], Töz Seray[4], Volf Petr[1], Volfová Věra[1], Maia Carla[6]

[1]Department of Parasitology, Faculty of Science, Charles University, Prague, Czech Republic
[2]Celal Bayar University, Vocational School of Health Sciences, Manisa, Turkey
[3]Aydin Adnan Menderes University, Veterinary School, Department of Parasitology, Aydin, Turkey
[4]Department of Parasitology, Faculty of Medicine, Ege University, Izmir, Turkey
[5]Turkish Ministry of Health, Ankara, Turkey
[6]University Nova of Lisbon, Lisbon, Portugal

***Corresponding author**: iva.kolarova@natur.cuni.cz

Abstract

Host antibody levels to sand fly salivary proteins reflect the intensity of host exposure to sand fly bites and can be used as a marker of exposure. To standardise the assay, recombinant sand fly salivary antigens provide a more reproducible option over the lysate from dissected salivary glands. In the CLIMOS project, the recombinant antigen-based ELISA assay is used to measure exposure to vector bites in sentinel dog populations as an early warning surveillance tool for circulating Leishmania infection and to evaluate the efficacy of developed protective measures. Three sand fly vector species are in the focus of the CLIMOS project: Phlebotomus perniciosus and P. tobbi as vectors of Leishmania infantum and P. papatasi as vector of L. major. Phlebotomus perniciosus rSP03B (yellow-related protein) has already been identified, validated, and expressed and will be used in the CLIMOS project to screen dog populations in Portugal, Spain, and Italy. New recombinant antigens are being developed for P. papatasi and P. tobbi. Sera from dogs bitten by P. papatasi or P. tobbi were collected in endemic areas in Turkey where these sand fly species are abundant. Positive sera have been analysed by immunoblot with salivary gland lysates and used to identify antigenic proteins by immunoprecipitation assay followed by mass spectrometry. Selected proteins have been expressed in E. coli and will be tested and validated as risk markers of sand fly exposure.

Keywords: Recombinant salivary proteins, risk markers, *Phlebotomus perniciosus, Phlebotomus papatasi, Phlebotomus tobbi*

Funding: The CLIMOS consortium is co-funded by the European Commission grant 101057690 and UKRI grants 10038150 and 10039289. The six Horizon Europe projects, BlueAdapt, CATALYSE, CLIMOS, HIGH Horizons, IDAlert, and TRIGGER, form the Climate Change and Health Cluster.

O40 Large-scale analysis of long-term historical data of Sand-Fly's population

Waitz Yoni[1]*, Blesic Suzana[2], Natal Sergio[3], Soheili Majid[4], Wang Danyang[5], Paz Shlomit[1]

[1]*University of Haifa, Haifa, Israel.*
[2]*Institute for Medical Research, University of Belgrade, Belgrade, Serbia.*
[3]*Predictia, Santander, Spain.*
[4]*Helmholtz Centre for Environmental Research, Leipzig, Germany.*
[5]*Wageningen University, Wageningen, Netherlands.*

***Corresponding author:** yonyonw@gmail.com

Abstract

Globally, the changing climate and environment are rapidly altering both the spatial and temporal patterns of transmission of sand-fly borne diseases. These changes influence insect population dynamics and their contact rates with host-vector-pathogen.
CLIMOS-Project (Climate Monitoring and Decision Support Framework for Sand Fly-borne Diseases Detection and Mitigation) is a continental initiative that aims to conduct innovative and applied platform for a better preparedness for current and future threat of Sand Fly-Borne Diseases (SFBDs) that is expected to be intensified by climate and environmental changes. Based on historical databases of VectorNet and EDENext projects, a large-scale analysis of long-term data of Phlebotomine sand flies obtained from decades of field collections. Historical data investigation was detected as a valuable tool for the assessment of population dynamics and how they are shaped by long- and short-term climatic variability, environmental properties, and human activities. The challenges of unifying data from different sources will be described. Preliminary insights on the temporal and spatial patterns of some common phlebotomine species in Europe and in the Middle East will be reported. These insights will be a cornerstone for predicting the occurrences of SFBDs, serving as an early warning system.

Keywords: Environmental modelling, population, seasonal activity, climate change.

The data for EDENext was obtained from the Palebludata website (https://www.palebludata.com). Additional details or publications associated with the dataset can be found at the provided website. The data for Vectornet was obtained from the ECDC. Additional details or publications associated with the dataset can be found at the provided website - https://www.ecdc.europa.eu/en.

O41 Originality, simplicity and reusability of visual tools developed for CLIMOS EWS

Gligorijević Vladan[1]*

[1]*CubexLab B.V, Amsterdam, Netherlands.*

***Corresponding author:** vladan@cubexlab.eu

Abstract

The complexity and volume of data collected and processed throughout the life of the CLIMOS project present a unique challenge for digital and visual interpretation when it comes to presenting this data at a level understandable for the end users. Existing tools in this domain share a similar design, data manipulation tools, and user experience. The challenge for the CLIMOS project is to create a state-of-the-art system that future systems could use as a reference during the transition to more modern tools for a better user experience.
Taking this into account, CubexLab's approach was to develop modular tools for displaying data that will be presented on the CLIMOS EWS. For the design of the CLIMOS EWS, we did not follow previously known platforms; instead, we took a step and a half forward to design a platform that will remain ahead of its time even after the project ends, achieving a state-of-the-art status. We applied a slightly futuristic style to already-known tools such as diagrams, charts, time series, and forecasting. To provide end users with a sense of simplicity and comfort when manipulating the tools found on the CLIMOS EWS, we proactively created the platform to cater to users with minimal requirements—those who seek a simple yes or no answer regarding sand fly activity—while also accommodating users with high criteria and professional purposes. Visual tools built as modular blocks can be reused to create new EWS, monitoring platforms, health platforms, and more. Additionally, CubexLab followed all modern web standards and considered the applicability of these visual tools to the most vulnerable user groups, who, due to developmental limitations, have previously been unable to use similar tools or could use them only in a limited capacity. The CLIMOS EWS will provide the capability of using its tools to people with special needs.

Keywords: Early warning system, futuristic visualization, simplicity, accessibility, modern web

The CLIMOS consortium is/was co-funded by the European Commission grant 101057690 and UKRI grants 10038150 and 10039289.
The six Horizon Europe projects, BlueAdapt, CATALYSE, CLIMOS, HIGH Horizons, IDAlert, and TRIGGER, form the Climate Change and Health Cluster.

O42 The development of an Early Warning System under the framework of CLIMOS project

Blesic Suzana[3], Natal Sergio[1]*, Paz Shlomit[2], San Martín Daniel[1], Soheili Majid[4], Wang Danyang[5], Waitz Yoni[2]

[1]Predictia Intelligent Data Solutions, Santander, Spain
[2]University of Haifa, Haifa, Israel
[3]Institute for Medical Research, University of Belgrade, Serbia
[4]Helmholtz Centre for Environmental Research, Leipzig, Germany
[5]Wageningen University, Wageningen, Netherlands

***Corresponding author:** natals@predictia.es

Abstract

Sand fly-borne diseases present a significant public health threat, worsened by the dynamic interplay of climate change, environmental factors, and human activities. The CLIMOS project (Climate Monitoring and Decision Support Framework for Sand Fly-borne Diseases Detection and Mitigation) addresses this challenge by establishing an innovative Early Warning System (EWS) tailored to monitoring sand fly activity. CLIMOS integrates hazard monitoring, forecasting, risk assessment, communication, and preparedness activities. Through large-scale analysis of historical sand fly data, the project reveals population dynamics influenced by climatic variations and human impacts. By unifying data from diverse sources, CLIMOS lays the groundwork for predicting the temporal and spatial patterns of sand fly-borne diseases in Europe and the Middle East. The implementation of this EWS involves several key components: employing modeling techniques based on niche models and machine learning models to predict the occurrence of sand flies, and establishing a robust communication network to disseminate warnings and guidance to public health authorities and the general public. Additionally, the system will address various prediction horizons, encompassing short-range forecasts, seasonal forecasts, and climate projections. By leveraging state-of-the-art technology and interdisciplinary collaboration, the system enhances the capacity for early detection and response to disease threats. These insights form the cornerstone of an EWS, empowering stakeholders to undertake timely interventions and mitigate the impact of emerging health threats.

Keywords: Early warning system, forecast, niche modeling, machine learning, sand fly.

The data for EDENext was obtained from the Palebludata website (https://www.palebludata.com). Additional details or publications associated with the dataset can be found at the provided website.
The data for Vectornet was obtained from the ECDC. Additional details or publications associated with the dataset can be found at the provided website.

O43 Promoting and disseminating sand fly research to various audiences

Maia Carla[1], Altuğ Ünal[2], Athanatos Manos[3], Berriatua Eduardo[4], Blesić Suzana[5], Bongiorno Gioia[6], Charrel Remi[7], Courtenay Orin[8], Davidovich-Cohen Maya[9], Depaquit Jerome[10], Dvorak Vit[11], Erisoz Ozge[12], Ferraro Federica[13], Fleming Padraic[14], Foglia-Manzillo Valentina[15], Gligorić Nenad[16], Gligorijević Vladan[17], Ferreira Maia Maria Joao[18], Hamilton Gordon[19], Hempelmann Nils[20], Hatzakis Tally[14], Ivovic Vladimir[21], Kirstein Oscar[9], Kniha Edwin[22], Kolarova Iva[11], Ozbel Yusuf[23], Paz Shlomit[24], Robert-Gangneux Florence[25], Sadlova Jovana[11], Samaniego Luis[26], San Martin Daniel[27], Van Langevelde Frank[28], Volf Petr[11], Wright David[14], Tuñon Sandra[29], Guardado Diana[29]*

[1]University NOVA of Lisbon, Lisbon, Portugal, [2]Turkish Ministry of Health, Ankara, Turkey; [3]Telecommunications Systems Institute, Chania, Greece; [4]University of Murcia, Murcia, Spain; [5]Institute for Medical Research, University of Belgrade, Belgrade, Serbia; [6]Istituto Superiore di Sanità, Rome, Italy; [7]Aix-Marseille University, Marseille, France; [8]University of Warwick, Coventry, UK; [9]Israeli Ministry of Health, Jerusalem, Israel; [10]University of Reims Champagne-Ardenne, Reims, France; [11]Charles University, Prague, Czech Republic; [12]Hacettepe University, Ankara, Turkey; [13]Ministry of Health, Italy, [14]Trilateral Research, Waterford, Ireland; [15]University of Naples Federico II, Naples, Italy; [16]Zentrix Lab, Pancevo, Serbia; [17]CubexLab, Amsterdam, Netherlands; [18]Karlsruhe Institute of Technology, Karlsruhe, Germany; [19]Lancaster University, Lancaster, UK; [20]Open Geospatial Consortium, Leuven, Belgium; [21]University of Primorska, Koper, Slovenia; [22]Medical University of Vienna, Vienna, Austria; [23]Ege University, Izmir, Turkey; [24]University of Haifa, Haifa, Israel; [25]University of Rennes, Rennes, France; [26]Helmholtz Centre for Environmental Research, Leipzig, German; [27]Predictia, Santander, Spain; [28]Wageningen University, Wageningen, Netherlands; [29]F6S Network Ireland Limited, Dublin, Ireland

***Corresponding author:** dianag@f6s.com

Abstract

CLIMOS project is generating science-based predictions, actions, and policy-relevant recommendations to mitigate the climate change-induced emergence and spread of sand fly-borne diseases. The project focuses on developing precision vector and infection surveillance methods and networks. It also promotes collaboration among research institutions, public authorities, companies, civil society, and the natural environment, recognizing the interconnectedness of human, animal, and environmental health (One Health concept). Through these efforts, CLIMOS is addressing common solutions to national challenges related to sand fly-borne diseases.

Effective communication and dissemination of the CLIMOS project's concepts, outcomes, and benefits are essential to ensure widespread awareness and adoption of the project's results and to leverage One Health literacy among our target audience. Additionally, increasing awareness of the project and its potential advantages will open new commercial and scientific opportunities beyond the project's duration. Recognizing the variety of project stakeholders, the partners are planning activities to involve all relevant parties and communities. Our goal is to foster a shift in mindset, encouraging, as well, citizens to recognize and understand the link between climate change and public health impacts.

Communication activities will be implemented during the project lifetime and underscore the importance of vigilance against harmful vector-borne diseases and support for measures to combat them. How can we effectively promote and disseminate scientific knowledge to diverse audiences? This session will showcase CLIMOS's efforts to promote and disseminate science to diverse

audiences, including tailored social media campaigns on Facebook, LinkedIn, Twitter, and Instagram, recording podcasts, and designing factsheets, among others.

Keywords: sand fly, dissemination, communicating science, stakeholder engagement

O44 IgM promotes genetic exchange of *Leishmania* inside the sandfly vector

Serafim Tiago D.[1]*, Iniguez Eva[1], Barletta Ana Beatriz F.[2], Cecilio Pedro[3], Doehl Johannes S.P.[1], Short Mara[1], Lack Justin[4], Nair Vinod[5], Disotuar Maria[1], Wilson Timothy[1], Coutinho-Abreu Iliano V.[1], Meneses Claudio[1], Andersen John[4], Alves e Silva Thiago Luiz[6], Oliveira Fabiano[1], Vega-Rodriguez Joel[6], Barillas-Mury Carolina[2], Ribeiro José M.C.[4], Beverley Stephen M.[7], Kamhawi Shaden[1], Valenzuela Jesus G.[1]*

[1]*Vector Molecular Biology Section, Laboratory of Malaria and Vector Research, National Institute of Allergy and Infectious Diseases, National Institutes of Health, Rockville, MD, USA*
[2]*Mosquito Immunity and Vector Competence Section, Laboratory of Malaria and Vector Research, National Institute of Allergy and Infectious Diseases, National Institutes of Health, Rockville, MD, USA*
[3]*Vector Biology Section, Laboratory of Malaria and Vector Research, National Institute of Allergy and Infectious Diseases, National Institutes of Health, Rockville, MD, USA*
[4]*NIAID Collaborative Bioinformatics Resource, National Institute of Allergy and Infectious Diseases, National Institutes of Health, Bethesda, MD, USA*
[5]*Rocky Mountain Laboratories, National Institute of Allergy and Infectious Diseases, National Institutes of Health, Hamilton, MT, USA*
[6]*Molecular Parasitology and Entomology Unit, Laboratory of Malaria and Vector Research, National Institute of Allergy and Infectious Diseases, National Institutes of Health, Rockville, MD, USA*
[7]*Department of Molecular Microbiology, School of Medicine, Washington University, St. Louis, MO, USA*

***Corresponding authors:** jvalenzuela@niaid.nih.gov; tiago.donatelliserafim@nih.gov

Abstract

Host factors facilitating *Leishmania* genetic exchange are not well understood. Here, we demonstrate that natural IgM antibodies, but not IgG or IgA, facilitate parasite genetic exchange. IgM induces the gradual and transient formation of a structured *Leishmania* clump that releases viable parasites in a process essential for *L. major* hybridization *in vitro*. Parasite hybrids and 3-nucleated parasites were observed inside this structure, named the *Leishmania* mating clump. IgM was also required for or significantly increased *Leishmania* hybrid formation *in vivo*. At minimum, we observed a 12-fold increase in the proportion of hybrids recovered from sand flies provided a second blood meal containing IgM compared to controls. Genetic backcross events in sand flies were only observed in the presence of IgM, and were reproducibly recovered, reinforcing the relevance of natural IgM for *Leishmania* genetic exchange in vivo. All *Leishmania* crosses reported here resulted in full genome hybrids with comparable recombination structures that arose from the fusion of the two parental lines. *Leishmania* co-option of a host antibody to facilitate mating in the insect vector establishes a new paradigm of parasite-host-vector coevolution that promotes parasite diversity and fitness through genetic exchange.

Keywords: Sandfly, Leishmania, IgM, genetic exchange

O45 Salivary glands of *Nyssomyia neivai* during *in vivo* infection by different Amazonian *Leishmania* (*Viannia*) species

Soares Rodrigo Pedro Pinto[1,2]*, Fontes Igor[1], Dutra-Rêgo Felipe[1], Matta Vania L.[2], Araujo Flores Gabriela[2], Sandoval Carmen[2], Andrade Andrey J.[3], Costa-Ribeiro Maria C.[3], Shaw Jeffrey[4], Laurenti Márcia[2]

[1]*Grupo Biotecnologia Aplicada ao Estudo de Patógenos (BAP), Instituto René Rachou, Fundação Oswaldo Cruz (FIOCRUZ), Belo Horizonte, MG, Brazil*
[2]*Laboratório de Patologia das Moléstias Infecciosas, Departamento de Patologia, Faculdade de Medicina, Universidade de São Paulo (USP), São Paulo, SP, Brazil*
[3]*Laboratório de Parasitologia Molecular, Departamento de Patologia Básica, Setor de Ciências Biológicas, Universidade Federal do Paraná (UFPR), Curitiba, PR, Brazil*
[4]*Departamento de Parasitologia, Instituto de Ciências Biomédicas, Universidade de São Paulo (USP), São Paulo, SP, Brazil*

***Corresponding author:** rodrigosoares28@hotmail.com

Abstract

Nyssomyia neivai is a suspected vector of transmitting *L. braziliensis* in the South of Brazil. Sand flies were captured in Castro municipality (24º59'31.2" S, 49º37'40.8" W), Paraná state, Brazil. Salivary glands extracts (SGEs) are important modulators of infection. We chose this species´ SGEs glands for hamster infection with six *L. (Viannia)* including *L. braziliensis* (MHOM/BR/2001/BA788), *L. guyanensis* (MHOM/BR/85/M9945), *L. shawi* (MHOM/BR/96/M15789), *L. lindenbergi* (MHOM/BR/98/M15733) and *L. naiffi* (MDAS/BR/79/M5533). Information on suitable animal models to understand immunopathology are scarce. We explored the use of the golden hamster *Mesocricetus auratus* as an infection model. Parasites were injected into the footpad and followed up at 20- and 40-days PI. Parasites were mixed with SGEs from the sandfly prior to infections. Animals were euthanized for histopathological evaluation of the footpads, spleen, and liver. The parasite burden was evaluated in the skin and draining lymph nodes. All species, except *L. guyanensis*, failed to generate evident macroscopic lesions 40 days PI. The *L. guyanensis* lesions were swollen but did not ulcerate and microscopically were characterized by an intense inflammatory exudate. Despite the fact the other species did not produce visible skin lesions there was no or mild pro-inflammatory infiltration at the inoculation site and parasites survived in the hamster skin/lymph nodes and even visceralized. In conclusion, hamster was a suitable model for all species, except for *L. naiffi*. This work was supported by CNPq (305238/2023-0) and FAPEMIG (APQ-04991-28).

Keywords: *Nyssomyia neivai*, *Leishmania* (*Viannia*) species, host-parasite interaction, *Mesocricetus auratus*

O46 Azadirachtin disrupts ecdysone signaling and alters *Phlebotomus perniciosus* immunity

Vieira Stahl Cecilia[1]*, Bisogno Sara[2], Salvemini Marco[2], Telleria L. Erich[1], Volf Petr[1]

[1]*Department of Parasitology, Faculty of Science, Charles University, Czech Republic*
[2]*Department of Biology, University of Naples Federico II, Italy.*

***Corresponding author**: ceciliastahl@gmail.com

Abstract

Phlebotomus perniciosus is the natural vector of *Leishmania infantum* (the causative agent of visceral leishmaniasis) in the western region of the Mediterranean basin of Europe. The *Leishmania* cycle occurs in the sand fly midgut, where parasites resist the activation of sand fly immune responses mainly regulated by the IMD pathway. Recently, it was demonstrated that insect immunity is under hormonal regulation. Moreover, ecdysone's role in sand fly immunity has never been studied. Here, we manipulated the neuroendocrine system of *P. perniciosus* larvae and females by adding Azadirachtin (AZA, a triterpenoid from the *Azadirachta indica* tree that affects ecdysone synthesis) to their food, compared to the addition of ecdysone or AZA plus ecdysone. We measured the effects on mortality and ecdysis. We also assessed the expression of genes related to ecdysone signaling and immunity, such as antimicrobial peptides (AMPs), Ecdysone receptor (EcR), and the ecdysone-induced genes *Serpent*, *Eip74F*, and *Eip75B* by quantitative PCR. In larvae, AZA significantly inhibited the molting process and the gene expression of ecdysone-induced genes and three AMPs. Interestingly, adding ecdysone to the larval food reversed gene downregulation and molting inhibition. In females fed on sugar meals, AZA also suppressed ecdysone signaling-related genes, and two of the three AMPs analyzed were restored by simultaneous ecdysone treatment. Additionally, the ecdysone alone supplemented into the female sugar meal increased *EcR*, *Serpent*, and *Attacin* gene expression levels. These results highlight the role of ecdysone in regulating sand fly immunity, which potentially could interfere with *Leishmania* infection.

Keywords: *Phlebotomus perniciosus*, ecdysone, immunity, antimicrobial peptides

O47 Surface molecules from *Leishmania* and bacteria increase the expression of sand fly genes coding for antimicrobial peptides and gut surface proteins

Telleria L. Erich[1]*, Vomáčková-Kykalová Barbora[1], Sassù Fabiana[1], Dutra-Rêgo Felipe[2], Volf Petr[1], Soares P. Rodrigo[2]

[1]*Charles University, Faculty of Science, Department of Parasitology, Viničná 7, 128 44, Prague, Czech Republic*
[2]*Biotechnology Applied to Pathogens (BAP), Instituto René Rachou, Fundação Oswaldo Cruz (Fiocruz), Av. Augusto de Lima, 1715, CEP: 30190-009, Belo Horizonte, MG, Brazil*

***Corresponding author:** erich.loza.telleria@natur.cuni.cz

Abstract

Our research is focused on the complex interaction between sand flies and pathogens. More specifically, we are searching for sand fly genes that have a key role in this interplay. *Leishmania* lipophosphoglycans (LPG) play important roles in the parasite life cycle in sand flies and vertebrate hosts. Similarly, bacteria produce lipopolysaccharides (LPS) that are recognized as virulence factors. These microbe surface molecules are pathogen-associated molecular patterns (PAMPs) that trigger the regulatory mechanisms of immune response in the vertebrate hosts. Sand flies have an active immune response, and we investigated whether these specific PAMPs modulate the expression of sand fly genes involved in midgut coating and immune response. *Lutzomyia longipalpis* and *Phlebotomus papatasi* were fed on blood containing purified LPG from *Leishmania infantum* and *Leishmania major* and LPS from *Escherichia coli*. Using quantitative PCR, we assessed the expression of a set of specific genes. *Leishmania infantum* LPG and *E. coli* LPS increased the expression of a mucin-like protein in *L. longipalpis*. *Leishmania major* LPG increased the expression of a galectin in *L. longipalpis*, while *E. coli* LPS did the same in *P. papatasi*. Moreover, both types of *Leishmania* LPG increased the activity of antimicrobial peptide attacin and defensin genes in both sand fly species, and *E. coli* LPS had a similar effect in *L. longipalpis*. Our study shows that these PAMPs increase the expression of genes that help maintain a healthy gut and control infections, revealing new aspects of the complex balance between sand flies and microbes.

Keywords: PAMPs; *Lutzomyia*; *Phlebotomus*; gut protein; innate immunity

O48 Uncovering the secrets of vector competence focusing on two Amazonian sand fly populations from leishmaniasis endemic and non-endemic areas

Marialva dos Santos Eric F.[1], Ramos de Almeida Victor[2], Spelta Guilherme[2], Pessoa Felipe[1], Rios Velasquez Claudia[1], Batista Michel[3], Marchini Fabricio[3], Tempone Antonio J.[2], Traub-Cseko Yara M.[2]*

[1]Lab. Ecologia de Doenças Transmissíveis na Amazônia, Instituto Leônidas e Maria Deane, Fiocruz Amazônia, Brazil
[2]Lab. Biologia Molecular de Parasitas e Vetores, Instituto Oswaldo Cruz, Fiocruz
[3]Lab. de Ciências e Tecnologias Aplicadas em Saúde, Instituto Carlos Chagas-Fiocruz, PR

***Corresponding author**: ytraub@ioc.fiocruz.br

Abstract

Nyssomyia umbratilis in the northern region of the Negro river (Manacapuru, MA), state of Amazon, Brazil, is found naturally infected with *Leishmania guyanensis*, while in the southern region of the river (Rio Preto da Eva, RPE) this does not occur, with the river apparently being a geographic barrier for the transmission of *L. guyanensis*. Previous comparative studies found some biological and genetic differences between the two populations, and i*n vitro* adhesion studies of parasites with dissected intestines from the vectors, found lower adhesion in sandflies collected in Manacapuru. Since these insects are not colonized we are using molecular approaches for the identification of possible mechanisms for the susceptibility difference. Since the *Leishmania* cycle occurs solely within the vector gut, we performed a comparative proteomic analysis of insect guts. A few proteins were unique and many had a significantly different abundance in both populations. Among these there were proteins involved with gut acidification and motility, and immunity. Promising candidates were validated through transcription experiments and some are being silenced in the model vector *Lutzomyia longipalpis*, for infectivity tests. We also investigated a possible involvement of the microbiota in the susceptibility differences, through metagenomic. Manacapuru has a greater bacterial diversity in relation to Rio Preto da Eva, the genera Rickettsia, Cryocola, Prevotella, Porphyromonas and Caulobacter being the most prevalent. We have also performed gut transcriptomics and these data are presently under analysis.

Keywords: vector-parasite interaction, vector competence, sandfly gut proteomics, sandfly microbiota metagenomics.

O49 Infectiousness of natural hosts of *Leishmania major* to sand flies at micro- and macro-scale

Vojtkova Barbora[1]*, Becvar Tomas[1], Mekarnia Nalia[2], Frynta Daniel[3], Benallal Kamal Eddine[1,4], Harrat Zoubir[5], Volf Petr[1], Sadlova Jovana[1]

[1]*Department of Parasitology, Faculty of Science, Charles University, Prague, Czech Republic*
[2]*UR 7510 ESCAPE - USC Anses, School of Pharmacy, Université de Reims Champagne-Ardenne, 10 Pôle Santé, 51100 Reims, France*
[3]*Department of Zoology, Faculty of Science, Charles University, Prague, Czech Republic*
[4]*Laboratory of Arboviruses and Emergent viruses, Institut Pasteur of Algiers, Algiers, Algeria*
[5]*Algerian Academy of Science and Technology, Algeria*

***Corresponding author:** vojtkob@natur.cuni.cz

Abstract

Cutaneous leishmaniasis caused by *L. major* is a typical zoonosis transmitted by sand flies and maintained in rodent reservoirs. Female sand flies were assumed to become infected by feeding on a host skin lesion and the relative contribution of asymptomatic individuals to disease transmission was unknown. Another important but poorly understood factors affecting transmission are the parasite distribution in the host body and the number of skin amastigotes needed to establish sand fly infection. We compared transmissibility in symptomatic and asymptomatic individuals of *Meriones shawi*, a North African reservoir, and correlated the spatial distribution of *L. major* with infectiousness to sand flies at the microscale. Rodents were infected with a natural dose of *L. major* derived from the guts of infected sand flies. Animals were subjected to xenodiagnoses with *Phlebotomus papatasi* and the infectiousness on the microscale was evaluated using microbiopsies. Analyses of 113 xenodiagnostic trials confirmed that asymptomatic and presymptomatic animals were fully comparable source of infection to symptomatic animals. *Leishmania* parasites were mainly localized in the skin in *M. shawi* and their distribution in infected ears was heterogeneous with marked differences between the centre of the lesion, the margin of the lesion and the outer zones. The margins of the lesions were the best source of infection, and even less than 10 amastigotes were sufficient to produce a heavy infection in the vector. These results may help to fill the gaps in understanding *Leishmania* transmission and enable precise modelling of the epidemiology of cutaneous leishmaniasis.

Keywords: *Meriones shawi; Leishmania major; Phlebotomus papatasi*, asymptomatic infection; reservoir host

O50 Thoughts about the very complicated taxonomy of South-East Asian sandflies

Depaquit Jérôme[1]*, Randrianambinintsoa José Fano[1], Rahola Nil[2], Bañuls Anne-Laure[2], Prasetyo Didot B.[3], Boyer Sébastien[3], Vongphayloth Khamsing[4]

[1]Université de Reims Champagne-Ardenne, Faculté de Pharmacie, UR EpidémioSurveillance et Circulation de Parasites dans les Environnements (ESCAPE), and ANSES, USC Pathogènes-Environnement - Toxoplasme-Arthropodes- Réservoirs- bioDiversité (PETARD), Reims, France; Centre Hospitalo-Universitaire, Laboratoire de Parasitologie-Mycologie, 51092 Reims, France
[2]UMR MIVEGEC, University of Montpellier-IRD-CNRS, Montpellier, France
[3]Medical and Veterinary Entomology Unit, Institut Pasteur du Cambodge, 983, Phnom Penh, Cambodia
[4]Laboratory of Vector-Borne Diseases, Institut Pasteur du Laos, Samsenhai Road, Ban Kao-Gnot, Sisattanak District, 3560 Vientiane, Lao PDR

***Corresponding author:** jerome.depaquit@univ-reims.fr

Abstract

Southeast Asia has long been considered a leishmaniasis-free area, leading to limited studies on the usual *Leishmania* vectors. Historically, three main entomologists have significantly contributed to the knowledge of the sandfly fauna in this region: Jean Raynal in the 1930s, Lawrence Quate in the 1960s and Donald J. Lewis in the 1970s. More or less successfully, and quite paradoxically, these authors have either related SE Asian specimens to species described from India or even China, or have described new species, or have proposed more or less relevant synonymizations. Sometimes, aberrant information appears in the literature and is perpetuated, in the absence of criticism, publication after publication, transforming the error as truth! We can cite the example of *Se. iyengari* whose the male was described from southwest India and the female from Vietnam, despite the obvious heterogeneity between these populations.
The scarcity of specialists in SE Asia has further contributed to approximations and taxonomic errors. This communication aims to present the latest advances in the taxonomy of sandflies in this region. It will highlight the main pitfalls and limitations of species-level identification and propose a consensual morphological and molecular database, that will enable less experienced entomologists to be able to identify sandflies with greater certainty.

Keywords: Systematics, DNA barcoding, *Idiophlebotomus*, *Chinius*, *Phlebotomus*, *Sergentomyia*, *Grassomyia*, phylogeny

O_{51} Hidden in plain sight: discovery of phlebotomine sandflies in Singapore and description of four species new to science

Ding Huicong[1#], Torno Majhalia[1#], Vongphayloth Khamsing[2,3], Ng Germaine[1], Tan Denise[1], Sng Wendy[4], Lim Kelvin[4], Randrianambinintsoa José Fano[2], Depaquit Jérôme[2,5], Tan Cheong Huat[1]*

[1]*Environmental Health Institute, National Environment Agency, Singapore*
[2]*Université de Reims Champagne-Ardenne, France*
[3]*Laboratory of Vector-Borne Diseases/Zoology, Institut Pasteur du Laos, Lao PDR*
[4]*Animal & Veterinary Service, National Parks Board, Singapore*
[5]*Laboratoire de Parasitologie-Mycologie, Centre Hospitalo-Universitaire, France*
[#]*HD and MT are joint first authors*

***Corresponding author**: tan_cheong_huat@nea.gov.sg

Abstract

Phlebotomine sand flies (Diptera: Psychodidae) are small, blood-sucking insects that are of significant public and veterinary health importance for their role in the transmission of *Leishmania* parasites and arboviruses. Despite presence of sandflies in the Southeast Asian region, there is no published record of their presence in Singapore. Sandflies were collected from routine vector surveillance activities carried out by the National Environment Agency. An integrated taxonomic workflow, involving morphological review and DNA barcoding of the mitochondrial *cytochrome b* (*cytb*) and *cytochrome c oxidase subunit I* (*COI*) gene, was applied for species identification. We report the discovery of eight species of phlebotomine sandflies from Singapore, including one *Phlebotomus* species and three *Sergentomyia* species new to science. Phylogenetic analyses of the *cytb* gene suggest that the new *Phlebotomus* species, belonging to subgenus *Euphlebotomus*, is closely related to *Phlebotomus argentipes*, an important vector of *Leishmania donovani* from the South Asian region. The detection of sandflies in Singapore underscores the importance of continuous monitoring and surveillance. Data presented here will provide greater understanding of sandfly species diversity and distribution, aiding in the identification of high-risk areas and contribute to the development of an early warning system. This is particularly critical in the light of the recent canine leishmaniasis detection in the country.

Keywords: Southeastern Asia, *Phlebotomus*, *Sergentomyia*, new species, systematics

O52 Suspected novel species of Phlebotomine sand flies (Diptera: Psychodidae) from Uthai Thani Province, Thailand, and its potential role in *Trypanosoma* sp. transmission

Pataradool Thanapat[1,2,3], Sunantaraporn Sakone[2], Yuan Jin-Min[3], Dvorak Vit[4], Volf Petr[4], Siriyasatien Padet[2,3]*

[1]*Medical Sciences Program, Faculty of Medicine, Chulalongkorn University, Bangkok, Thailand*
[2]*Center of Excellence in Vector Biology and Vector-Borne Diseases, Faculty of Medicine, Chulalongkorn University, Bangkok, Thailand*
[3]*Department of Parasitology, Faculty of Medicine, Chulalongkorn University, Bangkok, Thailand*
[4]*Department of Parasitology, Faculty of Science, Charles University, Prague, Czech Republic*

***Corresponding author:** padet.s@chula.ac.th

Abstract

Phlebotomine sand flies (Diptera: Psychodidae) are known vectors of several medically and veterinary important pathogens such as *Leishmania* and *Bartonella*. These insects inhabit cool, dark, and humid environments and are often found in caves. In this study, we collected sand flies from Pu Wai Cave, a tourist destination in Uthai Thani province, Thailand. A total of 154 female and 16 male sand flies were captured. Using morphological characteristics and genetic analysis based on cytochrome c oxidase subunit I (*COI*) and cytochrome b (*cytB*), four species of sand flies were identified: *Idiophlebotomus* sp. (66.9%), *Phlebotomus barguesae* (16.9%), *Sergentomyia anodontis* (14.9%), and *Ph. stantoni* (1.3%). The *Idiophlebotomus* sp. specimens resembled *Id. teshi* morphologically, but the males had longer genital filaments and styles. Additionally, their *COI* gene sequence showed only 93.4-95% similarity to *Id. teshi*. Phylogenetic analysis placed our *Idiophlebotomus* sp. in a separate branch from *Id. teshi*. Subsequently, all female samples were screened with PCR assays targeting the *gltA* gene for *Bartonella* and the *SSU* rRNA gene for *Trypanosoma*. The molecular screening showed 25 females (24 of *Idiophlebotomus* sp. and 1 of *Se. anodontis*) tested positive for *Trypanosoma* DNA, all identified as *Trypanosoma noyesi*, while none contained *Bartonella* DNA. These findings suggest our *Idiophlebotomus* sp. represents a potentially novel species and a candidate vector of *Trypanosoma* sp. Further investigations are needed to fulfill Koch's postulates and definitively incriminate its vector competence.

Keywords: Sand flies, *Trypanosoma* sp., *Bartonella* sp., novel species, Thailand

O53 Analysis of phlebotomine sandflies (Diptera: Psychodidae) in Laos from 2012-2024 identifies new species and diverse taxa

Vongphayloth Khamsing[1,2]*, Randrianambinintsoa José Fano[2], Lakeomany Khaithong[1], Phommavanh Nothasine[1], Pongsanarm Tavun[1], Vungkyly Veaky[1], Luangamath Phonesavanh[1], Chonephetsarath Somsanith[1], Brey Paul[1], Buchy Philippe[1], Depaquit Jérôme[2,3]

[1]Institut Pasteur du Laos, Laboratory of Vector-Borne Diseases, Samsenthai Road, Ban Kao-Gnot, Sisattanak District, 3560 Vientiane, Lao PDR

[2]UR ESCAPE, Université de Reims Champagne-Ardenne, ANSES USC PETARD, Reims, France

[3]Centre Hospitalo-Universitaire, pôle de Biologie territoriale, Laboratoire de Parasitologie-Mycologie, 51092 Reims, France

***Corresponding author:** k.vongphayloth@pasteur.la

Abstract

Phlebotomine sandflies are the principal vector of parasites and viruses. In the past decades, very few studies on sandflies and sandfly-borne pathogens have been conducted in Laos. Therefore, the knowledge of sandfly diversity, distribution and their related pathogens are lacking.
Between 2012 and 2024, sandflies were collected using CDC light traps from different types of habitats from different province of Laos and were identified by morphological and molecular methods. Of more than 6,000 sandflies examined, at least 33 species belonging to five genera including *Chinius*, *Idiophlebotomus*, *Phlebotomus*, *Sergentomyia* and *Grassomyia* have been found in Laos. Some more new species of sandflies were described. Our study highlighted the high diversity of phlebotomine sandfly fauna in Laos, which was previously underestimated. However, the taxonomic status of many species in Laos, as well as Southeast Asia, still needs more in-depth study using both morphological characters and molecular methods.

Keywords: Laos, systematic, sandflies

O54 Sand fly fauna investigation in refugee camps and emerging *Leishmania tropica* foci in Cyprus

Gouzelou Stavroula (Evi)[1,2]*, Christodoulou Vasiliki[3], Pavlou Christoforos[4], Antoniou Maria[5], Koliou Maria[1,2]

[1]*Laboratory of Infectious Diseases and Public Health, School of Medicine, University of Cyprus, Nicosia, Cyprus*
[2]*Department of Pediatrics, Archbishop Makarios III Hospital, Nicosia, Cyprus*
[3]*Laboratory of Animal Health, Virology Section, Veterinary Services of Cyprus, Nicosia, Cyprus*
[4]*Biology Department, School of Sciences and Engineering, University of Crete, Heraklion, Crete, Greece*
[5]*Laboratory of Clinical Bacteriology Parasitology Zoonoses and Geographical Medicine, Faculty of Medicine, University of Crete, Heraklion, Crete, Greece*

***Corresponding author:** evi.gouzelou@gmail.com

Abstract

In Cyprus, transmission of *Leishmania donovani* MON-37 is well-established causing human cutaneous and visceral leishmaniasis (HCL, HVL). However, the recent emergence of *Leishmania tropica* causing HCL in the local population and affecting Syrian refugees, proves that human migration and climate change could further impact the epidemiology of leishmaniasis in Cyprus.
In this context, we conducted an entomological survey to evaluate the sand fly fauna and identify sandfly-vector species that could support the establishment of non-endemic *Leishmania* spp., like *L. tropica,* or the increase of HCL/HVL caused by *L. donovani* complex.
Field samplings using CDC light traps were conducted at Larnaka and Nicosia districts, targeting refugee camp sites and recent HCL/HVL foci. Sand fly species identification was based on morphology using established keys, and complemented by DNA barcoding (cox1). Bi-directional sequencing, multiple sequence alignments were performed and used for phylogenetic inference.
Morphological and molecular identification of 772 wild-caught sand flies indicated the predominance of *Sergentomyia* genus (69%). *S. dentata* spp. was found most abundant, followed by *S. minuta* and *S. fallax cypriotica*. Among the *Phlebotomus* genus, *P. tobbi* (44.8%) and *P. papatasi* (44.4%) were the dominant species followed by *P. perfiliewi* (6.4%) and *P. sergenti* (4.2%). Of the 24 blood-fed sandflies, majority were identified as *P. tobbi* (n=10) and *P. papatasi* (n=9).
The presence of *P. sergenti* could support the establishment and spread of *L. tropica.* Overall, the circulation of various competent *Leishmania* vectors in these high-risk areas is an early warning, highlighting the need for systematic vector and disease surveillance.

Keywords: *Leishmania tropica*, emergence, human leishmaniasis, sandfly vectors, Cyprus

O55 Investigations on the presence of sand fly species (Diptera: Psychodidae) in northern Italy and on the prevalence of *Leishmania* spp. in sand flies and reptile hosts

Alvaro Alessandro[1], Cattaneo Giulia Maria[1], Bigoni Fabio[2], Sanchez-Ruiz Lorenzo[1], Mendoza-Roldan Jairo Alfonso[3], Otranto Domenico[3], Molteni Riccardo[1], Varotto-Boccazzi Ilaria[1], Bandi Claudio[1], Epis Sara[1]*

[1]*EntoPar lab, Department of Biosciences, University of Milan, Milan, 20133, Italy*
[2]*Department of Veterinary Medicine and Animal Science, WildlifeHealth Lab, Università Degli Studi di Milano, Via Dell'Università 6, 26900 Lodi, Italy*
[3]*Department of Veterinary Medicine, University of Bari, Valenzano, Bari, 70010, Italy*

***Corresponding author:** sara.epis@unimi.it

Abstract

In Italy, as in other Mediterranean regions, leishmaniasis is caused by *Leishmania infantum.* In the southern regions of the country, the reptile-associated *Leishmania tarentolae* has been reported, often in sympatry with *L. infantum*. There is evidence that exposure to *L. tarentolae* may elicit a protective immune response toward infections by pathogenic *Leishmania* spp. Little is known about sand fly species composition and *Leishmania* prevalence in northern Italy, and no data regarding the occurrence of *L. tarentolae* in sand flies and reptiles in this area have been published.
Sand flies were sampled at 12 sites in Bergamo district in 2022 and 2023 using light and sticky traps. Synanthropic Italian wall lizards *Podarcis muralis* were captured around the same sites, and samples of blood, faeces, tissue, as well as salivary and cloacal swabs, were collected.
A total of 678 sand flies were collected in 7/12 sites and were identified as *Phlebotomus perniciosus*, *Phlebotomus neglectus,* and *Sergentomyia minuta*. Seventy-five samples were collected from lizards. Screening for *Leishmania* spp. was performed in sand flies and reptile samples with PCR employing pan-*Leishmania* primers targeting the ITS-1 region. In sand flies, the prevalence of *Leishmania* spp. was 22.5% considering all the collected females, and the parasite DNA was detected in females of all the three sampled species. Positivity to *Leishmania* spp. was also observed in all the categories of lizard samples, with 96% prevalence in blood samples. A total of 53 *L. (Sauroleishmania) tarentolae*-like sequences were obtained, after sequencing of PCR products.

Keywords: *Phlebotomus*, *Sergentomyia*, *Sauroleishmania*, reptiles, northern Italy

EU funding provided by the NextGeneration EU-MUR PNRR Extended Partnership initiative on Emerging Infectious Diseases (Project No. PE00000007, INF-ACT)

O56 Sand flies (Diptera: Phlebotominae) in the Republic of Kosovo: distribution, ecology and pathogen circulation

Hoxha Ina[1]*, Xhekaj Betim[2], Platzgummer Katharina[1], Dvořák Vit[3], Ayhan Nazli[4], Charrel Remi[4], Muja Bajraktari Nesade[5], Milchram Markus[6], Obwaller Adelheid G.[7], Poeppl Wolfgang[8], Stefanovska Jovana[9], Cvetkovikj Aleksandar[9], Walochnik Julia[1], Trájer Attila J.[10], Sherifi Kurtesh[2], Kniha Edwin[1]

[1]Center for Pathophysiology, Infectiology and Immunology, Institute of Specific Prophylaxis and Tropical Medicine, Medical University Vienna, Vienna, Austria
[2]Faculty of Agriculture and Veterinary, University of Prishtina "Hasan Prishtina", Prishtinë, Kosovo
[3]Department of Parasitology, Faculty of Science, Charles University Prague, Prague, Czech Republic
[4]Unite des Virus Emergents (Aix- Marseille Univ – IRD 190 – Inserm 1207 – IHU Mediterranee Infection), Marseille, France
[5]Faculty of Mathematics and Natural Sciences, University of Prishtina "Hasan Prishtina", Prishtinë, Kosovo
[6]Institute of Zoology, BOKU University, Vienna, Austria
[7]Division of Science, Research and Development, Federal Ministry of Defence, 1090 Vienna, Austria
[8]Department of Dermatology and Tropical Medicine, Military Medical Cluster East, Austrian Armed Forces, Vienna, Austria
[9]Department of Parasitology and Parasitic Diseases, Faculty of Veterinary Medicine-Skopje, Ss. Cyril and Methodius University in Skopje, Skopje, North Macedonia
[10]University of Pannonia, Sustainability Solutions Research Lab, Hungary

***Corresponding author:** ina.hoxha@meduniwien.ac.at

Abstract

Phlebotomine sand flies (Diptera: Phlebotominae) are small, hematophagous insects and the principal vectors of *Leishmania* spp. and phleboviruses. Sand fly-borne diseases are highly endemic in the Balkans but often understudied. In our study, we aimed to update the known sand fly distribution in the Republic of Kosovo, assess the factors influencing their presence, and evaluate local pathogen circulation. To address this, two sand fly surveys using CDC light traps were conducted in understudied regions of Kosovo in 2022 and 2023. Morphological identification was confirmed by barcoding, and host-feeding preferences were assessed by blood meal analysis. PCR-based methods and sequencing were used to screen for *Leishmania* spp. and phleboviruses. Additional occurrence data from two previous surveys were incorporated to generate distribution maps and conduct environmental analyses. Altogether, more than 3500 sand flies were caught in all seven regions of Kosovo, and barcodes of eight endemic species were generated. Environmental analyses identified two geographical groups with notable differences between species. We analyzed blood meals of five sand fly species, identifying seven different host species, with *Ph. neglectus* and *Ph. perfiliewi* being the most prevalent species. *Leishmania* DNA was amplified from two sand fly species and further molecularly characterized as *L. infantum*, and three phleboviruses belonging to different serogroups were identified. This study provides the to date most comprehensive sand fly survey and mapping of the currently known distribution in Kosovo. By combining environmental, blood meal analyses and pathogen screening, we identified factors influencing sand fly occurrence and hotspots of pathogen circulation.

Keywords: Phlebotominae, *Leishmania*, Phlebovirus, Kosovo

O57 National Surveillance Network for sandflies in Portugal: the importance of monitoring a less known vector

Amaro Fátima[1,2,3]*, Osório Hugo[1,2], Silva Manuel[1], Freitas Inês Campos[1], Soares Patrícia[1], Vilares Anabela[4], Martins Susana[4], Reis Tânia[4], Gargaté Maria João[4], Alves Maria João[1,2,3], REVIVE Team[5]

[1]*Centre for Vectors and Infectious Diseases Research, National Institute of Health Doutor Ricardo Jorge, Águas de Moura, Portugal*
[2]*Environmental Health Institute, Environment and Infectious Diseases Research Group, Lisbon, Portugal;*
[3]*Centro de Estudos de Ciência Animal (CECA), Instituto de Ciências, Tecnologias e Agroambiente (ICETA), Universidade do Porto, Porto, Portugal*
[4]*National Reference Laboratory of Parasitic and Fungal Infections, National Institute of Health Doutor Ricardo Jorge, Lisboa, Portugal*
[5]*Administrações Regionais de Saúde do Algarve, Alentejo, Centro, Lisboa e Vale do Tejo e Norte*

***Corresponding author:** fatima.amaro@insa.min-saude.pt

Abstract

Phlebotomine sandflies play a crucial role in both human and veterinary medicine, acting as vectors for *Leishmania* parasites and the majority of known phleboviruses. In Portugal the REVIVE, programme, a comprehensive national surveillance network working under the Ministry of Health, has been operational since 2008, initially targeting mosquitoes and later expanding to include ticks in 2011 and sandflies in 2016. Regarding the latter, the primary objectives of REVIVE are to identify the existing species in our country, ascertain which pathogens are circulating among them and provide actionable insights for prevention and control measures when necessary. In this way, annually, from May to October, health technicians collect sandflies all over mainland Portugal, with CDC light traps. The collected sandflies are dispatched to the Centre for Vectors and Infectious Diseases Research (National Institute of Health) for species identification and molecular screening of pathogens with PCR assays. Since the beginning of the programme, 1577 sandflies have been collected from 72 municipalities across Portugal. Among the five species identified, namely *Ph. ariasi, Ph. papatasi, Ph. perniciosus, Ph. sergenti* and *Sergentomyia minuta*, *Ph. perniciosus* is the most ubiquitous. Until 2022, the screened, sandflies collected in the scope of REVIVE yielded no pathogen detection. However, on 21st September 2023, both Toscana virus and *Leishmania infantum* were detected in separate pools of 30 sandflies each, collected in the same trap in Algarve, the southernmost region of Portugal. While *Leishmania infantum* has been previously detected in sandflies in Portugal, to the best of our knowledge, this is the first time Toscana virus has been detected in its vector in this country, having previously only been reported in vertebrate hosts. These findings highlight the important role of ongoing surveillance efforts in monitoring and understanding the dynamics of sandfly-borne diseases in Portugal.

Keywords: Sandflies, Portugal, National Surveillance network, Toscana virus, *Leishmania infantum*

O58 Assessing Sand Fly Vector Distributions in Spain to Predict Leishmania and Phlebovirus Infection Risks: Insights from the CLIMOS Project.

Berriatua Eduardo[1]*, Alcover M. Magdalena[2], Barandika Jesús F.[3], Cevidanes Miranda Aitor[3], Cuadrado-Matías Raúl[4], Delacour Estrella Sarah[5], Díaz-Sáez Victoriano[6], Fernández Guillermo[5], Fisa Roser[2], Jiménez Maribel[7,8], Lucientes Curdi Javier[5], Martín-Martín Inés[7], Martín-Sánchez Joaquina[6], Martínez-Barciela Yasmina[9], Molina Ricardo[7], Morales-Yuste Manuel[6], Paz Adolfo[10], Pérez-Cutillas Pedro[1], Polina Alejandro[9], Risueño José[1], Roca-Geronès Xavier[2], Ruiz-Fons Francisco[4], Sánchez Andrade Rita[10]

[1]University of Murcia, Murcia, Spain, [2]University of Barcelona, Barcelona, Spain, [3]Basque Institute for Agricultural Research and Development (NEIKER), Derio, Spain, [4]Spanish Game & Wildlife Research Institute (IREC), Ciudad Real, Spain, [5]University of Zaragoza, Spain, [6]University of Granada, Granada, Spain, [7]Instituto de Salud Carlos III, Madrid, Spain, [8]Collaborative Biomedical Research Center in Infectious Diseases (CIBERINFEC), Instituto de Salud Carlos III, Madrid, Spain, [9]University of Vigo, Vigo, Spain, [10]University of Santiago de Compostela, Lugo, Spain

***Corresponding author:** berriatu@um.es

Abstract

Understanding Phlebotomine sand fly vector distributions is crucial for predicting the risk of *Leishmania* and Phlebovirus infections. As part of the CLIMOS project (https://climos-project.eu/), which aims to develop a sand fly distribution Early Warning System (EWS) for Europe and neighbouring countries, we investigated of sand fly density across a wide range of bioclimatic zones in Spain, between April and November 2023. Sand fly surveillance involved setting up 51 CDC light traps in animal premises across 23 localities for two consecutive nights each month, resulting in a total sampling effort of 884 trap-nights. Out of these, 6,664 sand flies were collected on 41% of the trap-nights, with 53% of them being females. Morphological identification revealed species composition, with 77% being *Phlebotomus perniciosus*, 14% *P. papatasi*, 7% *Sergentomyia minuta*, 1% *P. ariasi*, 0.4% *P. sergenti*, and one *P. mascittii* specimen. Sand fly density and species diversity exhibited significant variation within and between zones, showing strong seasonality and dependence on climate. The majority of specimens were collected between May and September in Eastern Mediterranean and Central Continental areas, characterized by low precipitation, hot summers, and mild or cold winters. Fewer sand flies, including *P. mascittii* and a relatively large proportion of *P. ariasi*, were found along the Northern Atlantic fringe. These findings provide valuable insights into the relationship between sand fly distribution and environmental variables at both small and large geoclimatic scales, contributing to the CLIMOS objective of developing an EWS.

Keywords: Spain, surveillance, sand flies, climate, light traps

Funding: The CLIMOS consortium is co-funded by the European Commission grant 101057690 and UKRI grants 10038150 and 10039289. The six Horizon Europe projects, BlueAdapt, CATALYSE, CLIMOS, HIGH Horizons, IDAlert, and TRIGGER, form the Climate Change and Health Cluster.

O59 *Sergentomyia ssp* in the Tunisian *Leishmania major focus* of Sidi Bouzid: Diversity and *Leishmania* infection

Ghrab Jamila[1,2]*, Benabid Meriam[1], Bellali Hedia[3], Aoun Karim[1], Bouratbine Aida[1]

[1]*Lab of Medical Parasitology, Biotechnology and Biomolecules LR20-IPT-06, Institut Pasteur of Tunis, Manar University, Tunisia*
[2]*High Institute of Sciences and Technology of Environnement, Borj-Cedria, Carthage University, Tunisia*
[3]*Faculty of Medecine Tunis, Tunisia*

***Corresponding author:** abdelkafi.jamila@gmail.com

Abstract

Phlebotomus (*Ph.*) *papatasi* is the proven vector of *Leishmania* (*L.*) *major i*n Tunisia. *Sergentomyia* species are present in *L. major* foci, however, their potential role as vectors deserves to be explored. This study aimed to investigate the role of *Sergentomyia* ssp in *L. major* transmission in the endemic zoonotic cutaneous leishmaniasis focus of Sidi Bouzid, central Tunisia. During August 2016, 559 sandflies (280 males, 279 females) were collected by CDC light traps set according to a short transect in rodent burrows, in irrigated field and 3 houses located at increasing distances from burrows. Male specimens were morphologically identified at the species level. DNA was extracted from all females and used for barcoding, *Leishmania* infection detection by kDNA qPCR and blood meal analysis. *Leishmania* species were identified by ITS-1 PCR-sequencing. *Phlebotomus papatasi* was a dominant species, more abundant indoor and rodent burrows either for males or for females. Indoors, its relative abundance decreased with distance from rodent burrows. *Sergentomyia ssp* females were more abundant and more diverse than predicted by males with close relative abundance in the different habitats. Five *Sergentomyia* species were identified*: Se. fallax, Se. dreyfussi, Se. antennata, Se. minuta and Se. ssp*. Nine female specimens were found infected by *L. major*. Five specimens were identified as *Ph. papatasi* and 4 belonged to the *Sergentomyia* genus. These latter were 2 unfed and gravid *Se.* ssp females collected in rodent burrows and 2 engorged *Se. dreyfussi* females were fed on humans and collected from irrigated fields and the nearest house to rodent biotopes.

Keywords: *Sergentomyia*, *Leishmania major* infection, Tunisia

O60 Sand fly fauna in leishmaniasis foci in Algeria: Species composition and blood meal sources studied by complementary techniques

Benallal Kamal Eddine[1,2]*, Halada Petr[3], Depaquit Jérôme[4,5], Manseur Hamza[6], Lafri Ismail[7], Mefissel Mohammed[8], Dib Yassine[8], Volf Petr[1], Dvorak Vit[1]

[1]*Department of Parasitology, Faculty of Science, Charles University, Prague, Czech Republic*
[2]*Laboratory of Arboviruses and Emergent Viruses, Institut Pasteur of Algiers, Algeria*
[3]*Institute of Microbiology of the Czech Academy of Sciences, Biocev Vestec, Czech Republic*
[4]*UR ESCAPE, Université de Reims Champagne-Ardenne, USC ANSES PETARD, Reims, France*
[5]*Laboratoire de Parasitologie, Pôle de Biologie Territoriale, CHU, Reims, France*
[6]*Laboratory of Biotechnology Related to Animal Reproduction (LBRA), Institute of Veterinary Sciences University of Blida 1, Algeria*
[7]*Department of Bacterial and Viral Veterinary, Institut Pasteur of Algiers, Algeria*
[8]*Prevention Laboratory, Local Public Health Establishment Ibn-Sina of Illizi, Algeria*

***Corresponding author:** benallak@natur.cuni.cz, kbenallal@pasteur.dz

Abstract

Sand fly-borne diseases, especially leishmaniases, are endemic in Algeria where several *Leishmania* species circulate in traditional and newly emerging foci. In our study, 2576 sand flies were collected by CDC light traps and sticky papers in provinces of Béjaïa and Sétif (Northern Algeria), Ghardaïa and Illizi (north and central Sahara) that report cases of human leishmaniasis, to understand the role of the local sand fly species in the transmission of leishmaniasis. Species identification was achieved by a combination of morphological and molecular techniques (sequencing analysis, MALDI-TOF MS protein profiling), and blood sources of engorged females were identified using MALDI-TOF peptide mass mapping. In Northern Algeria *Phlebotomus perniciosus* was very dominant (1836 out of 1940 specimens identified) while in south Algeria the most dominant species were *Ph. papatasi* (263 out of 430 specimens in Illizi) and *Sergentomyia antennata* (78 of 206 specimens in Ghardaïa). Out of 72 specimens analysed by MALDI-TOF protein profiling, 97% (70/72) were correctly identified and assigned to 8 species belonging to *Sergentomyia* and *Phlebotomus* genera. In addition, molecular techniques and detailed morphological assessment allowed to describe a new species belonging to *Sergentomyia* subgenus (*Sergentomyia imihra* n. sp). MALDI-TOF peptide mass mapping determined successfully 91% (174/192) of blood meal sources, cow and goat being the most prevailing hosts. One mixed blood meal (human and goat) was also identified. This study showed the potential of combining traditional morphological analysis and complementary molecular techniques in field studies of sand flies which can be extended to other arthropods of medical interest.

Keywords: MALDI-TOF mass spectrometry, *Phlebotomus*, *Sergentomyia*, blood meal, Algeria

O61 MALDI-TOF MS protein profiling: A promising tool for identification of sibling species within the *Phlebotomus perniciosus / longicuspis* complex

Halada Petr[1]*, Vojtkova Barbora[2], Mhaidi Idris[3], Sadlova Jovana[2], Lemrani Meryem[3], Dvorak Vit[2]

[1]*Institute of Microbiology of the Czech Academy of Sciences, BioCeV, Vestec, Czech Republic*
[2]*Department of Parasitology, Faculty of Science, Charles University, Prague, Czech Republic*
[3]*Laboratory of Parasitology and Vector-Borne-Diseases, Institut Pasteur du Maroc, Casablanca, Morocco*

***Corresponding author:** halada@biomed.cas.cz

Abstract

Within the subgenus *Larroussius*, *Phlebotomus perniciosus* and *Ph. longicuspis* are among principal vectors of *Leishmania infantum* that causes zoonotic leishmaniasis in humans and animals. Both species are distributed, often sympatrically, in the western part of the Mediterranean basin, are closely related and constitute a species complex. Males of *Ph. perniciosus* occur in two genetically similar morphotypes (PN, PNA). The atypical morphotype PNA is easily confused with males of *Ph. longicuspis sensu lato* which is represented by two sibling species (LC, LCx) whose males can be differentiated by the morphology of genitalia while females are considered morphologically indistinguishable and identifiable only by DNA sequencing methods. This complexity challenges correct understanding of geographical distribution and involvement in leishmaniasis transmission cycles for species within the complex that may have varying vectorial roles and epidemiological significance.
An entomological survey in Ouazzane province in northern Morocco, a focus on cutaneous leishmaniasis, revealed the presence of all members of the *Ph. perniciosus/longicuspis* complex, offering a unique possibility to test MALDI-TOF MS protein profiling as an alternative, rapid and cost-effective method for their correct species identification. In total, 102 specimens of both sexes were analyzed. The obtained protein profiles allowed to conclusively distinguish *Ph. perniciosus/longicuspis* specimens, and moreover, to reliably discriminate LC/LCx sibling species, whereas the spectra of PN/PNA morphotypes were found as indistinguishable. The correctness of identifications provided by mass spectrometry was confirmed by CytB sequencing. These results suggest that MALDI-TOF MS protein profiling may be a promising alternative approach to unravel the *Phlebotomus perniciosus/longicuspis* species complex.

Keywords: MALDI-TOF mass spectrometry, species identification, species complex

Funding: Czech Science Foundation (GA23-06299S) and LeiSHield-MATI project (H2020-MSCA-RISE-2017-778298).

O62 First report of a cavernicolous population of *Phlebotomus neglectus* (Diptera: Psychodidae) breeding at suboptimal temperatures in a karst tourist cave in Bulgaria

Mikov Ognyan[1]*, Bekchiev Rostislav[2], Langourov Mario[2], Hodzhev Yordan[1], Tsafarova Borislava[1], Panaiotov Stefan[1], Simov Nikolay[2]

[1]*National Centre of Infectious and Parasitic Diseases, Sofia, Bulgaria*
[2]*National Museum of Natural History, Bulgarian Academy of Sciences, Sofia, Bulgaria*

***Corresponding author:** mikov@ncipd.org

Abstract

Caves are specific ecological niches with extreme living conditions. They provide a natural shelter for many species of bats and dipterans. *Leishmania infantum*, the protozoan parasite causing the zoonotic visceral leishmaniasis, was detected in at least one of the 45 species of bats in Europe.
To identify the potential vectors of zoonotic diseases that may be associated with bat colonies, guano and cave environments, sampling was conducted in the caves of Orlova Chuka, Parnitsite, and Devetashka from January 2022 to February 2023 using pitfall traps both at the entrances and in internal completely dark halls isolated from the outside environment, near overwintering and breeding bat colonies. Additional sampling was performed in April 2024 using BG Pro traps with UV light, and manual collection on guano piles.
Two males and four gravid females of *Ph. neglectus* were collected from pitfall traps from the internal dark zone of Orlova Chuka cave. Additionally, three males and a gravid female were captured on the bat guano, as well as nine non-blood-fed females in the light traps. The air in the cave hall has a temperature between 13,2 and 14,5°C and a relative humidity of 97-99%.
The presence of male and female individuals of *Ph. neglectus* in different stages of maturation is evidence of a population breeding in complete darkness and at temperatures close to the threshold of 13°C. The proximity of the bat colony suggests a trophic relationship between the insects and the mammals with a potential role as natural reservoirs of *L. infantum*.

Keywords: caves, bats, sand flies, vectors, leishmaniasis

O63 Flebocollect Project: citizen science as a tool to enhance sand fly surveillance and community engagement in Spain

Gálvez Rosa[1,4]*, Milagres Tarcísio[1,2,3], López de Felipe Marcos[1,5], Bermejo San Frutos Ángela[1], Mora Ana I.[4]

[1]*Flebocollect Medical Entomology Citizen Science Group, Madrid, Spain*
[2]*Department of Microbiology, Immunology and Parasitology, Universidade Federal Do Rio Grande do Sul, Porto Alegre, Brazil*
[3]*Instituto de Higiene e Medicina Tropical, Universidade NOVA de Lisboa, Lisboa, Portugal*
[4]*Department of Specific Didactics, School of Education and Teacher Training, Universidad Autónoma de Madrid, Madrid, Spain*
[5]*Laboratory of Medical Entomology, National Center for Microbiology, Instituto de Salud Carlos III. Majadahonda, Madrid, Spain*

***Corresponding author:** rosa.galvez@uam.es

Abstract

Citizen science initiatives represent a new approach to engaging the public in controlling emerging diseases. The Flebocollect project is an example of this paradigm, mobilizing school students and public in general to monitor sand flies, the vectors of *Leishmania* parasites and Phlebovirus. Held in seven cities in Spain, the project managed to engage around 1,911 participants and collect 1088 specimens of sand flies using more than 300 DIY light traps. Four different species were identified, highlighting *Phlebotomus perniciosus*, the main vector in Europe. Using online application (www.flebocollect.com/user), entomological data was used to create a publicly accessible sand fly monitoring map (www.flebocollect.com/mapa), providing information about their distribution. In addition to enriching data collection, student involvement in scientific inquiry also stimulated public health education and engagement in alignment with the Sustainable Development Goals. Innovative strategies like Flebocollect exemplify how citizen science can boost vector-borne disease research and surveillance while promoting education and public participation in science. Furthermore, the success of the project in Spain suggests the possibility of similar ventures in other countries

Keywords: citizen science, sustainable vector control, sand flies, Leishmaniasis, public engagement

O64 Hidden diversity in Monticola Series (Diptera, Psychodidae, Phlebotominae)

Rodrigues Bruno L.[1]*, Oliveira Alessandra G.[1,2], Silva Leonardo E.H.[1], Santos Thiago Vasconcelos[3], Oliveira Lidiane N.C.[3], Rêgo Felipe D.[4], Andrade Andrey J.[5], Maia Glédson B.[1], Pinto Israel S.[6], Andrade-Filho José D.[4], Galati Eunice A.B.[1]

[1]*School of Public Health, University of São Paulo (FSP USP), Brazil,* [2]*Institute of Biosciences, Federal University of Mato Grosso do Sul (INBIO UFMS), Campo Grande – MS, Brazil,* [3]*Parasitology Section, Evandro Chagas Institute (IEC), Ananindeua – PA, Brazil,* [4]*Leishmaniasis Study Group, René Rachou Institute, Oswaldo Cruz Foundation (IRR), Belo Horizonte – MG, Brazil* [5]*Basic Pathology Department, Federal University of Paraná (UFPR), Curitiba – PR, Brazil,* [6]*Federal Institute of Education, Science and Technology of Pará (IFPA), Itaituba – PA, Brazil*

***Corresponding author:** rodrigues.bruno@usp.br

Abstract

The Monticola series comprises two anthropophilic and widely distributed species in Brazil: *Pintomyia monticola* (Costa Lima, 1932) and *Pintomyia misionensis* (Castro, 1959). They mainly occur in the Atlantic Rainforest, and it is known that *Pi. monticola* comprises at least two well-structured genetic lineages in Brazil using a fragment of the cytochrome c oxidase subunit I (*COI*) gene. Here, we aim to elucidate the taxonomic status of this group using integrative taxonomy tools. Collections were performed in nine localities of four Brazilian states, and *COI* fragments were sequenced and merged with publicly available data. Several single-locus species delimitation algorithms, genetic distance metrics, phylogenetic trees, and haplotype networks were used to uncover cryptic diversity and population structure within *Pi. monticola* and *Pi. misionensis*. The resulting genetic clusters were then tested for morphological differences through linear and geometric morphometry of several characters. We analyzed 152 *COI* sequences, comprising 48 haplotypes. The maximum intraspecific *p* distances were 8.21% (mean 4.17%) and 9.12% (mean 4.4%) for *Pi. monticola* and *Pi. misionensis*, respectively, while interspecific ones ranged from 10.94% to 14.09% (mean 12.33%). Phylogenetic gene trees showed well-supported clades for both species, with clear patterns of structuring within them. Species-delimitation algorithms split our dataset into at least two or three putative species for each taxon. Morphometric analyses were significant for wing shape variation and some linear measurements (mainly of the head) when comparing specimens of different genetic clusters. Our results indicate strong genetic structuring of Monticola series species, confirmed by morphometrics, indicating two possible cryptic species complexes.

Keywords: integrative taxonomy, species delimitation, cryptic species, morphometrics

Funding: Fundação de Amparo à Pesquisa do Estado de São Paulo (FAPESP) – Finance code 2023/03715-2; Coordenação de Aperfeiçoamento de Pessoal de Nível Superior - Brasil (CAPES) - Finance Code 001.

O65 Sand fly research in Central Europe – the past, the present and the future

Platzgummer Katharina[1]*, Oerther Sandra[2], Dvořák Vit[3], Sadlova Jovana[3], Vojtkova Barbora[3], Becvar Tomas[3], Volf Petr[3], Obwaller Adelheid G.[4], Poeppl Wolfgang[5], Hoxha Ina[1], Trájer Attila J.[6], Walochnik Julia[1], Kniha Edwin[1]

[1]Center for Pathophysiology, Infectiology and Immunology, Institute of Specific Prophylaxis and Tropical Medicine, Medical University Vienna, Kinderspitalgasse 15, 1090 Vienna, Austria
[2]Institute of Global Health, Heidelberg University, Im Neuenheimer Feld 130/3, 69120 Heidelberg, Germany
[3]Department of Parasitology, Faculty of Science, Charles University Prague, Viničná 7, 128 43 Prague, Czech Republic
[4]Division of Science, Research and Development, Federal Ministry of Defence, Roßauer Lände 1, 1090 Vienna, Austria
[5]Department of Dermatology and Tropical Medicine, Military Medical Cluster East, Austrian Armed Forces, Brünner Straße 238, 1210 Vienna, Austria
[6]University of Pannonia, Sustainability Solutions Research Lab, Egyetem u. 10. H-8200, Hungary

***Corresponding author:** katharina.platzgummer@meduniwien.ac.at

Abstract

In Europe, sand flies (Diptera: Phlebotominae) are typical Mediterranean faunal elements and various confirmed vector species for *Leishmania* spp. and phleboviruses are endemic. While their geographical distribution and involvement in local transmission cycles have been known for many decades in many parts of the Mediterranean region, the Central European sand fly fauna remains understudied. Only 25 years ago, the first sand fly record has been reported from Germany, marking the onset of following surveys in Austria and nearby regions. To date, *Phlebotomus mascittii* has been identified as the predominant species in Central Europe with established local populations in several countries while reports of other species such as *Ph. perniciosus* in Germany and *Ph. simici* in Austria are rare. In this study, we provide an update of sand fly distribution in Central Europe and highlight aspects of biology, ecology and disease transmission by combining historical and recent data. We present updated distribution maps and molecular screenings of sand flies as well as potential reservoir animals for sand fly-borne pathogens. Globalization and climate change may lead to the expansion of local populations and to the spread of new species to previously non-endemic parts of Central Europe. Assessing the factors that promote the appearance and spread of sand flies is crucial to monitor and understand future medical and veterinary risks of their presence.

Keywords: *Phlebotomus mascittii*, climate change, ecology, postglacial colonization

O66 Phlebotomine sandflies distribution and abundance in France: a systematic review

Prudhomme Jorian[1]*, Depaquit Jérôme[2], Robert-Gangneux Florence[1]

[1]Univ Rennes, Inserm, EHESP, Irset (Institut de Recherche en Santé Environnement Travail), UMR_S 1085, 35000, Rennes, France
[2]Université de Reims Champagne-Ardenne, Faculté de Pharmacie, UR EpidémioSurveillance et Circulation de Parasites dans les Environnements (ESCAPE), and ANSES, USC Pathogènes-Environnement - Toxoplasme-Arthropodes- Réservoirs- bioDiversité (PETARD), Reims, France; Centre Hospitalo-Universitaire, Laboratoire de Parasitologie-Mycologie, 51092, Reims, France

***Corresponding author:** jorian.prudhomme@hotmail.fr

Abstract

Global changes contribute to alter the presence and activity of phlebotomine sandflies and the distribution of the pathogens they transmit (e.g. *Leishmania* and phleboviruses), leading to their possible spread in northern France. It is important to identify and characterise the presence and abundance of potential vectors in order to predict the evolution of these pathogens and control their spread. However, there are no recent publications describing the distribution of sandfly species in France. Therefore, we conducted a systematic review to provide an overview of the distribution and abundance maps over time and a simplified dichotomous key for the French sandfly species, excluding overseas territories. A total of 2,646 documents were retrieved, of which 552 were read and 228 analysed. Seven sand fly species were identified in France: *Phlebotomus perniciosus*, *P. ariasi*, *P. papatasi*, *P. mascittii*, *P. perfiliewi*, *P. sergenti* and *Sergentomyia minuta*. However, there are very few studies that focus on the distribution and abundance of these different species over time. In this work, the available knowledge on these different species was reviewed, an abundance map of occurrence by species was created and a simplified key to identify the sandflies present in France was established. Our aim was to be as comprehensive as possible and to provide a basis for future comparisons of field data that will be generated in the future.

Keywords: systematic review, PRISMA, sand flies, distribution, France

O67 *Phlebotomus perfiliewi* as incriminated vector species in endemic area of Tuscany region (Central Italy)

Bernardini Ilaria[1], Fiorentino Eleonora[1], Mangiapelo Claudia[1], Bianchi Riccardo[1], Marsili Giulia[2], Di Muccio Trentina[1], Fortuna Claudia[2], Bongiorno Gioia[1]*

[1]*Department of infectious diseases, Vector-borne diseases unit, Istituto Superiore di Sanità, Rome, Italy*
[2]*Department of infectious diseases, National reference laboratory for Arboviruses, Istituto Superiore di Sanità, Rome, Italy*

***Corresponding author:** gioia.bongiorno@iss.it

Abstract

The need to update data on pathogen presence in vectors and the current epidemiological situation in the Tuscany Region (Central Italy) prompted to investigate the circulation of L. infantum and Toscana virus (TOSV). For this reason, an entomological survey was conducted during summer 2022 in Magliano in Toscana (Tuscany region, Central Italy). Collected sand flies were morphologically identified and pooled for molecular analysis. The presence of *Leishmania* spp. was evaluated by nested PCR while for TOSV detection a real time RT-PCR was carried out. From 107 analysed pools of *Phlebotomus perfiliewi*, the positivity for *Leishmania infantum* was observed in 10 (9.3%). The higher prevalence was registered in July (0.3%; *n*= 5), followed by August and September (0.2%; *n*= 5). Similarly, the single prevalence of *L. infantum* in each female resulted 8.6% with a peak in July (11%, *n*=11) followed by September (6.7%, *n*= 9), confirming high circulation. Concerning TOSV, out of 100 only one pool of *Ph. perfiliewi* collected in July resulted positive for TOSV (0.01%), supported by single prevalence. Overall, these results reflect a context strongly influenced by the epidemiological data of leishmaniasis, emphasizing the risk for people that attending in areas where *Ph. perfiliewi* is present. Despite TOSV findings reveal a lower circulation than leishmaniasis, massive investigations for a better understanding of the virus circulation would be carried out.

Keywords: *Phlebotomus perfiliewi,* vector species, *Leishmania*, Toscana virus

POSTER PRESENTATIONS

P1 Evaluation of *Lutzomyia longipalpis* populations (Diptera: Psychodidae: Phlebotominae) in areas with use of deltamethrin-impregnated collars for control of visceral leishmaniasis

Albuquerque Rafaella e Silva[1,2]*, De Andrade Andrey José[3], Beust Quint Bruno[1], Salmen Raffoul Gabriel Elias[1]; Werneck Guilherme Loureiro[4]; Ferreira Rangel Elizabeth[5]; Sierra Romero Gustavo Adolfo[1]

[1]*Center for Tropical Medicine, University of Brasilia, Brasília, DF; Brazil*
[2]*Health Surveillance Secretariat, Ministry of Health, Brasília, DF; Brazil*
[3]*Department of Basic Pathology, Federal University of Paraná, Curitiba, PR; Brazil*
[4]*Institute of Social Medicine, Federal University of Rio de Janeiro, Rio de Janeiro, RJ, Brazil*
[5]*Oswaldo Cruz Institute, Oswaldo Cruz Foundation, Rio de Janeiro, RJ; Brazil*

***Corresponding author:** rafaas28@gmail.com

Abstract

There is little information on the effect of using deltamethrin-impregnated dog collars to control canine visceral leishmaniasis over the phlebotomine population in Brazil. This work aimed to evaluate the impact of the use of 4% deltamethrin-impregnated collars (Scalibor®) in populations of *Lutzomyia longipalpis* by comparing areas submitted to the intervention to areas without this intervention. Phlebotomine captures were carried out for 30 months in four neighborhoods with intense VL transmission in Fortaleza/CE e Montes Claros/MG. We calculated the rates of domicile infestation, relative abundance and *Lu. longipalpis* distribution per point, capture location (intra and peridomicile areas) and area (intervention and non-intervention). In the control area from Fortaleza, the relative abundance was 415 specimens per capture point, whereas in the intervention area it was 159.25, while in Montes Claros, the relative abundance was 5,660 specimens per capture point in control areas, whereas in the intervention area, it was 2,499.4. The use of dog collars was associated with a reduction of captured insects of 15% ($p = 0.004$) and 60% ($p < 0.001$) in Montes Claros and Fortaleza, respectively. We observed a lower vector abundance in the intervention areas, which suggests an effect of the insecticide-impregnated collars on the phlebotomine population.

Keywords: visceral leishmaniasis, impregnated collars, control

P2 Co-circulation of phleboviruses and *Leishmania* in sandflies in the south of Portugal

Amaro Fátima[1,2,3]*, Osório Hugo[1,2], Silva Manuel[1], Freitas Inês Campos[1], Vilares Anabela[4], Martins Susana[4], Reis Tânia[4], Gargaté Maria João[4], Alves Maria João[1,2,3]

[1]*Centre for Vectors and Infectious Diseases Research, National Institute of Health Doutor Ricardo Jorge, Águas de Moura, Portugal*
[2]*Environmental Health Institute, Environment and Infectious Diseases Research Group, Lisbon, Portugal*
[3]*Centro de Estudos de Ciência Animal (CECA), Instituto de Ciências, Tecnologias e Agroambiente (ICETA), Universidade do Porto, Porto, Portugal*
[4]*National Reference Laboratory of Parasitic and Fungal Infections, National Institute of Health Doutor Ricardo Jorge, Lisboa, Portugal*

***Corresponding author:** fatima.amaro@insa.min-saude.pt

Abstract

Phlebotomine sandflies transmit pathogens, with *Leishmania* spp. parasites and phleboviruses being the most notable, impacting human and veterinary health. In Algarve, south of Portugal, *Leishmania* infections in dogs are widespread, alongside reported cases of human febrile illness and neurological diseases caused by Toscana virus. Entomological surveillance was conducted in that region, from May to October 2018 using CDC light traps, as part of a research project. Additionally, since 2016, Algarve has been annually surveyed under the National Vector Surveillance Network (REVIVE). In the laboratory, sandflies were morphologically identified and/or screened for pathogens via PCR. In 2018, 1161 sandflies were collected, and 1130 screened. On May 16th, an unknown *Phlebovirus* species was found in a pool of 20 sandfly females in the same trap as *L. infantum*, which was found in a single *Phlebotomus perniciosus* female. On June 5th, Massilia virus was detected in a pool of 20 females in the same trap along with *L. infantum* identified in a single *Phlebotomus perniciosus* female. In the REVIVE (2016-2023), 631 sandfly specimens were sampled in Algarve, and 565 screened. No pathogens were detected until September 21st 2023, when *L. infantum* and Toscana virus were identified, in the same trap, in two pools of 30 female sandflies each. Altogether, four sandfly species were identified in the region, namely *Ph. ariasi*, *Ph. perniciosus*, *Ph. sergenti* and *Sergentomyia minuta* with evidence indicating *Ph. phlebotomus* as the primary vector of *L. infantum* in Algarve. Co-circulation of *L. Infantum* and phleboviruses in sandfly populations suggests potential dual-pathogen transmission. While some laboratory studies have explored co-infections of phleboviruses and *Leishmania spp*. in hosts and there is evidence of natural epidemiological associations, further research is essential to understand the implications for vectors and hosts, and the necessity for additional control interventions in regions where co-circulation occurs.

Keywords: Sandflies, Portugal, National Surveillance network, Toscana virus, *Leishmania infantum*

This work was partially supported by an FCT project: Phleboviruses in Portugal: vectors, pathogenesis and co-infections (PTDC/DTP-SAP/0859/2014).

P3 Investigation of sandfly diversity and canine *Leishmania* infection in Northern and Central *Leishmania infantum* foci in Tunisia

Ghrab Jamila[1,2]*, Zribi Lilia[1], Bellali Hedia[3], Benabid Mariem[1], Hamdouni Mohamed[4], Jellali Taieb[4], Jemal Arij[5], Bouratbine Aida[1], Oliva Gaetano[6], Aoun Karim[1]

[1]*Lab of Medical Parasitology, Biotechnology and Biomolecules LR20-IPT-06, Institut Pasteur of Tunis, Manar University, Tunisia*
[2]*High Institute of Sciences and Technology of Environnement, Borj-Cedria, Carthage University, Tunisia*
[3]*Faculty of Medecine Tunis, Tunisia*
[4]*CRDA, Sidi Bouzid, Tunisia*
[5]*CRDA, Zaghouan, Tunisia*
[6]*University Federico II, 80137 Naples, Italy*

***Coresponding author:** abdelkafi.jamila@gmail.com

Abstract

Visceral leishmaniasis (VL) caused by *Leishmania* (*L.*) *infantum* is endemic in Tunisia. Visceral leishmaniasis is encountered mainly in the northern part of the country. However, it is also recorded in the Center of Tunisia where it coexists with zoonotic cutaneous leishmaniasis (ZCL) caused by *L. major*. The aim of this work was to investigate sandfly diversity in relation with canine *Leishmania* infection prevalence in northern and Central foci.

Study was conducted in VL focus of Zaghouan and ZCL focus of Sidi-Bouzid. Blood was collected from 137 dogs randomly recruited in Zaghouan (*n* = 74) and Sidi-Bouzid (*n* = 53). All dogs' sera were tested by ELISA (ID vet, France). kDNA RT-PCR was performed on DNA extracts from buffy coats of seropositive dogs. *Leishmania* species identification was done by ITS1 PCR-sequencing. Sandflies were collected by CDC-light traps set in dwelling. Males specimens were morphologically identified. Femeles were individually stored in ethanol 70% to detect *L.* infection (in process).

In Zaghouan, where 14 dogs (18.9%) were seropositive and 6 *Leishmania* DNA were identified as *L. infantum,* entomological survey showed that *Larroussius ssp* were dominant (86%) and *P. perniciosus* the more abundant species among the subgenus (76%). In contrast, in Sidi Bouzid, where *Leishmania* sero-prevalence in dog was lower (n= 5, 9.4%), *Phlebotomus papatasi* dominated *Larroussius* species (59% *versus* 38%). However, the most encontred *Larroussius* species was also *P. perniciosus* (75.7%).

Keyword: canine leishmaniasis, Sandfly, *L. infantum*

P4 Investigation of the susceptibility of sand flies to neonicotinoid insecticides in Southeastern Anatolia of Türkiye, where leishmaniasis is endemic

Arserim Suha Kenan[1*], Çetin Hüseyin[2], Limoncu M. Emin[1], Yetişmiş Kardelen[3], Omondi Zeph N.[4], Töz Seray[5], Babat Sefer Ö.[5], Özbel Yusuf[5], Balcioğlu I. Cüneyt[6]

[1]*Vocational School of Health Services, Manisa Celal Bayar University, Manisa, Türkiye*
[2]*Department of Biology, Faculty of Science, Akdeniz University, Antalya, Türkiye*
[3]*Department of Medical Parasitology, Graduate School of Health Sciences, Ege University, İzmir, Türkiye*
[4]*Department of Biology, Graduate School of Natural and Applied Sciences, Ege University, İzmir, Türkiye*
[5]*Department of Parasitology, Faculty of Medicine, Ege University, İzmir, Türkiye*
[6]*Department of Parasitology, Faculty of Medicine, Manisa Celal Bayar University, Manisa, Türkiye*

***Corresponding author:** suhaarserim@hotmail.com

Abstract

Sand flies are vectors of the genus *Leishmania*, which are the causative agent of leishmaniasis. This study aimed to investigate the effectiveness of four neonicotinoids (thiamethoxam, dinotefuran, acetamiprid and imidacloprid) insecticides on sand fly populations in Southeastern Anatolia, where cutaneous leishmaniasis is endemic.
Sand fly samples were collected from a total of 27 localities in the Southeastern Anatolia region using CDC light traps. Active ingredients were tested at the application dose (0.25 g ai/m^2) and lower doses (0.125 g ai/m^2 0.0625 g ai/m^2) as recommended by the Turkish Ministry of Health for mosquitoes and house flies. In the test protocol recommended by WHO, knock-down rates of flies for 60 minutes and death rates at the end of 24 hours were recorded. KDT_{50} and KDT_{90} knock-down values were calculated by probit regression analysis in IBM SPSS Statistics 20 program.
In the efficacy tests performed with dinotefuran and acetamiprid, 100% mortality was observed after 24 hours at all localities and concentrations. All sand fly populations tested were found to be sensitive to these two active substances. However, in the efficacy tests conducted with thiamethoxam and imidacloprid, resistance was detected in some localities and the presence of resistance in other localities still needs to be confirmed by further analyses.
If imidacloprid and thiamethoxam continue to be used irregularly in this region, resistance will develop in areas where resistance has not yet been recorded. In addition, environmental health and non-target organisms will be negatively affected in areas where resistance is thought to have developed.

Keywords: Sand fly, leishmaniasis, insecticide, neonicotinoid, Türkiye

P5 Epidemiological Insights and Distribution of *Lutzomyia spp*. in Uruguay, 2024

González Telma[1], Cabrera Andrés[1], Díaz Mauricio[1], Froster Paola[1], León Diego[1], Verger Lorenzo[2], Willat Gabriela[2], Basmadjian Yester[1]*

[1]*Departamento de Parasitología y Micología, Facultad de Medicina, Universidad de la República, Uruguay.*
[2]*Unidad de Zoonosis y Vectores, Ministerio de Salud Pública, Uruguay.*

***Corresponding author:** yester@higiene.edu.uy

Abstract

Visceral Leishmaniasis is a parasitic disease affecting nearly all South American countries. It is caused by *Leishmania infantum* and is primarily transmitted by *Lutzomyia longipalpis*. This disease poses a significant public health challenge, making it crucial to control both the parasite and the vector. Since the 1930s, the presence of *Lutzomyia* species has been documented in Uruguay, including *L. gaminarai* in Tacuarembó and Salto, and *L. cortelezzi* in Montevideo. However, since 2010, *L. longipalpis* has been detected in the northern part of the country, specifically in Salto and Artigas (Bella Unión). Starting in 2016, the Ministry of Public Health (MSP) began monitoring these insects in Rivera and Paysandú as well. Over time, the distribution of this vector has increased. In 2019, the first specimens of *L. longipalpis* were recorded in Rivera, a city that had not previously reported its presence. Surveillance in Paysandú has intensified due to its proximity to the Uruguay River, its connection to Salto, and the detection of infected dogs in the area. To date, no specimens of the vector have been captured in Paysandú. In December 2022, due to infected canine cases, the first specimens of *L. longipalpis* were detected in the city of Artigas. The gradual increase of *Lutzomyia spp.* in Uruguay makes it essential to continue monitoring to understand the vector's expansion, ecology, and associated epidemiological risk.

Keywords: visceral leishmaniasis, *Leishmania infantum*, *Lutzomyia longipalpis*, Uruguay, public health

P6 Exploring the Seasonal of *Lutzomyia longipalpis* in Salto City: Insights from the Southernmost Region Where It Was Recorded

Piegas Sofía[1], Cabrera Andrés[2], González Telma[2], Díaz Mauricio[2], Froster Paola[2], León Diego[2], Verger Lorenzo[1], Willat Gabriela[1], Basmadjian Yester[2]*

[1]*Unidad de Zoonosis y Vectores, Ministerio de Salud Pública, Uruguay.*
[2]*Departamento de Parasitología y Micología, Facultad de Medicina, Universidad de la República, Uruguay.*

***Corresponding author:** yester@higiene.edu.uy

Abstract

The recent geographical spread of Leishmania infantum along the borders of Argentina, Brazil, and Paraguay has been highlighted due to the presence of *Lutzomyia longipalpis*. Uruguay has not escaped this harsh reality; the vector was found in 2010, and the parasite circulation appeared in 2015. The detection of the vector in the northern part of the country, primarily in Salto, represents the southernmost area of the continent that it has managed to colonize. Mitochondrial marker studies indicate that the vector populations are very similar to those in neighboring countries. While these genetic patterns allow us to trace the species' introduction route, it's intriguing to observe how these populations behave in our territory. Studying the vector's abundance throughout the year and its relationship with climatic variables would describe how these populations have adapted to the environment. The objective of this study was to explore the seasonal variations of *Lutzomyia longipalpis* and gather abundance data specifically in Salto city, while also examining its correlation with climatic variables. The main finding was the presence of the vector throughout the year, with higher abundances during warmer months. It was surprising to observe the vector even during the colder months of the year. These data help us understand the behavior of *Lutzomyia longipalpis* in the southernmost area of the continent and will enable us to generate knowledge applicable to campaigns aimed at vector control.

Keywords: *Leishmania infantum*, *Lutzomyia longipalpis*, Uruguay, Seasonal variations, Climatic variables

P7 Contribution of real-time PCR targeting kinetoplast DNA to access sandfly infection rate in areas with low *Leishmania infantum* transmission

Bouratbine Aïda[1]*, Weslati Marwa[1], Benabid Meriem[1], Ghrab Jamila[1,2], Souissi Olfa[1], Aoun Karim[1]

[1]*Lab of Medical Parasitology, Biotechnology and Biomolecules LR20-IPT-06, Institut Pasteur of Tunis, Manar University, Tunisia*
[2]*High Institute of Sciences and Technology of Environnement, Borj-Cedria, Carthage University, Tunisia*

***Corresponding author:** aida.bouratbine@pasteur.rns.tn

Abstract

Real-time PCR (qPCR) targeting minicircle kinetoplast DNA (kDNA) is a highly sensitive method that allows *Leishmania* identification. It could detect one flagellate per dissected sandfly gut which legitimates its use as a screening method for study of sandfly infection. Furthermore, kDNA qPCR can determine parasite load, a high burden being correlated with strong evidence of *Leishmania* transmission.
The objective of this work was to access usefulness of kDNA qPCR in screening *Leishmania* infection and determining *L. infantum* vectors in an area with low *Leishmania (L.) infantum* transmission.
A sample of 878 females was collected from a focus of low incidence of visceral leishmaniasis. DNA was extracted separately from each female individual and subjected to kDNA qPCR using a TaqMan probe. *Leishmania* infection was first screened by pools of 5 sandflies' DNA. Then, DNAs from sandflies grouped into each positive pool were analyzed individually to determine the positive specimens and to quantify the parasite load of each specimen. All positive kDNA qPCR specimens were systematically amplified by ITS1-PCR and *Leishmania* species were determined by ITS1 sequencing. Sandfly species was detemined by barcoding.
Real-time PCR identified four positive specimens (0.45%). ITS1-PCR sequencing allowed *L. infantum* identification in only one kDNA qPCR-positive specimen out of 4. This was a *P. perniciosus* female with thousands of parasites. For the other 3 specimens, all were *P. Perniciosus* harboring less than 20 parasites for which *Leishmania* species identification was not possible using ITS1 target.
This work highlights the usefulness of kDNA qPCR in screening *Leishmania* infection.

Keywords: Real-time PCR, kinetoplast DNA, *Leishmania*, *L. infantum*, sandfly

P8 Phlebovirus detection on phlebotomine sand flies in Northern Italy

Carrera Maya[1]*, Calzolari Mattia[1], Scremin Mara[1], Defilippo Francesco[1], Grisendi Annalisa[1], Lelli Davide[1], Lavazza Antonio[1], Dottori Michele[1]

[1]Istituto Zooprofilattico Sperimentale della Lombardia e dell'Emilia Romagna "B. Ubertini", Brescia, Italy

***Corresponding author:** maya.carrera@izsler.it

Abstract

The genus *Phlebovirus* (Phenuiviridae family) includes tri-segmented viruses (L, M, S segments) with a negative-sense RNA genome. Several phleboviruses are transmitted by arthropods, mainly sand flies. The wide diversity of phleboviruses is likely linked to their high mutation rate. Some of these viruses are pathogenic for humans, causing symptoms ranging from summer fevers (Sicilian and Naples viruses) to meningoencephalitis (Toscana virus). In recent years, an increasing number of phleboviruses was detected in sand flies. Sand fly-transmitted diseases are under a surveillance program in Emilia-Romagna and Lombardy regions: from June to October 2023, a total of 61027 sand flies were collected by attractive traps in 247 sites of the two Regions. Of these sand flies, 39230 were grouped in 1179 pools and further screened by molecular analysis with a pan-phlebovirus PCR, followed by sequencing a partial region of the S segment. The analysis revealed the presence of nine different viruses: the well-known Toscana virus (8 samples), Fermo virus (103), and three related viruses differing for the M segment named Ponticelli viruses I, II, III (34); less common viral strains isolated were Corfou virus (10) and Punique virus (2). Two other phleboviruses were also detected but not yet identified, one of which was present in 22 pools of sand flies.
All these viruses do not have well-established potential pathogenicity for humans and animals and deserve more deep investigation. Such characterization could be achieved by screening human and animal blood samples, developing specific PCR protocols, or through seroneutralization assays, employing viruses isolated from sand flies.

Keywords: *Phlebovirus,* Toscana virus, Fermo virus

P9 The phlebotomine sand fly fauna in the foci of Canine leishmaniasis in Zambia

Sanjoba Chizu[1], Tatsuki Sugi[2], Kyoko Hayashida[2], Herman M. Chambaro[3], Enala T. Mwase[4], Boniface Namangala[4], Yasuyuki Goto[1]*

[1]*Graduate School of Agricultural and Life Science, The University of Tokyo, Japan*
[2]*International Institute for Zoonosis Control, Hokkaido University, Japan*
[3]*Department of Veterinary Services, Ministry of Fisheries and Livestock, Zambia*
[4]*School of Veterinary Medicine, The University of Zambia, Zambia*

***Corresponding author**: aygoto@g.ecc.u-tokyo.ac.jp

Abstract

Dogs are the main reservoir of *Leishmania infantum*, causing canine leishmaniasis that leads to death in symptomatic dogs if left untreated. In Africa, *L. infantum* causes visceral leishmaniasis in humans, mainly in North Africa, and brings serious health issues. In Zambia, although human visceral leishmaniasis was reported in 1973 and 1976, and canine leishmaniasis in 1997, the disease appears to be one of the less recognized or under-reported parasitic diseases in the country. There have been no reports on sand flies inhabiting Zambia. Therefore, we conducted in 2022 the first entomological survey of sand fly fauna in Zambia using CDC light traps at Livingstone, Southern-province where three canine leishmaniasis due to *L. infantum* were reported. Five Sergentomyia species: *S. congolensis*, *S. africana africana*, *S. saliburiensis*, *S. bedfordi*, *S. antennata* and one *Phlebotomus* spp. were identified by partial sequences of mitochondrial cytochrome c oxidase subunit I gene and morphological characters. S. congolensis was the most predominant *Sergentomyia* species and Phlebotomus spp. was scarce. In this survey, 41.2% of *Sergentomyia* spp. were collected from the chicken house, 15% from the sheep house, and 12.2% from the pig house. In 2023, the surveys of sand flies were also conducted in the capital, Lusaka, and *S. congolensis* was predominant. Together with recently accumulating reports on detecting *Leishmania* DNA and/or parasites in several *Sergentomyia* spp. posing their potential for transmitting mammalian/canine leishmaniases in the Old World, the competence of the genus *Sergentomyia* as a vector of leishmaniasis in Zambia should be studied.

Keywords: Zambia, canine leishmaniasis, Sergentomyia congolensis

P10 Sand flies (Diptera: Phlebotominae) in an endemic area of American Tegumentary Leishmaniasis (ATL) in the Paraná state, Brazil

Costa-Ribeiro Magda C.V.[1], Morelli Letícia C.[1], Andrade Andrey J.[1]*

[1]*Basic Pathology Department, Federal University of Paraná. Post-graduate Programme in Microbiology, Parasitology and Pathology, Federal University of Paraná, Curitiba, Paraná, Brazil*

***Corresponding author:** andreyandrade@ufpr.br

Abstract

In Paraná state, the Ribeira Valley region is endemic to American Tegumentary Leishmaniasis (ATL). Aimed to investigate the sand fly fauna in a rural area, species were identified, observing their mensal frequency and the relation between sand fly density and abiotic factors. Positive points for ATL were evaluated for two years (July 2016-July 2018) using CDC light traps (during three consecutive nights in peridomicile) and Shannon trap (during one night in forested area) with a sampling effort of 8,640 hours e 72 hours of capture, respectively. Specimens' density was correlated with abiotic factors collected by meteorological station; for statistical analysis was used the program BioEstat v. 5.3. Sand fly fauna was composed by 13 species, being the *Nyssomyia neivai* (93.9%) more frequent. This species is the main vectors of *Leishmania braziliensis* and it has been found naturally infected by *Leishmania infantum* in an area endemic for Visceral Leishmaniasis (VL), where there is no presence of *Lutzomyia longipalpis*. *Brumptomyia* genus shows more diversity composed by six species. Phlebotomine specimens were present all years regardless of the climatic variables being more frequent after August and less after May. After results we provide data for the Health Secretary about the distribution and seasonality of the species that occur in the area contributing to the control and monitoring of ATL cases in an endemic area for ATL, as preconized by the Ministry of Health of Brazil.

Keywords: *Nyssomyia neivai*, American Tegumentary leishmaniasis, Ribeira Valley.

P11 Sand fly fauna in region of implementation of dog collars impregnated with Deltamethrin in Timon, Maranhão, Brazil, an endemıc area for visceral leishmaniasis

Leite G.D.S. Jackellyne[1,2], Infran O.M. Jucelei[3], Oliveira G. Alessandra[3,4], Galvis-Ovallos Fredy[4], Werneck L. Guilherme[5], Cruz P. Maria do Socorro[1]*

[1]*Program of Applied Technologies to Animals of Regional Interest, Federal University of Piauí, Brazil*
[2]*Federal Institute of Education, Science and Technology of Maranhão, Brazil*
[3]*Institute of Biosciences, Federal University of Mato Grosso do Sul, Brazil*
[4]*Faculty of Public Health, University of São Paulo, Brazil*
[5]*Institute of Public Health Studies, Federal University of Rio de Janeiro, Brazil*

***Corresponding author:** mspcruz@ufpi.edu.br

Abstract

Leishmaniasis is a global health problem, affecting over 1 billion people worldwide. Brazilian government has adopted vector control as one strategy to control leishmaniasis. The main goal was to study sand fly fauna in region of implementation of dog collars impregnated with deltamethrin in Timon, Maranhão, Brazil. Two areas were selected in Timon: control and treatment, where dogs were fitted with collars impregnated with deltamethrin. In each area, 10 households were selected to capture sandflies in peridomestic and intradomestic environments. Twenty-five months of captures were carried out, starting in August 2021. A total of 13,347 sandflies were captured (11,092 males and 2,255 females) from species *Lutzomyia longipalpis*; *Nyssomyia whitmani*; *Evandromyia lenti*; *Evandromyia evandroi*; *and Brumptomyia brumpti*; with *Lutzomyia longipalpis* being the most frequent (n=12,748). Control area had a higher number of insects (n=7,109), males (n=5,881), in the peridomestic environment (n=6,017), and at point 1 (n=1,367). In treatment area, more males (n=5,211) and more sandflies in peridomestic environment (n=5,187), and at point 13 (n=1,270) were captured. In November 2022 was captured the highest number of insects in control (n=901) and treatment areas (n=1,460). The study presented a sand fly fauna composed of five species, with a predominance of *Lutzomyia longipalpis*. In both areas, there was a higher frequency of males in the peridomestic environment, and in November 2022. More insects were captured in control area.

Keywords: Insect vector surveillance, Phlebotomine sandflies, vector ecology, Deltamethrin efficacy

P12 A non-destructive DNA extraction protocol for comprehensive vouchering of DNA-barcoded phlebotomine sandflies (Diptera: Psychodidae)

Ding Huicong[1], Torno Majhalia[1], Ng Germaine[1], Tan Denise[1], Tan Cheong Huat[1]*

[1]*Environmental Health Institute, National Environment Agency, Singapore*

***Corresponding author**: tan_cheong_huat@nea.gov.sg

Abstract

Phlebotomine sandflies (Diptera: Psychodidae) are minute biting flies of great public and veterinary health importance. Accurate identification of these vectors is crucial for understanding the diversity of sandflies and identifying vectors and potential vectors for control. In recent years, DNA barcoding using various genetic markers has become an important method to complement the morphological identification of sandflies. However, the method usually involves the destruction of the entire specimen. Here, we present an "easy-to-follow" methodology for the vouchering of sandfly specimens and the generation of their DNA barcodes. This method will cover sandfly collection using Nightcatcher—an innovative trap, handling of specimens, and the non-destructive DNA extraction protocol. Our method successfully adopts the integrated taxonomic approach to preserve key morphological traits necessary for sandfly morphological identification while providing high-quality DNA for PCR-based DNA barcoding. These are critical first steps in sandfly research necessary to achieve accurate species identification and establish repositories to build up a local archive for future research. The method will also generate strong evidence for species description, given that obscure and overlooked morphology can now undergo re-examination after extraction. The genomic data obtained will also serve as an alternative lens to resolve any morphological uncertainties.

Keywords: Non-destructive DNA extraction, Integrated taxonomy, Morphological identification, DNA barcoding

P13 Identification of sand fly blood meals from filter papers by MALDI-TOF MS peptide mapping

Dvorak Vit[1]*, Chaskopoulou Alexandra[2], Hanusniakova Ida[1], Volf Petr[1], Halada Petr[3]

[1]*Department of Parasitology, Faculty of Science, Charles University, Prague, Czech Republic*
[2]*USDA–ARS, European Biological Control Laboratory, Thessaloniki, Greece*
[3]*Institute of Microbiology of the Czech Academy of Sciences, BioCeV, Vestec, Czech Republic*

***Corresponding author:** vit.dvorak@natur.cuni.cz

Abstract

Conclusive host blood identification provides valuable insight into interactions of sand flies with their hosts and potential reservoirs of sand fly-borne pathogens which is crucial for effective control strategies. Matrix-assisted laser desorption/ionization time-of-flight mass spectrometry (MALDI-TOF MS) was demonstrated as a time, labor and cost-effective method for blood meal identification in medically important arthropods including sand flies. To further foster its routine application in field surveys, a protocol to identify blood meals spotted on Whatman filter paper was developed and tested under laboratory conditions using engorged sand flies of three genera (*Lutzomyia longipalpis*, *Phlebotomus perniciosus*, *Sergentomyia schwetzi*) fed on different hosts (mouse, rabbit) and stored for varying times. The method correctly determined experimentally acquired blood meals in the interval 0 – 48 hours post blood feeding in all tested species and from blood spots stored for up to one year at room temperature.
In the second step, the protocol was evaluated using naturally engorged sand flies (*Phlebotomus tobbi*, *Ph. perfiliewi*, *Ph. simici*) from an endemic locality in Greece, successfully analyzing 33/34 samples and identifying a range of human and domestic animal hosts (sheep, goats, pigs, horses, dogs and chickens) including a mixed blood meal. Peptide mass mapping using MALDI-TOF MS of blood meals spotted on filter paper proved to be a powerful and robust technique for identification of sand fly hosts and potentially a valuable tool in entomological surveys targeting vectors of sand fly-borne pathogens.

Keywords: MALDI-TOF, blood meal determination

Funding: Czech Science Foundation GA23-06299S

P_{14} Collection of sand flies using different colors of light-emitting diodes (LEDs), Brazilian Amazon: preliminary results

De Oliveira Janaína Estevo[1], De Souza Lencina Matheus Vitor[2], De Araújo Nascimento Marlon Gabriel[2], Nogueira Letícia Amaral[2], De Ávila Márcia Moreira[3], Ramos Leandro José[2], Fernandes Brilhante Andreia[1,2]*

[1]*Postgraduate Program in Science and Technology in the Western Amazon, Federal University of Acre, Rio Branco, Acre, Brazil*
[2]*Center for Health and Sports Sciences, Federal University of Acre, Rio Branco, Acre, Brazil*
[3]*Federal Institute of Acre, Rio Branco, Brazil*

***Corresponding author:** andreia.brilhante@ufac.br

Abstract

Light traps are the most used models for collecting and monitoring sand flies. Recently, some studies have shown that sand flies can be attracted to light-emitting diodes (LEDs), which are as efficient as incandescent lights. Our study aimed to use different colors of LED lights to capture sand flies and verify the most collected species according to the color of the LEDs. Seven collections were carried out from October 2022 to December 2023 at the Catuaba Experimental Farm, in Senador Guiomard, Acre, Brazilian Amazon. Six CDC light traps were modified by adding LEDs containing the following colors, yellow, red, green, blue, white and ultraviolet, and one of the traps as incandescent light. The traps were installed at 6 pm and collected at 6 pm. A total of 1,463 sandflies were collected, distributed in 12 genera and 28 species, with *Trichophoromyia ubiquitalis* (36.8%) and *Th. auraensis/octavioi* (27.5%) being the most frequent. Regarding the number of insects according to color, the trap with ultraviolet light had the highest frequency of collected insects (24.84%), followed by white light (21.29%), blue (18.8%), green (16.2%), incandescent (10.2%), red (5.7%) and yellow (2.8%). In this preliminary analysis, we concluded that in terms of population density, ultraviolet and white lights obtained more insects collected, and the genus Trichophoromyia was dominant in all traps.

Keywords: Phletobotominae, *Trichophoromyia*, light trap, Amazonia

*P*15 Sand fly fauna in a forest preservation area, Rio Branco, Acre, Western Brazilian Amazon

Moreira de Ávila Márcia[1]; Alcici de Souza Eduardo[2]; Barroso Edmilson Pereira[2]; Gomes Carneiro Ana Carolina[2]; Galati Eunice Aparecida Bianchi[3]; Fernandes Brilhante Andreia[1,2]*

[1]*Federal Institute of Acre, Rio Branco, Brazil*
[2]*Center for Health and Sports Sciences, Federal University of Acre, Rio Branco, Acre, Brazil*
[3]*Department of Epidemiology, Faculty of Public Health, University of São Paulo, São Paulo, Brazil*

***Corresponding author:** andreia.brilhante@ufac.br

Abstract

American cutaneous leishmaniasis is considered an endemic disease, with high incidence in Brazil, with the state of Acre being one of the states that reports the most cases in the country. It also has a great diversity of sand fly species, some of which are implicated in the transmission of *Leishmania* parasites. Furthermore, of the Brazilian Amazon states, Acre has the largest amount of preserved forest areas. Thus, environmental preservation areas play an important role in the biodiversity of different pathogen vector species. The study aimed to describe the sand fly fauna of the Lago do Amapá environmental preservation area, in the municipality of Rio Branco, Acre, Western Brazilian Amazon. Six collections were carried out with modified CDC-type automatic light traps (n=5), installed at 6 pm, with uninterrupted exposure for 24 hours, from December 2020 to September 2021. A total of 343 sand fly specimens were collected, 207 males and 136 females, distributed in 11 genera and 18 species. The species *Evandromyia walkeri* (33.81%) followed by *Pressatia calcarata* (20.60%) and *Evandromyia saulensis* (11.95%) were the most frequent. Other species collected, which are epidemiologically important for the region, were: *Bichromomyia flaviscutellata*, *Migonemyia migonei*, *Nyssomia antunesi*, *Nyssomia whitmani* and *Trichophoromyia auraensis*, however, with a lower population density. We conclude that the Lago do Amapá area has a diversity of species, with some proven potential vectors and others incriminated in the transmission of etiological agents of leishmaniasis, and may be an area of risk of transmission for its visitors.

Keywords: Phletobotominae, *Evandromyia walkeri*, forest area, Amazonia

P16 *Leishmania* hybridization in their sand fly vectors: investigation of the role of Gex1

Fuentes-Carias Marcela[1]*, Späth Gerald F.[1], Louradour Isabelle[1]

[1]*Institut Pasteur, Université Paris Cité, Unité de Parasitologie moléculaire et Signalisation, Paris, France*

***Corresponding author**: marcela.fuentes-carias@pasteur.fr

Abstract

Abstract: *Leishmania* parasites can produce hybrid genotypes through a cryptic sexual reproductive cycle which occurs in the gut of their sand fly vectors. *Leishmania* experimental hybridization can be achieved *in vivo* (by performing artificial sand fly coinfection) or *in vitro*. The frequency of hybrid formation is higher *in vivo* than *in vitro*, indicating that the conditions within the sand fly's gut are crucial to promote hybridization. Our research focuses on the study of Gex1, a protein recognized in other organisms for its role in nuclear fusion, via an approach combining genome editing, sand fly infections and the generation and use of specific polyclonal antibodies.
Using CRISPR/Cas9, we engineered *Leishmania* Gex1 *null* mutant and addback strains. While the wild type and addback controls successfully produce *in vitro* hybrids, Gex1 mutants failed to generate any, showing that Gex1 function is required for *Leishmania* sexual reproduction *in vitro*. In asymmetrical crosses - where only one parent expresses Gex1 - hybrid frequency strongly decreases but is not abolished, indicating that Gex1 is required in only one parental cell. We are currently characterizing the phenotype of these parasites and performing *in vivo* assays to test their mating competence in the sand fly. Additionally, we generated antibodies targeting different domains of Gex1, with the aim to track the protein expression and to identify key interaction partners.
Altogether, we demonstrated that Gex1 is essential in *Leishmania* hybridization and developed tools that will allow us to elucidate its mechanism of action *in vitro* and in the sand fly's gut.

Keywords: Vector/parasite interaction, *Leishmania*, Hybrids, CRISPR/Cas9

P_{17} On the taxonomic status of *Brumptomyia cavicola* Barreto, 1964 and *Brumptomyia dasypophila* Barreto, 1964 (Diptera, Psychodidae, Phlebotominae)

Galati A. B. Eunice[1]*, Rodrigues L. Bruno[1]

[1]*Public Health Scholl, São Paulo University. São Paulo, SP, Brazil*

***Corresponding author:** egalati@usp.br

Abstract

Brumptomyia cavicola and *Brumptomyia dasypophila* (*sic*.), sampled in Cássia dos Coqueiros municipality, SP-Brazil, were described by Barreto in an abstract published in "Ciência e Cultura,1964,6(2):156-157". *Brumptomyia cavicola* and *Br. dasipophyla* were considered close-related to *Br. troglodytes* (Lutz) and *Br. guimaraesi* (Barretto & Coutinho), respectively, from which both differ by their terminalia characters. Both were subsequently not included in any of sandfly classifications, but have been accepted as potentially species valid in the Catalog of Life. Here, we aimed to evaluate the taxonomic status of *Br. cavicola* and *Br. dasipophyla*. For this, we assessed the specimens on which Barreto's description was based, now deposited at the Museum of Zoology of USP. Four males (MZ006779-MZ006782) present one original label on their slides, having the type-locality (Coqueiros, SP), the collectors (Albertin and Barretto), and collection date, but not the species name. In addition, two slides (MZ006761 and MZ006762) were assigned as holotype and paratype of *Br. dasipophyla*, respectively, from the same locality. Comparing *Br. dasipophyla* specimens from MZUSP with others identified as *Br. cunhai* (Mangabeira) from eight Brazilian states of extra-Amazonian regions, we found no differences between them, however these specimens differ from those of *Br. cunhai* from Belém, Pará (type-locality) in the Amazon region, mainly regarding terminalia aspects. The characteristics of MZ006779-MZ006782 specimens coincide with those of *Br. cavicola*, and are similar to those of *Br. ortizi* Martins, Falcão & Silva, 1971. In conclusion, *Brumptomyia dasipophyla* is a valid species, and *Br. cavicola* is proposed as senior synonym of *Br. ortizi*.

Keywords: taxonomy, sand flies, synonyms

Funding: FAPESP - Fundação de Amparo à Pesquisa do Estado de São Paulo (n° 2023/03715-2) and Coordenação de Aperfeiçoamento de Pessoal de Nível Superior (CAPES) for scholarship grants to BLR — financial code 001

*P*18 MALDI-TOF mass spectrometry for species identification and blood meal determination of phlebotomine sand flies

Halada Petr[1]*, Hlavackova Kristyna[2], Kavan Daniel[1], Chaskopoulou Alexandra[3], Volf Petr[2], Dvorak Vit[2]

[1]*Institute of Microbiology of the Czech Academy of Sciences, BioCeV, Vestec, Czech Republic*
[2]*Department of Parasitology, Faculty of Science, Charles University, Prague, Czech Republic*
[3]*USDA–ARS, European Biological Control Laboratory, Thessaloniki, Greece*

***Corresponding author:** halada@biomed.cas.cz

Abstract

Leishmaniases caused by *Leishmania* protozoans and transmitted by females of phlebotomine sand flies (Diptera: Psychodidae) belong to very important yet still neglected vector-borne diseases. When evaluating the epidemiological role of sand flies, both reliable species identification and knowledge of trophic preferences towards reservoir hosts are required to understand local transmission cycles and design efficient disease control strategies in endemic regions.
During the last decade, two matrix-assisted laser-desorption/ionisation time of flight mass spectrometry-based methods, MALDI-TOF MS protein profiling and MALDI-TOF MS peptide mass mapping have been successfully employed in sand fly research. MALDI-TOF MS protein profiling was proved as a method of choice for conclusive, time and cost-effective species identification of large sets of field-caught sand flies from various endemic regions of the Old World including the Mediterranean, East Africa or Southeast Asia, creating a reference database that currently includes more than 40 sand fly species. Standardized protocol of specimen trapping, storage and sample preparation ensures to acquire reproducible species-specific protein profiles that serve as useful tool in integrative taxonomy, supporting formal description of new species, suggesting existence of yet undescribed cryptic species, discriminating among sibling species and challenging validity of established taxons while allowing utilization of a single sand fly specimen for other purposes (DNA barcoding, morphological analysis, blood meal identification, pathogen screening). MALDI-TOF MS peptide mass mapping of host-specific haemoglobin peptides in engorged females enables efficient and reliable blood meal origin identification, including mixed blood meals, up to 48 hours post feeding, clearly outperforming other currently used methods.

Keywords: MALDI-TOF mass spectrometry, species identification, blood meal determination

Funding: Czech Science Foundation (GA23-06299S)

P19 Vector competence of European sand flies to *Leishmania donovani, L. major* and *L. martiniquensis*

Hoskova Anna[1], Stejskalova Marketa[1], Vojtkova Barbora[1], Polanska Nikola[1], Jancarova Magdalena[1], Volf Petr1, Sadlova Jovana[1]*

[1]*Charles University, Prague, Czech Republic*

***Corresponding author:** sadlovaj@natur.cuni.cz

Abstract

Recently, several *Leishmania* species (*L. donovani*, *L. major* and *L. martiniquensis*) have emerged in Europe. Therefore, within the CLIMOS project, we aimed to test the vector competence of three European sand fly species, *P. perniciosus*, *P. tobbi* and *S. minuta*, for their transmission. Sand flies were allowed to feed on infected blood through the chick skin membrane, *Leishmania* infections were evaluated by light microscopy and qPCR and the representation of morphological forms was assessed from Giemsa-stained gut smears. Sand fly species were considered competent if *Leishmania* survived defecation of blood meal remnants, colonized the stomodeal valve and metacyclic stages developed in mature infections.

The results showed that *P. perniciosus* and *P. tobbi* support the development of late-stage infections of *L. major* and *L. donovani* and may be considered as potential vectors of these parasites in Europe. In contrast, *L. martiniquensis* failed to develop in these sand fly species. The vector competence of *S. minuta* remains unclear because females of this species have refused to take blood by membrane feeding, despite several types of membranes (chicken skin, synthetic membrane, pig intestine and reptilian skin), blood (ram, avian, reptilian and human) and experimental conditions were tested. Out of the eight other sand fly species used for comparison, 5 fed on all membrane types (*S. schwetzi*, *Lutzomyia migonei*, *P. arabicus*, *P. sergenti* and *P. argentipes*), *P. duboscqi* refused to feed through the artificial membrane and 2 species *(Lu. longipalpis* and *P. perniciosus)* refused to feed through the synthetic membrane and pig intestine.

Keywords: *Phlebotomus*, *Sergentomyia*, *Leishmania*, vector competence

Funding: The CLIMOS consortium is co-funded by the European Commission grant 101057690 and UKRI grants 10038150 and 10039289. CLIMOS is one of the six Horizon Europe projects, BlueAdapt, CATALYSE, CLIMOS, HIGH Horizons, IDAlert, and TRIGGER, forming the Climate Change and Health Cluster.

P20 Cloning and expression of the *wsp* gene encoding the main surface protein of *Wolbachia* endosymbiont of *Phlebotomus papatasi* (Diptera: Psychodidae)

Khanahmadi Nasim[1,2], Maleki-Ravasan Naseh[1*], Ajdary Soheila[3], Karimian Fateh[1], Heydarian Peyman[3], Babaie Jalal[1]

[1]*Department of Parasitology, Pasteur Institute of Iran, Tehran, Iran*
[2]*Department of Medical Parasitology and Mycology, School of Medicine, Qazvin University of Medical Sciences, Qazvin, Iran*
[3]*Department of Immunology, Pasteur Institute of Iran, Tehran, Iran*

***Corresponding author**: naseh_maleki@yahoo.com

Abstract

The lack of a vaccine and treatment failure in leishmaniasis offer many enthusiasms for research on details of the disease. Studies have shown that co-inoculation of *Leishmania major* with gut bacteria exacerbates the pathological responses of the vertebrate hosts, while pre-exposure of the hosts to sand fly bites confers significant protection against infection. The aim of this study was to produce a recombinant WSP protein of *Wolbachia* with the ultimate goal of accessing its antigenicity and immunogenicity in an animal model.
The sand flies *Phlebotomus papatasi* were captured from a *Lesishmania*-endemic focus in Iran during their activity seasons in June and July 2023. *Wolbachia* endobacteria were identified through dissection of the sand flies and amplification/sequencing of ~650-bp of *wsp* gene using universal primers. The *wsp* fragments were respectively inserted into *pTG* TA cloning and *pET21a* expression vectors.
The insertion of a 627-bp fragment of the *wsp* gene in the *pET-21a*, was confirmed by bioinformatic analysis. A 23.5 kDa rWSP was produced on both small and large scales. Small-scale expression parameters were optimized for large-scale protein production. Western blotting and SDS page were used to confirm the expression of the recombinant protein in both stages.
Wolbachia and its products are a promising target in leishmaniasis research due to their dynamic role in pathogen-host interactions. The successful expression and purification of the rWSP protein will be very useful to infer the protective role of bacteria transferred to the vertebrate host during the bite of sand fly vectors.

Keywords: *Phlebotomus papatasi*, Zoonotic Cutaneous Leishmaniosis, Wolbachia Surface Protein

P21 Hotspots of sandfly's abundance in Sicilian hinterland (10 years of sampling)

Di Pasquale M. Liliana[1], Vitale Fabrizio[3], Oliveri Eugenia[2], Scibetta Silvia[3], Reale Stefano[3], Facciponte Flavia[2], Giardina Susanna[2], Tumminia Silvia[2], Villari Sara M.[1], La Russa Francesco[1]*

[1]*Entomology and Environmental Vector Control Lab., Istituto Zooprofilattico Sperimentale della Sicilia A. Mirri, Palermo, Italy*
[2]*Centro di Referenza Nazionale per le Leishmaniosi (C.Re.Na.L.), Istituto Zooprofilattico Sperimentale della Sicilia A. Mirri, Palermo, Italy*
[3]*Istituto Zooprofilattico Sperimentale della Sicilia A. Mirri, Palermo, Italy*

***Corresponding author:** francesco.larussa@izssicilia.it

Abstract

According to the Piano Nazionale Arbovirosi (PNA), vectors are monitored, the purpose of the study is showing a hot spot of presence and abundance of phlebotomus in investigated areas. Sampling was conducted from January to December from 2010 to 2020, and black light traps were activated for one night from sunset to the next sunrise. Distinction and counting of sand flies from other insects were performed morphologically. The sites studied are located in the Sicilian hinterland provinces of Caltanissetta and Enna between 500-900 meters above sea level. The sampling sites consist in cattle/horse/sheep farms, environments are characterized by herbaceous plants or polyphytic meadows, dry stone walls, centuries-old olive groves and hay piles that certainly contribute to a perfect habitat for sand flies' proliferation; indoor environments are frequented by dogs. The raw soil rich in animal droppings maintains ideal conditions for immature stages's development; *Phlebotomous perfiliewii* and P. *perniciosus* are the species detected. The data obtained suggest the need to thoroughly investigate the sites' environmental conditions and compare them with human and canine leishmaniasis positive cases to have a more accurate picture of the circulation and transmission of the zoonosis in the region. The results are essential to implement data and create predictive models of phlebotomus population dynamics in Sicily, and investigation of favored habitat conditions (still lacking in the literature) will be useful for surveillance and prevention purposes to preserve human, animal and environmental health.

Keywords: hotspot, environment, One Health.

P22 Salivary gland extracts of *Phlebotomus papatasi* alter miRNA and inflammatory gene expression in Human monocytes-derived macrophages

Mkanenz Ghada[1], Guerfali Fatma Z.[1], Bali Aymen[1], Atri Chiraz[1], Laouini Dhafer[1]*

[1]*Laboratory of Transmission, Control and Immunobiology of Infections (LTCII) LR16IPT02, Institut Pasteur de Tunis, Tunisia*

***Corresponding author:** Dhafer.Laouini@gmail.com

Abstract

Micro-RNAs (miRNAs) are highly conserved between animals and plants, and numerous studies have shown their involvement in several physiological processes, such as cell differentiation, apoptosis, and metabolism in insects, neuronal development in nematodes, and the control of leaf and flower development in plants. During its blood meal, the vector of leishmaniasis inoculates parasites into the host's dermis along with saliva. It is well established that the salivary components of *Phlebotomus* (*P.*) *papatasi*, the vector of *Leishmania major* parasites, play a key role in protection or susceptibility during *Leishmania* infection. These components consist of a plethora of bioactive molecules that interfere with the host's response.

In the present study, we used a PCR array to investigate the effect of *P. papatasi* salivary gland extracts (SGE) on the expression of inflammatory genes and miRNA expression in human macrophages after 18 and 48 hours of treatment.

Among the tested miRNAs, only 15 and 6 were consistently over-expressed in macrophages after 18 and 48 hours of SGE treatment, respectively. Our results also showed that, among the tested inflammatory genes, 9 were up-regulated after salivary gland treatment. Most of these up-regulated genes encode chemokines and cytokines.

These findings provide evidence that the miRNA profile could serve as an early indicator of host cellular regulation induced by *P. papatasi* salivary gland components. Such modulation would likely influence the propagation of Leishmania, counteracting the immune response and aiding parasite survival within host cells.

Keywords: *Phlebotomus papatasi,* salivary gland extracts, human macrophages, miRNA, inflammation.

P23 Contribution to the sandflies of the Philippines

Loyer Mathieu[1]*, Nacri Mehdi[1], Randrianambinintsoa Fano[1], Huguenin Antoine[1], Gay Frédérick[2], Vongphayloth Khamsing[3], Depaquit Jérôme[1]

[1]UR7510 "ESCAPE" - USC ANSES "PETARD", Faculté de Pharmacie, Université de Reims Champagne Ardenne (URCA), Reims, France
[2]Sorbonne Université, INSERM, Institut Pierre Louis d'Epidémiologie et de Santé Publique, Hôpital Pitié Salpêtrière, APHP, Paris, France
[3]Laboratory of Vector-Borne Diseases, Institut Pasteur du Laos, Samsenhai Road, Ban Kao-Gnot, Sisattanak District, 3560 Vientiane, Lao PDR

***Corresponding author:** reyol.cobalt@gmail.com

Abstract

With 27 known species, the Philippines archipelago is a real Eldorado for faunistic, taxonomic and biogeographic research. After a pike of activity from the early twentieth century to the sixties, only two new species were described until nowadays. The mixed geographical origin (orogenesis and continental shifting) is promising for genera like the cavernicolous *Idiophlebotomus* and *Chinius*. This work summarises the state of the art about the known fauna of sandflies (27 species, *Phlebotomus philippinensis*, *Sergentomyia tracheolus*, *Sergentomyia yoshimotoi*, *Sergentomyia heiseri*, *Sergentomyia lagunensis*, *Sergentomyia dentaceus*, *Sergentomyia dapsilidentes*, *Sergentomyia manganus*, *Sergentomyia bigtii*, *Sergentomyia torrechantei*, *Sergentomyia spinifaucis*, *Sergentomyia nicnic*, *Sergentomyia neras*, *Sergentomyia hitchensi*, *Sergentomyia losarcus*, *Sergentomyia delfinadoae*, *Sergentomyia exastis*, *Sergentomyia dayapensis*, *Sergentomyia franciscanus*, *Sergentomyia bukidnonis*, *Sergentomyia imitor*, *Idiophlebotomus stellae*, *Idiophlebotomus sejunctus*, *Idiophlebotomus erebicolus*, *Idiophlebotomus pholetor*, *Idiophlebotomus padillarum*, *Chinius samarensis* described from Luzon, Mindanao, Palawan, Negros, Cebu and Samar) and discusses the creation of several new species for science. We also emphasis on the taxonomy of some *Idiophlebotomus*, like *Idiophlebotomus pholetor* (Quate & Fairchild, 1961, Borneo), mentioned in Palawan (Eran Area) by Quate (1965) and later found by Gay (2012) in another location in Palawan.

Keywords: Philippines, cavernicolous, *Idiophlebotomus*, Cytochrome b mt DNA, new species

P24 Sand flies in holiday paradise: first data on sand fly fauna of Phuket Island, Thailand

Mandysova Adela[1]*, Siriyasatien Padet[2], Halada Petr[3], Sunantaraporn Sakone[2], Boonserm Rungfar[2], Volf Petr[1], Dvorak Vit[1]

[1]Department of Parasitology, Faculty of Science, Charles University, Prague, Czech Republic
[2]Department of Parasitology, Faculty of Medicine, Chulalongkorn University, Bangkok, Thailand
[3]Institute of Microbiology of the Czech Academy of Sciences, BioCeV, Vestec, Czech Republic

***Corresponding author:** mandysoad@natur.cuni.cz

Abstract

In Southeast Asia, Thailand reports the highest numbers of autochthonous human leishmaniasis caused by *Leishmania martiniquensis* and *L. orientalis*. Therefore, extensive entomological studies of sand fly fauna across the country are conducted to identify its potential vectors. Despite its status of a popular tourist destination, the sand fly fauna of Phuket Island in Thailand remains undocumented. The aim of this study was to assess the species composition of local sand flies and determine the molecular prevalence of trypanosomatids of Phuket Island.
In total, 396 sand fly specimens were collected at three different localities with various habitats. Eight species belonging to genera *Sergentomyia*, *Phlebotomus* and *Grassomyia*, were identified. The species identification based on morphological analysis was further confirmed by sequencing of cytochrome oxidase I and cytochrome B genes. Additionally, first protein spectra of Thai sand fly species were obtained using MALDI-TOF protein profiling.
The most prevalent species found was *Ph. stantoni*, followed by *Se. khawi* and *Se. hivernus/iyengari*. Specimens of *Sergentomyia* sp. which could not be assigned to any described species were collected. Sand fly females were also screened for *Leishmania* spp., *Trypanosoma* spp. and *Wolbachia* spp. One specimen was tested positive for *Trypanosoma* sp. DNA, but all tested samples were negative for *Leishmania* spp. or *Wolbachia* spp.
This study provided first data about sand fly fauna of Phuket and screening of sand fly-borne pathogens.

Keywords: sand flies, Phuket, Thailand, species identification

P_{25} Sandflies longitudinal epidemiological study in the upper central part of Italy (2019-2021)

Mangiapelo Claudia[1], Bernardini Ilaria[1], Bianchi Riccardo[1], Fiorentino Eleonora[1], Foxi Cipriano[2], Mosca Andrea[3], Calzolari Mattia[4], De Liberato Claudio[5], Di Muccio Trentina[1], Satta Giuseppe[2], Gradoni Luigi[1], Bongiorno Gioia[1]*, Angelini Paola[6]

[1]*Department of Infectious Diseases, Unit of Vector-Borne Diseases, Istituto Superiore Di Sanità, Rome, Italy*
[2]*Istituto Zooprofilattico Sperimentale Della Sardegna, Sassari*
[3]*Istituto Per Le Piante Da Legno E L'ambiente - Ipla S.P.A, Turin, Piedmont*
[4]*Istituto Zooprofilattico Sperimentale Della Lombardia e dell'Emilia Romagna, Brescia*
[5]*Istituto Zooprofilattico Sperimentale del Lazio e della Toscana, Rome*
[6]*Servizio Prevenzione Collettiva e Sanità Pubblica - Emilia-Romagna Region*

***Corresponding author:** gioia.bongiorno@iss.it

Abstract

Climate change is gradually leading sand flies, tiny blood-feeding insects, to expand their boundaries and seasonal activity. They are known vectors of zoonotic visceral and cutaneous leishmaniasis caused by *Leishmania infantum* and associated with Phleboviruses, such as the Toscana virus. This study aims to provide an epidemiological frame, using sandfly species distribution reported in four Italian regions.
A longitudinal study as part of West Nile virus surveillance was carried out from 2019 to 2021 in the upper central part of Italy: Piedmont, Emilia-Romagna, Latium and Sardinia. Samplings were performed twice a month, using CDC and BG-sentinel traps. Subsamples were morphologically identified and molecularly tested by RFLP and RT-PCR for pathogens detection.
On a total of 96747 collected specimens 9000 were morphologically identified, belonging to four species: *Phlebotomus perfiliewi* (81.1%), *Ph. perniciosus* (14.9%), *Sergentomyia minuta* (3.7%) and *Ph. mascittii* (0.3%). In Piedmont *Ph. perniciosus* was the prevalent species while Emilia Romagna reported 99.6% *Ph. perfiliewi* prevalence. Lazio showed 99.7% of *Ph. perfiliewi* and 0.3% of *Ph. perniciosus*. Sardinia had a 61.8% prevalence of *Ph. perniciosus*, followed by *S. minuta* (20.3%) and *Ph. perfiliewi* (17.9%). Molecular analyses to detect *Leishmania spp*. on a total of 4062 specimens showed a positivity rate in Piedmont (11.2%) and Sardinia (11.1%). Toscana Virus analyses on a total of 3321 specimens revealed a positivity in Latium (0.4%), Piedmont (1.7%) and Sardinia (0.5%).
These preliminary results confirm the ubiquitous presence of *Ph. perniciosus* and *Ph. perfiliewi* as the most abundant species and a fair circulation of aforementioned pathogens.

Keywords: sand flies, climate change, sand-fly borne diseases

P26 Educational series for children based on sandflies as vectors of *Leishmania*

Milagres Tarcísio[1,2,3]*, Aparicio Lola[4], Barón Jorge[4], Gálvez Rosa[1,4]

[1]*Flebocollect Medical Entomology Citizen Science Group, Madrid, Spain.*
[2]*Department of Microbiology, Immunology and Parasitology, Universidade Federal Do Rio Grande do Sul, Porto Alegre, Brazil.*
[3]*Instituto de Higiene e Medicina Tropical, Universidade NOVA de Lisboa, Lisboa, Portugal*
[4]*Department of Specific Didactics, School of Education and Teacher Training, Universidad Autónoma de Madrid, Madrid, Spain.*

***Corresponding author:** tarcisiodefreitasmilagres@gmail.com

Abstract

This book series was created as an educational and informative tool, providing an accessible and engaging introduction to the world of sandflies and the importance of understanding these insects that cause a great impact on human and animal health. Although it is primarily aimed at children under 12 years old, the target audience can also be the general public, as this series presents a scientific and technological divulgation discourse. The educational material is crafted with language tailored to young readers, promoting the dissemination of information about sandflies and filling an educational gap targeted at this age group. Simultaneously, it aims to spark children's interest in science and the environment, encouraging curiosity and scientific literacy. The language of the books is structured based on didactic studies, and through simple and unpretentious texts, the stories in this series introduce relevant information in a playful manner through illustrations and games, making the educational process functional and intuitive. Series are already translated into five different languages: English, Spanish, Italian, Greek, French and Portuguese. Currently, there are three books in the series: "The Sand Fly," which focuses on the basic characteristics of this insect, such as size, reproduction, habits, and biological development; "The Sand Fly and the Parasite," which delves into the concept of the sand fly as a vector of the protozoan parasite Leishmania, providing information on parasite-vector interaction, clinical manifestations, and disease prevention; and "The Sand Fly Travels the World," which features the main character traveling to various countries and informing the reader about the epidemiological situation of leishmaniasis in different regions, addressing how climate and social changes are linked to this disease.

Keywords: sand fly, *Leishmania*, science communication, science education, leishmaniasis

P27 Entomological Survey of sand flies in an intense area for visceral leishmaniasis in Maranhão state, Brazil

Jucelei Infran O.M.[1,2], Casaril Aline E.[1], Barbosa Matheus E.P.[2], Fernandes Wagner S.[1], Saraiva Elanderson F.[3], Galati Eunice A.B.[4], Galvis-Ovallos Fredy[4], Werneck Guilherme L.[5], Cruz Maria do Socorro P.[6], Silva Monique Pinheiro Maia[7], Oliveira Alessandra G.[1,2,4]*

[1]*Institute of Biosciences Federal, University of Mato Grosso do Sul, Brazil*
[2]*Graduate Program in Infectious and Parasitic Diseases, Faculty of Medicine, Federal University of Mato Grosso do Sul, Brazil*
[3]*Institute of Mathematics, Federal University of Mato Grosso do Sul, Brazil*
[4]*Faculty of Public Health, University of São Paulo, Brazil*
[5]*Institute of Public Health Studies, Federal University of Rio de Janeiro, Brazil*
[6]*Graduate Program of Applied Technologies to Animals of Regional Interest, Federal University of Piauí, Brazil*
[7]*Health Secretary of Maranhão State, Brazil*

***Corresponding author:** alessandra.oliveira@ufms.br

Abstract

In the Americas, Brazil is responsible for 96% of the visceral leishmaniasis cases notified and it is estimated that they are concentrated mainly in 121 municipalities, mainly in the states of Maranhão, Pará, and Tocantins. In the Maranhão state, the Ministry of Health considers Caxias municipality a priority area for prevention and control measures. The study's objective was to characterize the sand fly fauna in the urban area of Caxias. The sand flies were collected over a three-night period between August 2021 and August 2023. The collection was conducted using automatic light traps (CDC-type) in both indoor and outdoor areas of 20 residences, from 6:00 pm to 6:00 am. A total of 103,350 sandflies belonging to 23 species were identified. The following species were identified: *Bichromomyia flaviscutellata*, *Brumptomyia avellari*, *Br. brumpti*, *Evandromyia begonae*, *Ev. bourrouli*, *Ev. evandroi*, *Ev. lenti*, *Ev. sallesi*, *Ev. saulensis*, *Ev. termitophila*, *Lutzomyia evangelistai*, *Lu. longipalpis*, *Lu. sherlocki*, *Micropygomyia longipennis*, *Mi. trinidadensis*, *Migonemyia villelai*, *Nyssomyia antunesi, Ny. whitmani*, *Psathyromyia aragaoi*, *Pa. bigeniculata*, *Pa. campbelli*, *Pa. hermanlenti*, and *Sciopemyia sordellii*. The peripheral areas close to the forest edge had a higher number of species captured (n=24), while in the central area, the presence of 15 species was identified. The most abundant species was *Lutzomyia longipalpis*, followed by *Ny. whitmani*. Both species have medical significance as vectors of the etiological agents of VL and cutaneous leishmaniasis, respectively. It is strongly recommended that health surveillance and control efforts in the municipality be intensified.

Keywords: Phlebotomines, Caxias, *Lutzomyia longipalpis*, Leishmaniasis

P_{28} Description of a new species of the Saulensis series, *Evandromyia* genus (Diptera, Psychodidae, Phlebotominae)

Oliveira G. Alessandra[1,2]*, Galvis-Ovallos Fredy[1], Rodrigues L. Bruno[1], Galati Eunice A.B.[1]

[1]*Faculty of Public Health, University of São Paulo, Brazil*
[2]*Institute of Biosciences, Federal University of Mato Grosso do Sul, Brazil*

***Corresponding author:** alessandra.oliveira@ufms.br

Abstract

Evandromyia saulensis (Floch & Abonnenc) and *Ev. wilsoni* (Damasceno & Causey) constitute the Saulensis series of the *Evandromyia* subgenus. The main diagnostic male character is the paramere with two elbows on the ventral margin, and for females, the spermathecae resemble a small bunch of grapes. The differentiation between these species primarily relies on the male genitalia. Both species were initially described from males, *Ev. saulensis* from French Guiana, and *Ev. wilsoni*, Amazonas state, Brazil. *Ev. saulensis* has been reported from different biomes: Amazonian, Cerrado, and Caatinga, and frequently found in caves. Our group has observed variations in certain morphological traits among specimens identified as *Ev. saulensis* from different biomes. The main goal of this study was to investigate specimens of both sexes collected in two Brazilian biomes: the Amazonian (forest and caves in Pará state) and the Cerrado (Pitoco's cave, Alcinópolis, Mato Grosso do Sul state). The Amazonian specimens were identified as belonging to *Ev. saulensis*, while those from the Cerrado, to a new species. Males of this new species can be distinguished from those of *Ev. saulensis* by the paramere, which presents a closer distance between the two elbows and distinct briste covering than those of *Ev. saulensis*. The new species also has a clypeus longer than the eyes, contrasting with *Ev. saulensis*, with clypeus shorter than eyes. This character is also evident in females, that display narrower spermathecae and spermathecal common duct than *Ev. saulensis*. Consequently, specimens previously identified as *Ev. saulensis*, particularly those from regions outside the Amazon, require further examination.

Keywords: *Evandromyia (Evandromyia*) sp. nov, cave, Mato Grosso do Sul

P_{29} Differentiation of females of *Lutzomyia cruzi* and *Lutzomyia longipalpis* (Diptera: Psychodidae: Phlebotominae) by infrared spectroscopy

Barbosa E.P. Matheus[1], Lacerda O. Miller [2], Infran O.M. Jucelei[1,3], Da Silva F. Thiago[2], Calvani C. Camila[2], Casaril E. Aline[3], Cena Cicero[2], Oliveira G. Alessandra[1,3,4*]

[1]*Graduate Program in Infectious and Parasitic Diseases - Faculty of Medicine - Federal University of Mato Grosso do Sul, Brazil*
[2]*Institute of Physics, Federal University of Mato Grosso do Sul, Brazil*
[3]*Institute of Biosciences, Federal University of Mato Grosso do Sul, Brazil*
[4]*Faculty of Public Health, University of São Paulo, Brazil*

***Corresponding author:** alessandra.oliveira@ufms.br

Abstract

Lutzomyia longipalpis and *Lutzomyia cruzi* are vectors of *Leishmania infantum* in Brazil. While *Lutzomyia longipalpis* is distributed throughout the country, *Lutzomyia cruzi* is confined to the Centre-West and Northeast regions. The morphological similarity of the females of both species presents a challenge for differentiation. In Mato Grosso do Sul, they are found in sympatry in six municipalities with cases of visceral leishmaniasis. The *longipalpis* complex, comprising *Lutzomyia longipalpis* and related species, has been delineated based on behavioral, biochemical, and morphological characteristics. To overcome this identification challenge, alternative methods are necessary. The main goal of the study was zo differentiate females of *Lutzomyia longipalpis* and *Lutzomyia cruzi* using Fourier Transform Infrared Spectroscopy (FTIR) combined with machine learning. 120 female sand flies, in groups of four, comprising 60 *Lutzomyia cruzi* and 60 *Lutzomyia longipalpis*, were analyzed. The acquired spectra underwent multi-analysis and machine-learning techniques for species characterization. The Linear Support Vector Machine (SVM) algorithm was employed across three band ranges (4000 to 600 cm^{-1}; 3000 to 2800 cm^{-1}; 1800 to 800 cm^{-1}) with principal components determined for each range. It was observed that any spectral range could yield a robust predictive model suitable for laboratory routines. Validation tests yielded a remarkable overall accuracy of 100% for all spectral ranges analyzed when appropriate principal components were chosen. Vibrational bands at 2800 cm^{-1} (lipids and fatty acids) and 1154, 1109 cm^{-1} (carbohydrates) were identified as key differentiators between the two species. The combination of FTIR and machine learning proves to be an effective approach for distinguishing between *Lutzomyia longipalpis* and *Lutzomyia cruzi*.

Keywords: FTIR, sandflies, females differentiation

P30 *Lutzomyia longipalpis* in intense and very intense transmission areas of visceral leishmaniasis in the Northeast and Central-West regions of Brazil

Infran O.M. Jucelei[1,2], Casaril E. Aline[1], Barbosa E.P. Matheus[2], Fernandes S. Wagner[1], Saraiva F. Elanderson[3], Monique Pinheiro Maia Silva[4], Galati A.B. Eunice[5], Galvis-Ovallos Fredy[5], Werneck L. Guilherme[6], Cruz P. Maria do Socorro[7], Oliveira G. Alessandra[1,2,5]*

[1]*Institute of Biosciences Federal, University of Mato Grosso do Sul, Brazil*
[2]*Graduate Program in Infectious and Parasitic Diseases, Faculty of Medicine, Federal University of Mato Grosso do Sul, Brazil*
[3]*Institute of Mathematics, Federal University of Mato Grosso do Sul, Brazil*
[4]*Health Secretary of Maranhão State, Brazil*
[5]*Epidemiology department, Faculty of Public Health, University of São Paulo, Brazil*
[6]*Institute of Public Health Studies, Federal University of Rio de Janeiro, Brazil*
[7]*Graduate Program of Applied Technologies to Animals of Regional Interest, Federal University of Piauí, Brazil*

***Corresponding author:** alessandra.oliveira@ufms.br

Abstract

In the Americas, *Lutzomyia longipalpis* is the main vector of *Leishmania infantum*. Our study aimed to quantify the fauna of *Lu. longipalpis* in the urban areas of Campo Grande (MS) and São Luís (MA), both classified as intense and very intense VL transmission areas, respectively. Collection efforts were conducted over three consecutive nights between August 2021 and August 2023 in São Luis and from July 2021 to August 2023 in Campo Grande with automatic light traps (CDC-type) in indoor and outdoor areas of 10 residences per city, from 6:00 p.m. to 6:00 a.m. *Lutzomyia longipalpis* was captured in all months and sampling points in Campo Grande, with 1,082 specimens (785 males and 297 females). Of these, 52% were captured indoors and 48% outdoors. The highest number of sandflies in the intra-domicile was likely due to the presence of a dog, where we observed the highest concentration of these dipterans. In São Luis, a total of 3,059 *Lu. longipalpis* were captured (2,182 males and 877 females). Of these, 13% were indoor and 87% outdoor. Interestingly, with the same sampling effort in the areas, differences in the densities of *Lutzomyia longipalpis* between the areas were observed. It could be explained by the ecological characteristics of the areas located in different biomes. The species has adapted to the anthropic environment, especially in areas with an abundance of organic matter, chicken coops, pig pens, and domestic dogs. In these environments, sandflies find ideal breeding, shelter, and food sites. These characteristics may justify the large number of specimens in all sampling points in both cities since many areas present an environment for the development of these dipterans.

Keywords: *Lutzomyia longipalpis*, Phlebotomine, visceral leishmaniasis, Brazil

P31 Effect of Urbanization on the Dominance of *Lutzomyia longipalpis* in Campo Grande, Mato Grosso do Sul, Brazil

Souza N. Rebeccah[1]*, Santos R. O. Luiz Gustavo[1], Oliveira G. Alessandra[1,2]

[1]*Graduate Program in Ecology and Conservation - Institute of Biosciences - Federal University of Mato Grosso do Sul, Brazil*
[2]*Graduate Program in Infectious and Parasitic Diseases - Faculty of Medicine - Federal University of Mato Grosso do Sul, Brazil*

***Corresponding author:** rebeccah.nogueira@ufms.br

Abstract

The lack of urban planning contributes to the adaptation of vectors in urban centers, spreading diseases such as visceral leishmaniasis, an endemic disease in Brazil, with *Lutzomyia longipalpis* as one of its main vectors. In Campo Grande, an endemic area for leishmaniasis, there are gaps in the study of the effect of urbanization on the dominance of *Lu. longipalpis*. Main goal of the study was to identify how the dominance of *Lu. longipalpis* behaves in response to changes in the urban landscape of Campo Grande, MS, from 1999 to 2024. Sixteen light traps were installed in 16 neighborhoods of Campo Grande, MS, Brazil. We identified all captured sand flies, analyzing the points with more than four sand flies collected in at least four sample years. We used software to obtain land use data and the percentage of urbanization around the collection points. To analyze the influence of urbanization on the dominance of *Lu. longipalpis*, we developed partial ordinary differential equations (ODEp) hypothesizing the relationships in which changes in urbanization over time (U/Δt) affect the quality of available urban habitat (K/Δt), determining vector dominance (N/Δt). Urbanization is positively related to the dominance of *Lu. longipalpis* and the intrinsic population growth rate. The urban growth rate directly influences the carrying capacity of the *Lu. longipalpis* population and its dominance. This may be associated with inadequate land use, poor sanitation, and other factors present in the urbanization process, facilitating the adaptation of vectors to the urban environment.

Keywords: dominance, sandflies, urbanization, Campo Grande, Brazil

P32 The Investigation of Epidemiology of Leishmaniasis in Bodrum Peninsula

Sevil Naser[1], Töz Seray[1], Demir Samiye[2], Özbel Yusuf[1]*

[1]*Department of Parasitology, Faculty of Medicine Ege University, Bornova, İzmir, Türkiye*
[2]*Department of Zoology, Faculty of Science, Ege University, Bornova, İzmir, Türkiye*

***Corresponding author:** yusuf.ozbel@ege.edu.tr

Abstract

The aim of this study was to carry out serological/parasitological survey on canine leishmaniasis and entomological survey for detecting sand fly species in Bodrum Peninsula located in southwestern of Türkiye where human cutaneous/visceral and canine leishmaniasis cases were previously reported.
Blood samples were collected into sera tubes and EDTA tubes from 124 dogs living in two dog shelters and 3 villages, and lymph node aspiration was also performed from 10 dogs with lymphadenopathy for parasite isolation. IFAT using sera samples and 13A/13B conventional PCR using blood and buffy coats were performed. The peninsula was divided into sections (8x8 km^2 grids) and at least one locality was selected from each section for sand fly collection.
Fifteen dogs out of 124 were found to be positive with at least one of the tests and canine leishmaniasis mean prevalance was determined as 12.09% in the peninsula.
A total of 985 sand fly specimens (10 *Phlebotomus* and 3 *Sergentomyia* species) were collected by CDC Light Traps from 50 different localities and identification results were as follows: *P. papatasi, P. alexandri, P. sergenti s.l., P. jacusieli, P. brevis, P. simici, P. tobbi, P. neglectus, P. mascittii, P. perfiliewi*, *Sergentomyia theodori*, *S. minuta* ve *S. dentata*. *P. tobbi* (35.93%), the proven vector of visceral leishmaniasis and *P. sergenti s.l.* (10.15%), probable vector of cutaneous leishmaniasis were found to be dominant species. The assessment of the results indicated that *P. tobbi* was predominant and probably the vector of CanL in the region.

Keywords: *Leishmania infantum*; prevalence, epidemiology; leishmaniasis, PCR, IFAT

P33 Comparative analysis of *Leishmania mexicana* amastigotes derived from different sources

Pacáková Lenka[1]*, Harant Karel[2,3], Kolářová Iva[1], Volf Petr[1], Leštinová Tereza[1]

[1]*Department of Parasitology, Faculty of Science, Charles University, Prague, Czech Republic*
[2]*Biotechnology and Biomedicine Centre of the Academy of Sciences and Charles University (BIOCEV), Vestec u Prahy, Czech Republic*
[3]*Proteomics Core Facility, Faculty of Science, Charles University, Prague, Czech Republic*

***Corresponding author:** lenka.pacakova@natur.cuni.cz

Abstract

Within the mammalian host, *Leishmania* parasites persist as intracellular amastigotes, characterized by a round shape and a short flagellum non exceeding the flagellar pocket. Previously, two alternative methods for studying amastigotes have been developed: cultivating axenic amastigotes by mimicking host macrophage conditions (temperature, low pH) or isolating amastigotes from *in vitro* infected macrophages. Both methods serve as substitutes for host-derived amastigotes and offer several advantages, however, their experimental use faces criticism for uncertain resemblance to lesion-derived amastigotes due to missing macrophage and host immune pressure. In this study, we compared axenic, macrophage-derived and lesion-derived amastigotes using various methods. We analyzed (1) the development of amastigotes in *Lutzomyia longipalpis,* (2) performed quantitative proteomic analysis focusing on major metabolic pathways, known virulence factors, and other important functional groups, and (3) compared antigens recognized by positive mice sera on immunoblot. In terms of development in sand flies, we observed significantly lower infection intensity in axenic amastigotes compared to other types. Significant differences were observed in the abundance of metabolic enzymes, virulence factors, and proteins involved in the translation and condensation of DNA. The most pronounced differences were found between axenic and lesion-derived amastigotes. Western blot analysis showed that anti-*Leishmania* antibodies did not bind to several bands of axenic amastigotes compared to other two types of amastigotes. Observed differences among amastigotes indicates that alternative amastigote forms may not be suitable for all types of experiments.

Keywords: *Leishmania*, amastigotes, sand fly vector, proteome, Western blot

Funding: The study was funded by Ministry of Education of the Czech Republic through the ERD Fund, project CePaViP (project CZ.02.1.01/0.0/0.0/16_019/0000759) and by Research center UNCE (project no. 204072).

P34 Natural *Wolbachia* infection in phlebotomine sandflies collected from tourist caves in Chiang Rai province, northern Thailand

Preativatanyou Kanok[1,2]*, Khositharattanakool Pathamet[3,4], Promrangsee Chulaluk[5], Siriyasatien Padet[1,2], Somwang Puckavadee[3,4]

[1]*Center of Excellence in Vector Biology and Vector-Borne Disease, Faculty of Medicine, Chulalongkorn University, Bangkok 10330, Thailand*
[2]*Department of Parasitology, Faculty of Medicine, Chulalongkorn University, Bangkok 10330, Thailand*
[3]*Biomedical Technology Research Group for Vulnerable Populations, Mae Fah Luang University, Chiang Rai 57100, Thailand*
[4]*School of Medicine, Mae Fah Luang University, Chiang Rai 57100, Thailand*
[5]*Interdisciplinary Program of Biomedical Sciences, Graduate School, Chulalongkorn University, Bangkok 10330, Thailand*

***Corresponding author:** kanok.pr@chula.ac.th

Abstract

Leishmaniasis has been known to be transmitted by phlebotomine sand flies. Of concern, autochthonous leishmaniasis cases have been increasingly reported in Thailand in recent years. One of the vector control strategies recommended is using genetically manipulated endosymbiotic *Wolbachia* bacteria, to express transgenic effects for suppressing pathogen multiplication and transmission. However, the genetic information of *Wolbachia* in Thai sand flies remains unknown. In this work, we investigated the prevalence of *Wolbachia* infection in sand flies captured from three tourist caves, namely Tham Phra Cave, Pha Thong Cave, Pha Jom Cave, in Chiang Rai Province, Northern Thailand. Ninety-two sand flies from four genera, namely *Sergentomyia*, *Phlebotomus*, *Grassomyia*, and *Idiophlebotomus*, were collected and screened for *Wolbachia* using *Wolbachia* surface protein gene (*wsp*)-specific PCR and direct Sanger sequencing. *Wolbachia* tested positive in 62 samples of all species except *Idiophlebotomus* sp., representing 67.4%. The identified *Wolbachia* sequences were phylogenetically classified into supergroups A and B and were highly identical to those detected in other dipterans such as *Armigeres* spp. (supergroup A) and *Culex quinquefasciatus* (supergroup B). It was found that a population of a single sand fly species could be infected with one to four *Wolbachia* strains. In addition, different sand fly species were found to share a common *Wolbachia* strain, suggesting either a common ancestral origin or a horizontal transmission between host species. To the best of our knowledge, this study provides preliminary information that would be helpful for a deeper understanding of host-endosymbiont relationships and greatly facilitate the development of effective management and control of neglected sand fly-borne diseases.

Keywords: Sandflies, *Wolbachia*, Leishmaniasis, Tourist caves, Thailand

P35 Phlebotomine sandflies of Madagascar: an update

Randrianambinintsoa Fano J.[1]*, Tantely Luciano[2], Vincent Robert[3], Depaquit Jérôme[1]

[1]*Université de Reims Champagne-Ardenne, Faculté de Pharmacie, UR EpidémioSurveillance et Circulation de Parasites dans les Environnements (ESCAPE), and ANSES, USC Pathogènes-Environnement - Toxoplasme-Arthropodes- Réservoirs- bioDiversité (PETARD), Reims, France; Centre Hospitalo-Universitaire, Laboratoire de Parasitologie-Mycologie, 51092 Reims, France*
[2]*Médical Entomology Unit, Institut Pasteur de Madagascar, Ambatofotsikely – 101 Antananarivo, Madagascar*
[3]*UMR MIVEGEC, University of Montpellier-IRD-CNRS, Montpellier, France*

***Corresponding author:** fano-jose.randrianambinintsoa1@univ-reims.fr

Abstract

The Malagasy sandfly fauna includes 21 species grouped into three genera: *Phlebotomus*, *Sergentomyia*, and *Grassomyia*. In Madagascar, three genera are present on the island.
The genus *Phlebotomus* includes six species, all belonging to the subgenus *Madaphlebotomus*, endemic to Madagascar. All males share characteristics with the subgenus *Anaphlebotomus,* which contains six species: two in continental Africa and four in southeast Asia, whereas the unique spermathecae of Malagasy *Phlebotomus* showing an asymmetric spermathecae constitute, in our opinion, a synapomorphy.
The genus *Sergentomyia*, which is widespread across the Old World, appears not to be monophyletic. It is well represented in Madagascar, with 13 species divided into five subgenera and some ungrouped species. Four subgenera are endemic to the Malagasy Region: *Ranavalonomyia*, *Riouxomyia*, *Vattieromyia*, and *Trouilletomyia*. The fifth subgenus, *Rondanomyia*, is also recorded in Asia. Some species have not been assigned to any subgenus and fall in the category of ungrouped *Sergentomyia*.
At least two species of the genus *Grassomyia* have been recorded in Madagascar: *Gr. Squamipleuris* and *Gr. madagascariensis*. The presence of this non-endemic genus on the island is not surprising given its large distribution in the Old World, from west Africa to southeast Asia, but its systematics seem more complicated in light of molecular investigations.

Keywords: systematics, new species, *Phlebotomus*, *Sergentomyia*, *Grassomyia*, phylogeny

P36 DNA barcoding approach as a useful support to the entomological examination of wild-caught phlebotomine sand flies

Scibetta Silvia[1], Oliveri Eugenia[1], Tumminia Silvia[1], Piazza Carlotta[1], Di Pasquale Maria Liliana[1], Castelli Germano[1], Bruno Federica[1], La Russa Francesco[1], Vitale Fabrizio[1], Reale Stefano[1]*

[1]*IZSSi Istituto Zooprofilattico Sperimentale della Sicilia, Palermo,Italy*

***Corresponding author:** stefano.reale@izssicilia.it

Abstract

Phlebotomine sand flies (Diptera, Psychodidae) are recognized as vectors of multiple pathogens, however, only two genera, *Phlebotomus* and *Sergentomyia*, act as vectors of Leishmaniasis in Afro-Eurasia regions.
The sand fly species identification is crucial for elucidating the epidemiology of leishmaniasis and implementing vector control strategies in endemic regions. Courrently traditional classification of sand fly species is based on the validated morpholocogical examination, but everytime this kind of identification is difficultous or for epidemiological scale approach it can be complemented by DNA-based methodologies as the molecular barcode.
The mitochondrial gene cytochrome c oxidase subunit I (COI) is widely utilized as molecular marker in taxonomic identifications of various insect species, proving successful, also, in sand fly species. Therefore, DNA barcoding approach has emerged as a reliable molecular tool for species identification within traps used for monitoring insect dispersion.
This study aimed to assess the potential and the efficacy of COI sequence analysis for identifying prevalent sand fly species in Sicily, including those at risk of spreading: *Phlebotomus perniciosus, P. neglectus, P. papatasi, P. perfiliewi, P. ariasi*, *P. mascittii, P. tobbi* and *Sergentomyia minuta*.
The discriminative potential of the COI target in discriminate these species was assessed *in silico* by comparing sequences of 50 *Phlebotomus* species and 27 *Sergentomia* species obtained from NCBI. In a subsequent step, wild-caught sand flies circulating in Sicily, previously characterized by morphology, were subjected to DNA barcode analysis, encompassing DNA extraction, PCR/sequencing (Folmer primers), thus demonstrating the efficiency of the approach.

Keywords: Phlebotominae sand flies, identification, DNA-based methodologies

P37 Bioecological description of the Phlebotomine community in an area of the Southwestern Mesoregion of Pará state, Brazil: preliminary data

Rocha Goncala Elisene[1]*, Gomes A. Maurício[2], Silva F. C. Antonio[2], Galati A. B. Eunice[3], Oliveira G. Alessandra[1,2]

[1]*Graduate Program in Infectious and Parasitic Diseases - Faculty of Medicine - Federal University of Mato Grosso do Sul, Brazil*
[2]*Graduate Program in Ecology - Institute of Biosciences - Federal University of Mato Grosso do Sul, Brazil*
[3]*Faculty of Public Health, University of São Paulo, Brazil*

***Corresponding author:** elisene.rocha@ufms.br

Abstract

The intensification of migration and immigration flows can lead to environmental changes such as deforestation, mining activities and the construction of dams. These changes can favour the spread of cutaneous leishmaniasis (CL) in previously untouched regions. In the state of Pará, cases of cutaneous leishmaniasis (CL) have been reported in all 144 municipalities. Despite extensive research in Pará, Pinto et al. (2023) suggest that certain municipalities remain insufficiently studied, particularly those subject to constant environmental changes, such as Novo Progresso-PA. The main goal of our research was to describe the bioecological structure of the phlebotomine community in Novo Progresso-PA. 36 automatic light traps are placed every two months from 18:00 to 06:00 in different ecotopes, including urban, peri-urban, forest edge and forest interior areas surrounding the city. Trap locations were selected using the NDVI (Normalised Difference Vegetation Index) derived from satellite imagery. The trapped insects are identified and classified according to Galati (2023). 6,504 phlebotomines were collected, 2,924 males and 3,580 females were identified, with 4,739 successfully identified. The most prevalent species in forest and urban ecotopes are *Nyssomyia urbinattii*, *Nyssomyia antunesi* and *Psychodopygus davisi*. On the other hand, *Evandromyia walkeri*, *Trichophoromyia ubiquitalis* and *Psychodopygus complexus* are more prevalent in forest edges and interiors. Nyssomyia antunesi, Trichophoromyia ubiquitalis and *Psychodopygus complexus* were identified as potential vectors of *Leishmania* sp. The ecotones in the forest interior had the highest number and diversity of species.

Keywords: sandflies, landscape ecology, cutaneous leishmaniasis

P38 Molecular analysis of *Trypanosoma* parasite and *Bartonella* bacteria detected in cave-dwelling phlebotomine sandflies (Diptera: Psychodidae: Phlebotominae) collected from endemic areas of leishmaniasis in Thailand

Sunantaraporn Sakone[1], Khositharattanakool Pathamet[2,3], Somwang Puckavadee[2,3], Unchanam Isaraporn[4], Saenchaiban Nattiya[4], Wongkhut Wilai[4], Sanum Pinpinat[5], Boonserm Rungfar[1], Depaquit Jérôme[6], Siriyasatien Padet[1,7]*

[1]Center of Excellence in Vector Biology and Vector-Borne Diseases, Department of Parasitology, Faculty of Medicine, Chulalongkorn University, Bangkok 10330, Thailand
[2]School of Medicine, Mae Fah Luang University, Chiang Rai 57100, Thailand
[3]Biomedical Technology Research Group for Vulnerable Populations (BIOMED), Mae Fah Luang University, Chiang Rai 57100, Thailand
[4]The Office of Disease Prevention and Control 1, Chiang Mai 50000, Thailand
[5]Lampang Provincial Health Office, Lampang 52000, Thailand
[6]Faculté de Pharmacie, Université de Reims Champagne-Ardenne, SFR Cap Santé, EA7510 ESCAPE-USC ANSES VECPAR, Reims, France
[7]Department of Parasitology, Faculty of Medicine, Chulalongkorn University, Bangkok 10330, Thailand

***Corresponding author:** padet.s@chula.ac.th

Abstract

We collected sandflies from three caves located in endemic areas of leishmaniasis, including Lampang and Chiang Rai in the north and Songkhla in southern Thailand. A total of 557 female sandfly samples were screened for *Leishmania*, *Trypanosoma*, and *Bartonella* DNA by PCR based on the *ITS1* region of the rRNA, *SSU* rRNA, and *gltA* genes, respectively. Individual female sandflies were identified based on morphological characteristics and confirmed by cytochrome C oxidase subunit I (*COI*) sequencing. All sandflies in the current study were morphologically identified into twelve species, *Sergentomyia anodontis* was the dominant species found in Chiang Rai and Lampang, while *Se. khawi* was the most common species found in Songkhla. The results of molecular detection demonstrated that *Leishmania* DNA was not detected. However, *Trypanosoma* DNA was detected in 11 (1.97%) samples of *Phlebotomus mascomai* from Lampang (7 for *T. noyesi*), *Se. anodontis* from Chiang Rai (one for each *T. noyesi* and *Trypanosoma* sp.) and *Se. khawi* from Songkhla (2 for *Trypanosoma* sp.). We also detected *Bartonella* DNA in 16 (2.87%) samples of *Se. anodontis* and *Se. barraudi* from Chiang Rai, *Se. anodontis* from Lampang, and *Se. khawi* from Songkhla. The information data from the present study indicate that phlebotomine sandflies could be potential vectors of zoonotic diseases caused by *Trypanosoma* sp. and *Bartonella* sp. This is the first report of the natural infection of *Bartonella* associated with sandflies in Thailand and the presence of *Trypanosoma* parasites in the northern region of the country.

Keywords : Sandflies, *Trypanosoma* sp., *Bartonella* sp., Thailand

P39 The Epidemiological and Entomological Survey on Canine Leishmaniasis in Kırklareli Province Located in the Northwestern Part of Türkiye

Ertabaklar Hatice[1], Nalçaci Muhammed[2], Balcioğlu I. Cüneyt[3], Şakru Nermin[4], Özbel Yusuf[5], Demir Samiye[6], Töz Seray[5]*

[1]*Department of Parasitology, Medical Faculty, Aydın Adnan Menderes University Aydın, Türkiye*
[2]*Department of Zoology, Faculty of Science, Ege University, Bornova, İzmir, Türkiye*
[3]*Department of Parasitology, Faculty of Medicine, Manisa Celal Bayar University, Manisa, Türkiye*
[4]*Department of Microbiology and Clinical Microbiology Medical Faculty, Trakya University, Edirne, Türkiye*
[5]*Department of Parasitology, Faculty of Medicine Ege University, Bornova, İzmir, Türkiye*

***Corresponding author:** seray.ozensoy.toz@ege.edu.tr

Abstract

Human visceral leishmaniasis (HVL) and canine leishmaniasis (CanL) caused by *Leishmania infantum* are mainly observed in the Aegean and Mediterranean Regions of Türkiye while endemic or sporadic CanL cases have been reported from other provinces located in different geographical regions. This study aimed to carry out a serological survey on leishmaniasis among dog populations and an entomological survey on vector sand fly species in the villages of Kırklareli province where very limited information about CanL and its vectors are reported.
The province is divided into four geographical areas and three or four villages were selected from each one. In these villages, physical examinations and blood sampling were carried out on a total of 131 dogs. Anti-*Leishmania* antibodies were searched by Indirect Fluorescent Antibody Test (IFAT) by ≥1/128 titer was considered seropositive. To determine the vector sand fly species, 15 CDC Light traps and 290 A4-sized sticky papers were set up in seven villages.
Two dogs (1.51%) were found to be seropositive in two villages while 32 (24.42%) dogs were considered as borderline (in 1/64 titer). A total of 48 sand fly specimens were caught and among them; *Phlebotomus tobbi* (10.41%), *P. sergenti s.l.* (2.08%) and *Sergentomyia* sp. (87.7%) were found. These findings revealed that CanL cases and suitable vector species are present in this geographical area of Türkiye.

Keywords: Leishmaniasis, canine, epidemiology, sand fly, Kırklareli, Türkiye

P40 Diversity and Spatio-temporal from ground to the canopy of Phlebotomine Sand Flies (Diptera: Psychodidae) in the Ecuadorian Amazon Basin

Ramírez Sebastián[1], Torres-Sobrevilla Carla[1,2], Ulloa Catalina[2], Arroyo Santiago[2], Macanilla José[2], Randrianambininstoa Fano[3], Gantier Jean Charles[3], Depaquit Jérôme[3], Zapata Sonia[1,2]

[1]Instituto de Microbiología, Colegio de Ciencias Biológicas y Ambientales COCIBA, Universidad San Francisco de Quito USFQ, Quito, Ecuador.
[2]Estación de Biodiversidad Tiputini, Colegio de Ciencias Biológicas y Ambientales, Universidad San Francisco de Quito USFQ, Quito, Ecuador.
[3]EA7510 ESCAPE, ANSES, USC "Pathogènes-Environnement-Toxoplasme-Arthropodes-Réservoirs-bioDiversité (PETARD)", Université de Reims Champagne-Ardenne, Reims, France

***Corresponding author:** szapata@estud.usfq.edu.ec

Abstract

The Yasuní National Park is located in the Ecuadorian Amazon region, which is part of the world's largest biodiversity hotspot. Inside the park is the Tiputini Biodiversity Station, equipped with a 40-meter high tower for recording meteorological variables. This region reports cases of cutaneous and mucocutaneous leishmaniasis, but the vectors transmitting the disease and the reservoirs remain unknown. This study aimed to describe the fauna of sandflies in three different vertical strata on the tower from November 2022 to October 2023 to characterize their diversity, potential vectors, and reservoirs. Entomological collections were performed using CDC miniature light traps in the tower at three levels of altitude: ground, intermediate and canopy 1, 20 and 40 meters high respectively.
In total, 950 specimens were identified, belonging to the *Lutzomyiina* (108) and *Psychodopygina* (790) subtribes, with the latter being the most prevalent across all three strata. The overall male-to-female ratio was 1:1, although this ratio varied within the strata. At the ground level, the ratio was 1.72 males per female, at the intermediate level it was 0.70 males per female, and in the canopy, it was 0.87 males per female.
A greater abundance of sandflies was observed at the intermediate level, a pattern consistent for nine months, except in March, July, and August, when more sandflies were found at ground level. At the genus level, the most abundant were *Nyssomyia* (500), followed by *Psychodopygus* (202), *Lutzomyia* (45), *Psathyromyia* (38), *Pintomyia* (27), and *Trichopygomyia* (22). The remaining specimens were divided among *Trichophoromyia*, *Pressatia*, and *Sciopemyia*.
In total, 28 species of sandflies were recorded, with notable potential vectors of Leishmania being *Ps. panamensis*, *Pa. shannoni*, *Ps. ayrozai*, *Ps. carrerai carrerai*, *Ps. paraensis*, *Th. ubiquitalis*, *Lu. hartmanni*, *Ps. amazonensis*, and *Ny. yuilli*.

Keywords: sandflies, ground, canopy, vectors, Ecuadorian Amazon.

P41 Centrin gene-deleted *Leishmania major* vaccine protects against canine visceral leishmaniasis following repeated natural exposure in an endemic focus, Tunisia

Boussoffara Thouraya[1], Labidi Imen[2], Trimèche Malek[1], Chelbi Ifhem[1,2], Khalil Dachraoui[1,2], Abbas Mohammed[1,2], Cherni Saifedine[1,2], Singh Kamaleshwar P.[3], Kaviraj Swarnendu[3], Dey Ranadhir[4#], Varikuti Sanjay[5], Gannavaram Sreenivas[4#], Pereira Lais da S.[4#], Zhang Wen-Wei[6], Lypaczewski Patrick[6], Hamanno Shinjiro[7], Matlashewski Greg[6], Singh Sanjay[3], Louzir Hechmi[1], Nakhasi Hira L.[4#], Satoskar Abhay R.[5], and Zhioua Elyes[1,2]*

[1]Laboratory of Transmission, Control, and Immunobiology of Infections. Pasteur Institute of Tunis. University of Tunis El Manar, Tunis, Tunisia
[2]Unit of Vector Ecology, Pasteur Institute of Tunis, Tunis, Tunisia
[3]Gennova Biopharmaceuticals Limited, Hinjewadi Phase II, Pune, Maharashtra, India
[4]Division of Emerging and Transfusion Transmitted Diseases, Office of Blood Research and Review, Center for Biologics Evaluation and Research, Food and Drug Administration, Silver Spring, MD, USA
[5]The Ohio State University, Columbus, Ohio, USA
[6]Department of Microbiology and Immunology, McGill University, Montreal, QC, Canada
[7]Department of Parasitology, Institute of Tropical Medicine (NEKKEN), The Joint Usage/Research Center on Tropical Disease, Nagasaki University, Nagasaki, Japan
# Our contributions are an informal communication and represent our own best judgement. These comments do not bind or obligate FDA

***Corresponding author:** elyes.zhioua@gmail.com

Abstract:

Visceral leishmaniasis is a zoonosis disease, in which dogs are the major reservoir for *Leishmania infantum*. An effective vaccine against Canine Visceral Leishmaniasis (CVL) will help the control and elimination of VL. In this study, we evaluated in dogs the safety, immunogenicity, and efficacy of a live attenuated *Leishmania major* Centrin gene-deleted (*LmCen*$^{-/-}$) as a vaccine. Two doses (10^6 or 10^7) of *LmCen*$^{-/-}$ vaccine were administered intradermally in a prime-boost regimen. Both vaccine doses induced equally high level of IgG anti-*Leishmania* and exhibited strong antigen-specific cellular responses with IFN-γ production by CD4+T cells one-month post-immunization. A second cohort of dogs was vaccinated with 10^6 *LmCen*$^{-/-}$ parasites one month prior to their transfer to the endemic area for exposure to sand flies' bites during three successive transmission seasons. Dogs were exposed to bite from naturally infected sandflies for 3-5 months per year. Our results showed that only 1/11 vaccinated dogs became PCR positive for *Leishmania* and developed clinical signs of CVL. In contrast, 4/11 unvaccinated dogs were tested PCR positive for *Leishmania* and displayed oligosymptomatic CVL, demonstrating that immunization with *Lm*Cen$^{-/-}$ vaccine confers long-term protection with an efficacy of 82.5% against CVL in natural transmission settings.

Keywords: Canine leishmaniasis, dog vaccine, natural challenge

P42 Bioecological description of the Phlebotomine community in an area of the Southwestern Mesoregion of Pará state, Brazil: preliminary data

Rocha G. Elisene[1]*, Gomes A. Maurício[2], Silva F. C. Antonio[2], Galati A. B. Eunice[3], Oliveira G. Alessandra[1,2]

[1]*Graduate Program in Infectious and Parasitic Diseases - Faculty of Medicine - Federal University of Mato Grosso do Sul, Brazil*
[2]*Graduate Program in Ecology - Institute of Biosciences - Federal University of Mato Grosso do Sul, Brazil*
[3]*Faculty of Public Health, University of São Paulo, Brazil*

***Corresponding author:** elisene.rocha@ufms.br

Abstract

The intensification of migration and immigration flows can lead to environmental changes such as deforestation, mining activities and the construction of dams. These changes can favour the spread of cutaneous leishmaniasis (CL) in previously untouched regions. In the state of Pará, cases of cutaneous leishmaniasis (CL) have been reported in all 144 municipalities. Despite extensive research in Pará, Pinto et al. (2023) suggest that certain municipalities remain insufficiently studied, particularly those subject to constant environmental changes, such as Novo Progresso-PA. The main goal of our research was to describe the bioecological structure of the phlebotomine community in Novo Progresso-PA. 36 automatic light traps are placed every two months from 18:00 to 06:00 in different ecotopes, including urban, peri-urban, forest edge and forest interior areas surrounding the city. Trap locations were selected using the NDVI (Normalised Difference Vegetation Index) derived from satellite imagery. The trapped insects are identified and classified according to Galati (2023). 6,504 phlebotomines were collected, 2,924 males and 3,580 females were identified, with 4,739 successfully identified. The most prevalent species in forest and urban ecotopes are *Nyssomyia urbinattii*, *Nyssomyia antunesi* and *Psychodopygus davisi*. On the other hand, *Evandromyia walkeri*, *Trichophoromyia ubiquitalis* and *Psychodopygus complexus* are more prevalent in forest edges and interiors. *Nyssomyia antunesi*, *Trichophoromyia ubiquitalis* and *Psychodopygus complexus* were identified as potential vectors of *Leishmania* sp. The ecotones in the forest interior had the highest number and diversity of species.

Keywords: sandflies, landscape ecology, cutaneous leishmaniasis

EXTRAS

E1 Evaluation of the vector competence of *Migonemyia migonei* for *Leishmania* (*Viannia*) *braziliensis* and *Leishmania* (*Leishmania*) *infantum* through experimental transmission to susceptible hosts

Alexandre Joanna L.[1], Oliveira Debora E. [1], Silva Andrea K. F. [1], Almeida Ericka L. [1], Silva Fernando J.[1], Valença Hélio F., Shaw Jeffrey J.[2], Brandão-Filho Sinval P.[1]*

[1]*Department of Immunology, Aggeu Magalhães Institut, Oswaldo Cruz Foundation (Fiocruz), Recife, Pernambuco, Brazil.*
[2]*Department of Parasitology, ICB, University of São Paulo, São Paulo, Brazil.*

***Corresponding author:** sinval.brandao@fiocruz.br

Abstract

The Leishmaniases are important neglected tropical diseases. In the Americas, *Leishmania* (*Viannia*) *braziliensis* and *Leishmania* (*Leishmania*) *infantum* are respectively amongst the principal etiological agents of cutaneous and visceral leishmaniasis. The possibility of more than one sand fly species being involved in their enzootic and zoonotic cycles in an endemic area is a widely discussed subject. Natural infections of both parasites have been found in *Migonemyia migonei* in endemic areas. The incrimination of a sand fly species as a vector is based on a series of criteria that include the insect's ability to support the parasite's development after digestion of the blood meal. In addition to this a crucial step in evaluating a sand fly's vectorial competence is to demonstrate parasite transmission by its bite. Studies on the sand fly-*Leishmania* interaction are important and contribute to a better understanding of its transmission potential. The focus of our study is the experimental transmission of *L.* (*V.*) *braziliensis* and *L.* (*L.*) *infantum* by *Mg. migonei*. The *Mg. migonei* colony is maintained IAM/Fiocruz's insectary. Laboratory reared sand flies were infected with *L.* (*V.*) *braziliensis*, MHOM/BR/1975/M2903 (IOC-L 566 LRV) but unfortunately, the females did not survive to the experimental transmission stage. Presently the size of the colony is being increased to continue this work. The results will assist in confirming *Mg. migonei* vectorial competence for *L.* (*V.*) *braziliensis* and *L.* (*L.*) *infantum*. This information contributes to assessing more securely the risk of new leishmaniasis outbreaks involving this phlebotomine species as well as the development of effective leishmaniasis control and surveillance strategies.

Keywords: *Migonemyia migonei,* experimental transmission, vector competence.

Supported by PROEP/IAM/Fiotec (IAM-005-FIO-22) and CNPq (305607/2022-7) grants.

E2 Genetic variants in the genome of the *Lutzomyia longipalpis* complex

Pech-May Angélica[1,2*], Fraisse Christelle[2], Cigarroa-Toledo Nohemí[3], Moya Sofia Lorian[1,4] & Salomón Oscar Daniel[1,4]

[1]*Red de Investigación de la Leishmaniasis en Argentina (REDILA)*
[2]*Centro Regional de Investigación en Salud Pública, Instituto Nacional de Salud Pública, México*
[2]*University of Lille, Ecology and Evolution unit, France*
[3]*Centro de Investigaciones Regionales ''Dr. Hideyo Noguchi'', Universidad Autónoma de Yucatán, México*
[4]*Instituto Nacional de Medicina Tropical (INMeT), ANLIS "Dr. Carlos G Malbrán", Puerto Iguazú, Misiones, Argentina*

***Corresponding author:** apechmay@gmail.com

Abstract

Lutzyomia longipalpis is currently considered a species complex, despite a broad yet discontinuous distribution from Mexico to the north of Argentina and Uruguay. Currently, there are eight haplogroups with a high level of phylogeographic divergence. Three haplogroups have been found in Argentina. The present study aimed to identify SNPs and Indels of the *Lutzomyia longipalpis* complex: Haplogroup Ar1, Haplogroup Ar2 vs Haplogroup Bra. The specimens were collected in Puerto Iguazú and San Ignacio, Argentina using REDILA tramps. Genomic DNA was extracted from individual sandflies and the amplification of the ND4 gene and subsequently, the PCR products were purified for sequencing. This sequence was used for phylogeographic analysis by Bayesian inference to verify the haplogroup of *Lutzomyia longipalpis* specimens. Genome sequencing was performed on the Illumina platform ~64 million 150 bp reads per sample, the analysis of genome quality per sample was performed using FastQC Report, the programme 'SnpEff' was used to annotate the variants found among haplogroups and from the generated mapping files (BAMs), VarDict was used to perform variant calling. Four genomes were obtained: two from HgAr1 and two from HgAr2, which were mapped with the HgBra (Genbank: GCA_000265325). The genome length was approximately 154, 229, 266 nucleotides. The HgAr2 vs HgBra had the highest number of a) SNPs, b) number of insertions as well as deletions, and c) number of silent mutations. These findings suggest the need to analyze vector competence, according to vector haplogroup since *Lutzomia longipalpis* is the main vector of *Leishmania infantum*.

Keywords: *Lutzomyia longipalpis* complex, genome, SNPs, silent mutations

E₃ Diversity of Phlebotominae species in the Chaco region of Argentina

Rosa Juan[1,2]*, Gomez Gallipoliti Sofía[1,2], Ellena Miriam[1,2], Szelag Enrique[1,2,3], Quintana M. Gabriela[2,3,4,5], Salomon O Daniel[4]

[1]*Instituto de Medicina Regional. Universidad Nacional del Nordeste. Campus Resistencia, Chaco. Argentina.*
[2]*Red de investigación de la Leishmaniasis en Argentina (REDILA)*
[3]*Consejo Nacional de Investigaciones Científicas y Técnicas (CONICET)*
[4]*Instituto Nacional de Medicina Tropical (INMeT). Administración Nacional de Laboratorios e Institutos de Salud (ANLIS Malbrán). Puerto Iguazú, Misiones, Argentina*
[5]*Instituto Superior de Entomología (INSUE), Universidad Nacional de Tucumán. Argentina*

***Corresponding authors:** juan_rosa05@yahoo.com.ar

Abstract

Chaco province, Argentina (27°27'S-58°59'W), included the dry (DCh) and wet (WCh) Chaco ecoregion with cases and outbreaks of tegumentary (TL) and visceral leishmaniasis (VL).
In DCh (2021-2023) eight areas were selected with three sampling sites (S) in each one (domicile, peridomicile, forest). In the metropolitan area belonging to WCh (2022-2023), three sites in each urban, peri-urban, rural, and natural strata were selected. For 3 nights/month with 1 trap/site, CDC light traps (CDC-lt) in DCh and REDILA-BL traps, besides DCh 40 emergence traps (ET) and 84 organic substrate samples (OS) for immature states in WCh were used. Burrows (B) of *Lagostomus sp* (Rodentia: Chinchillidae) were studied as probable natural breeding sites using sticky traps (ST), CDC-lt and OS. The total phlebotomine obtained was 1421 (DCh) and 1190 (WCh): *Migonemyia migonei*: DCh 75%(ET/CDC-lt-B), 13.6%(ST); WCh (34%). Cortelezzii complex: 18.4%(ST), 14.4%(CDC-lt-B); WCh (27.4%). *Evandromyia sallesi*: 23.8%(ST), 6.1%(CDC-lt-B); WCh (6.2%). *Ev. cortelezzii* .4%(ST), 0.2%(CDC-lt-S); WCh (6.2%). Chacuensis complex: 13.4%(ST), 1.1%(CDC-lt-B). *Ev. chacuensis*: 19%(ST), 0.6%(CDC-lt-S/CDC-lt-B); WCh (0.1%). *Ev. cristacapita*: 14.3%(ST), 0.8%(CDC-lt-S); WCh (0.1%). *Nyssomyia neivai*: 2.5%(ET), 0.3%(CDC-lt-S); WCh (15.4%). Only in DCh *Ev. termitophila* 5.4%(ST), 0.1%(CDC-lt-S). *Pintomyia torresi* 0.7%(ST). In OS: *Mg. migonei* (1 female). Only in WCh *Lutzomyia longipalpis* (8%), *Sciopemyia sordellii* (0.1%), *Ev. aldafalcaoae* (1.6%), *Psathyromyia campograndensis* (0.1%). Common species could be considered as potential vectors due to abundance and background, *Lu. longipalpis* as primary vector of VL in WCh, and *Mg. migonei* in both as a primary/permissive vector of TL/VL. Burrows would be probable resting and/or breeding sites for sandflies in DCh.

Keywords: Sandflies - breeding site - Ecology - Vectors

E4

Current coverage of Phlebotominae DNA Barcodes in Digital Repositories: Update and Assessment for Argentina and the Americas

Moya Sofia L.[1,2,3]*, Quintana Maria G.[1,2,3,4], Liotta Domingo J.[1,2,5], Salomón Oscar D.[1,2,3]

[1] *National Institute of Tropical Medicine (INMeT), ANLIS "Dr. Carlos G Malbrán", Puerto Iguazú, Misiones, Argentina*
[2] *Research Network on Leishmaniasis in Argentina (REDILA), Argentina*
[3] *National Scientific and Technical Research Council (CONICET), Argentina*
[4] *Superior Institute of Entomology "Dr. Abraham Willink", San Miguel de Tucumán, Tucumán, Argentina*
[5] *Laboratory of Applied Molecular Biology (LaBiMAp-FCEQyN-UNaM), Posadas, Misiones, Argentina*

***Corresponding author**: sofialorian@gmail.com

Abstract

After 20 years since the introduction of the "barcode" concept, its potential as a tool for species identification and integrative taxonomic hypotheses remains significant. However, understanding the actual scope of repositories, specifically their taxonomic and geographic coverage, is essential. We aimed to review and assess the availability and quality of barcodes for Phlebotominae species across the Americas, and to update the repository for species in Argentina. A database was constructed using sequences and metadata from scientific articles and DNA repositories. The species were classified based on the presence of barcodes for related species and their geographic representativeness. As of May-2024, the database includes 2,377 barcodes from 174 species and 19 genera. Only 10 genera have more than 30% of their species with barcodes, highlighting the need for careful attention when working with species with closely related species with no-barcodes. Ninety-one species have five or fewer barcodes, while 41 have barcodes from every country in their range. Of the 57 species with more than 10 barcodes, only 10 covers at least half of their geographic range. Out of 538 species in the Americas, barcodes are available for 174 (32%). This international effort has yielded notable outcomes, including the discovery of cryptic diversity. The accessibility of the technology and its potential for entomological surveillance is advancing more rapidly than the quality of the repositories. Given that both factors are crucial for reliable results and robust conclusions, continued efforts in enhancing barcode repositories are essential for maximizing the efficacy of these molecular tools.

Keywords: molecular taxonomy, cytochrome C Oxidase subunit I, New World.

*E*5 Ecology and Bionomics of Sandflies in the Panama Canal Corridor

Valderrama C. Anayansi[1]*, Henriquez C. Brigitte K.[1], Tuñon Anyi[1], González Marlin[1], Santos Hermes[1]

[1]*Department of Medical Entomology Research, Gorgas Memorial Institute for Health Studies, Panama*

***Corresponding author:** avalderrama@gorgas.gob.pa

Abstract

Sandfly-borne diseases played a crucial role in the Panama Canal construction history, and today, surveillance in the Canal Zone is essential to prevent emerging diseases. This study characterized the potential distribution of sandflies in the urban area of the Panama Canal Zone to identify threats. Sandflies were collected using mini light traps with photocells from June 13 to July 28, 2022, in forested areas, forest-urban edges, and residential zones of Cardenas de los Rios (Corozal), taking lunar phases into consideration. Sandflies were morphologically identified and the abundance of various species was quantified. Our results show that forested areas have the highest diversity and richness of sandfly species. The borderline area, although having high potential richness, shows a significant difference in species composition compared to the forest. The urban area has the lowest diversity and species richness, indicating a less suitable environment for sandflies. Bray-Curtis indices reveal variability in species composition across zones. The greater difference between the borderline and forest areas suggests a unique species composition in the borderline area due to its intermediate characteristics. The smaller difference between the forest and urban areas indicates some species overlap, but in different proportions. The historical relevance of sandflies in the region underscores the continuous importance of maintaining strict surveillance, as these diseases continue to pose a significant public health risk in the area. This study highlights the need for ongoing monitoring and control efforts to mitigate the impact of sandfly-borne diseases in the Panama Canal Zone.

Keywords: sandfly-borne diseases, Panama Canal zone, surveillance, species diversity, public health

List of authors

A
Abdo S. Abbas Mohammed - **O25, P42**
Adam Katja - **O35, O36**
Ajdary Soheila - **P21**
Akad Fouad - **O10**
Akihiro Kuroki - **O18**
Aklilu Esayas - **O23**
Albuquerque e Silva Rafaella - **O30, O31, P1**
Alcici de Souza Eduardo - **P16**
Alcover M. Magdalena - **O35, O36, O58**
Almeida Rocha Douglas - **O31**
Altug Unal - **O34, O39, O43**
Alvaro Alessandro - **O55**
Alves e Silva Thiago Luiz - **O44**
Alves Maria João - **O57, P2**
Amaro Fátima - **O57, P2**
Andersen John - **O44**
Andrade Andrey J. - **O45, O64, P11**
Andrade do Rosário Amanda - **O17, O32**
Andrade-Filho José D. - **O64**
Angelini Paola - **O20, P26**
Antoniou Maria - **O54**
Aoun Karim - **O7, O59, P3, P8**
Aparicio Lola - **P27**
Araújo Flores Gabriela - **O45**
Arnaud Frédérick - **O21**
Arroyo Santiago - **P41**
Arserim Suha Kenan - **O11, O33, O35, O36, O39, P4, P5**
Athanatos Manos - **O34, O43**
Atri Chiraz - **P23**
Ayhan Nazli - **O36, O56**
B
Babaie Jalal - **P21**
Babat Sefer Ö. - **P5**
Bacelar Paixão I. Matheus - **O30**
Balcioğlu I. Cüneyt - **O11, P4, P5, P40**
Bali Aymen - **P23**
Bandeira Maia Gledson - **O17**
Bandi Claudio - **O55**
Baneth Gad - **O10**
Bañuls Anne-Laure - **O35, O36, O50**
Barandika Jesús F. - **O35, O36, O58**
Barbosa Matheus E.P. - **P28, P30, P31**
Bardunek Valigurova Andrea - **O2**
Barillas-Mury Carolina - **O44**
Barletta Ana Beatriz F. - **O44**
Barón Jorge - **P27**
Barroso Edmilson Pereira - **P16**
Basmadjian Yester - **P6, P7**
Basseville François O35, O36
Bates Paul - **O3**
Batista Michel - **O48**
Battistoni Olivia - **O23**
Becvar Tomas - **O2, O3, O37, O49, O65**
Bedini Stefano - **O13**
Bekchiev Rostislav - **O62**
Bellali Hedia - **O59**
Ben Osman Raja - **O25**
Ben Said Mourad - **O25**
Benabid Meriem - **O7, O59, P3, P8**
Benallal Kamal Eddine - **O49, O60**
Ben-Avi Irina - **O10**
Benelli Giovanni - **O13**
Berberoglu Umut - **O39**
Bermejo San Frutos Ángela - **O63**
Bernardini Ilaria - **O35, O36, O37, O67, P26**
Berriatua Eduardo - **O34, O35, O36, O38, O43, O58**
Beust Quint Bruno - **P1**
Beverley Stephen M. - **O44**
Bezerra S. Marcos Antonio - **O13, O24**
Bianchi Riccardo - **O35, O37, O67, P26**
Bigoni Fabio - **O55**
Bisia Marina - **O35**
Bisogno Sara - **O46**
Blesić Suzana - **O34, O40, O42, O43**
Bongiorno Gioia - **O34, O35, O36, O37, O38, O43, O67, P26**
Boniface Namangala - **P10**
Boonserm Rungfar - **P39**
Bouattour Ali - **O29**
Bouhsira Emilie - **O35, O36**
Bouratbine Aïda - **O7, O59, P3, P8**
Bousbata Sabrina - **O28**
Boussaa Samia - **O28**
Boussoffara Thouraya - **O25, P42**
Boyer Sébastien - **O50**
Brey Paul - **O53**
Brezina Jiri - **O2**
Bruno Federica - **P37**
Buchy Philippe - **O53**
C
Cabrera Andrés - **P6, P7**
Caljon Guy - **O4**
Calvani C. Camila - **P30**
Calzolari Mattia - **O20, P9, P26**

Campolina Thais - **O19**
Carbonara Mariaelisa - **O24**
Cardoso de Oliveira Augusto - **O30**
Carra Elena - **O20**
Carrera Maya - **O20, P9**
Casaril Aline E. - **P28, P30, P31**
Castañeda-Casado Pablo - **O6**
Castelli Germano - **P37**
Cattaneo Giulia Maria - **O55**
Çavuş Ibrahim - **O4**
Cecilio Pedro - **O6, O8, O44**
Cena Cicero - **P30**
Çetin Hüseyin - **P5**
Cevidanes Miranda Aitor - **O35, O36, O58**
Charrel Remi - **O34, O36, O43, O56**
Chaskopoulou Alexandra - **P14, P19**
Chaves Luis F. - **O6**
Chebet Alphine - **O23**
Chelbi Ifhem - **O25, O38, P42**
Chemkhi Jomaa - **O29**
Chemkhi Safa - **O29**
Cherni Saifedine - **O25, P42**
Chizu Sanjoba - **O18**
Chonephetsarath Somsanith - **O53**
Christodoulou Vasiliki - **O54**
Colonna Roberta - **O20**
Costa-Ribeiro Magda C.V. - **P11**
Costa-Ribeiro Maria C. - **O45**
Courtenay Orin - **O14, O34, O38, O43**
Coutinho-Abreu Iliano V. - **O44**
Cova Bruno Oliveira - **O27**
Cristóvão Jose - **O36**
Cruz Maria do Socorro P. - **P12, P28, P31**
Cuadrado-Matías Raúl - **O35, O36, O58**
Cvetkovikj Aleksandar - **O56**
D
Da Silva F. Thiago - **P30**
Dachraoui Khalil - **O25**
Daisuke Kobayashi - **O18**
Dakien Osman M. - **O16, O23**
Dardé Marie-Laure - **O35, O36**
Davidovich-Cohen Maya - **O10, O34, O43**
De Andrade Andrey José - **P1**
De Araújo Nascimento Marlon G. - **P15**
De Ávila Márcia Moreira - **P15**
De Liberato Claudio - **P26**
Defilippo Francesco - **O20, P9**
Delacour Estrella Sarah - **O35, O36, O58**
Delaunay Pascal - **O35, O36**
Demir Samiye - **O11, P4, P33, P40**
Depaquit Jérôme - **O34, O35, O36, O43, O50, O51, O53, O60, O66, P24, P36, P39, P42**
Derghal Moufida - **O29**
Dey Ranadhir - **P42**
Di Muccio Trentina - **O36, O67, P26**
Di Pasquale Maria Liliana - **P22, P37**
Diaz Debora - **O10**
Díaz Mauricio - **P6, P7**
Díaz-Sáez Victoriano - **O35, O36, O58**
Dib Yassine - **O60**
Ding Huicong - **O51, P13**
Disotuar Maria M. - **O8, O44**
Doehl Johannes S.P. - **O44**
Doh Serena - **O23, O26**
Dottori Michele - **O20, P9**
Dutra-Rêgo Felipe - **O45, O47**
Dvořák Vít - **O28, O34, O35, O36, O37, O39, O43, O52, O56, O60, O61, O65, P14, P19, P25**
Dvorakova Barbora - **O2**
E
El Kacem Sofia - **O28**
Elbaz Shirly - **O10**
Elnaiem Dia-Eldin - **O16**
Enala T. Mwase - **P10**
England Marion - **O3**
Epis Sara - **O55**
Erisoz Kasap Ozge - **O34, O35, 36, O43**
Ertabaklar Hatice - **P40**
F
Facciponte Flavia - **P22**
Falchi Alessandra - **O35, O36**
Fathima PA - **O22**
Favennec Loïc - **O35, O36**
Felix Luiza dos Santos - **O8**
Fernandes Brilhante Andreia - **P15, P16**
Fernandes Wagner S. - **P28, P31**
Fernández Guillermo - **O35, O36, O58**
Ferraro Federica - **O34, O43**
Ferreira Bonfim Isabel - **O30**
Ferreira Maia Maria Joao - **O34, O43**
Ferreira Rangel Elizabeth - **P1**
Figueredo Fabiano - **O32**
Fiorentino Eleonora - **O36, O67, P26**
Fisa Roser - **O35, O36, O58**
Fleming Padraic - **O34, O43**
Flores Gabriela - **O1**
Foglia-Manzillo Valentina - **O34, O36, O43**
Fonseca Rocha Marilia - **O32**
Fontes Igor - **O45**
Fortuna Claudia - **O36, O67**

Foxi Cipriano - **P26**
Freitas Inês Campos - **O57, P2**
Froster Paola - **P6, P7**
Frynta Daniel - **O49**
Fuentes-Carias Marcela - **O9, P17**
G
Galati Eunice A.B. - **O32, O64, P16, P18, P28, P29, P31, P38, P43**
Gálvez Rosa - **O63, P27**
Galvis-Ovallos Fredy - **O17, O32, P12, P28, P29, P31**
Gannavaram Sreenivas - **P42**
Gantier Jean Charles - **P41**
Gargaté Maria João - **O57, P2**
Gay Frédérick - **P24**
Gebresilassie Araya - **O23**
Germinara G. Salvatore - **O13**
Gestich Carla - **O27**
Ghrab Jamila - **O7, O59, P3, P8**
Giardina Susanna - **P22**
Gligorić Nenad - **O38, O43**
Gligorijević Vladan - **O41**
Gomes A. Maurício - **P38, P43**
Gomes Carneiro Ana Carolina - **P16**
González Telma - **P6, P7**
Goto Hiro - **O14**
Gouzelou Stavroula (Evi) - **O54**
Gradoni Luigi - **P26**
Grisendi Annalisa - **O20, P9**
Guardado Diana - **O34, O43**
Guerfali Fatma Z. - **P23**
Guizani Ikram - **O29**
Gurgel-Gonçalves Rodrigo - **O14, O31**
H
Hailu Asrat - **O23**
Halada Petr - **O60, O61, P14, P19, P25**
Hamanno Shinjiro - **P42**
Hamdouni Mohamed - **P3**
Hamilton Gordon - **O34, O38, O43**
Hamilton James - **O14**
Hanusniakova Ida - **P14**
Harant Karel - **P34**
Harrat Zoubir - **O49**
Haruhiko Isawa - **O18**
Hatzakis Tally - **O34, O43**
Hempelmann Nils - **O34, O43**
Hendrickx Sarah - **O4**
Herman M. Chambaro - **P10**
Hernandez-Colina Arturo - **O38**
Heydarian Peyman - **P21**
Hlavackova Kristyna - **P19**
Hodzhev Yordan - **O62**
Hoskova Anna - **O37, P20**
Hoxha Ina - **O56, O65**
Huang Wei - **O6**
Huffcutt Patrick - **O23, O26**
Huguenin Antoine - **P24**
I
Iatta Roberta - **O24**
Indraccolo Francesca - **O24**
Infran O.M. Jucelei - **P12, P30, P31**
Ingonga Johnstone - **O23**
Iniguez Eva - **O6, O23, O26, O44**
Ivovic Vladimir - **O34, O35, O36, O43**
Izri Arezki - **O35, O36**
J
Jacobs-Lorena Marcelo - **O6**
Jahn Petr - **O3**
Jancarova Magdalena - **O21, O29, O37, P20**
Jelinkova Kristyna - **O2, O39**
Jellali Taieb - **P3**
Jemal Arij - **P3**
Jiménez Maribel - **O35, O36, O58**
Jucelei Infran O.M. - **P28**
K
Kamhawi Shaden - **O23, O26, O44**
Kanako Minagawa - **O18**
Karakuş Mehmet - **P4**
Karimian Fateh - **P21**
Kaunda Esther - **O23**
Kavan Daniel - **P19**
Kaviraj Swarnendu - **P42**
Kentaro Itokawa - **O18**
Khanahmadi Nasim - **P21**
Khositharattanakool Pathamet - **P35, P39**
Kiplagat Steve - **O23**
Kirstein Oscar David - **O10, O34, O35, O36, O43**
Kniha Edwin - **O34, O35, O36, O43, O56, O65**
Kohl Alain - **O21**
Kolarova Iva - **O2, O34, O39, O43, P34**
Koliou Maria - **O54**
Kowacich-Swaney Dannielle - **O15**
Kyoko Hayashida - **P10**
Kyoko Sawabe - **O18**
L
La Russa Francesco - **O35, P22, P37**
Labidi Imen - **O25, P42**
Lacerda O. Miller - **P30**
Lack Justin - **O44**

Lafri Ismail - **O60**
Lakeomany Khaithong - **O53**
Langourov Mario - **O62**
Laouini Dhafer - **P23**
Laurenti Márcia - **O1, O45**
Lavazza Antonio - **O20, P9**
Leite G.D.S. Jackellyne - **P12**
Lelli Davide - **O20, P9**
Lemrani Meryem - **O28, O61**
León Diego - **P6, P7**
Leštinová Tereza - **P34**
Liénard Emmanuel - **O35, O36**
Lim Kelvin - **O30, O51**
Limoncu M. Emin - **O11, P5**
Liu Kevin - **O30**
López de Felipe Marcos - **O63**
Louradour Isabelle - **O9, P17**
Louzir Hechmi - **P42**
Loyer Mathieu - **P24**
Luangamath Phonesavanh - **O53**
Lucientes Curdi Javier - **O35, O36, O58**
Lypaczewski Patrick - **P42**
M
M'Ghirbi Youmna - **O29**
Maachach Yassine - **O25**
Macanilla José - **P41**
Machado Paulo Roberto - **O27**
Maia Carla - **O35, O36, O38, O39, O43**
Maia Glédson B. - **O64**
Makwatta John Mark - **O23**
Maleki-Ravasan Naseh - **P21**
Manarolla Giovanni - **O20**
Mandysova Adela - **P25**
Mangiapelo Claudia - **O35, O36, O67, P26**
Manseur Hamza - **O60**
Marchand Lucien - **O28**
Marchini Fabricio - **O48**
Marialva dos Santos Eric F. - **O48**
Marsili Giulia - **O36, O67**
Martínez-Barciela Yasmina - **O35, O36, O58**
Martín-Martín Inés - **O35, O36, O58**
Martins Susana - **O57, P2**
Martín-Sánchez Joaquina - **O35, O36, O58**
Masiga Dan - **O23**
Mathieu Bruno - **O35, O36**
Matlashewski Greg - **P42**
Matoke Damaris - **O23**
Matta Vania L. - **O45**
Mefissel Mohammed - **O60**
Mekarnia Nalia - **O35, O36, O49**
Mendoza-Roldan J. Alfonso - **O24, O55**
Meneses Claudio - **O6, O8, O26, O44**
Mercier Aurélien - **O35, O36**
Mhaidi Idris - **O35, O61**
Michaelakis Antonios - **O35**
Mikov Ognyan - **O62**
Milagres Tarcísio - **O63, P27**
Milchram Markus - **O56**
Mkanenz Ghada - **P23**
Modry David - **O3**
Molina Ricardo - **O35, O36, O58**
Molteni Riccardo - **O55**
Monique Pinheiro Maia Silva - **P31**
Monte-Alegre Adriano - **O27**
Mora Ana I. - **O63**
Morales-Yuste Manuel - **O35, O36, O58**
Moreira de Ávila Márcia - **P16**
Moreira-Leite Flávia - **O5**
Morelli Letícia C. - **P11**
Mosca Andrea - **P26**
Muja Bajraktari Nesade - **O56**
Musa Ahmed M. - **O23**
N
Nacri Mehdi - **P24**
Nair Vinod - **O44**
Nakhasi Hira L. - **P42**
Nalçaci Muhammed - **P40**
Natal Sergio - **O40, O42**
Ng Germaine - **O51, P13**
Nogueira Letícia Amaral - **P15**
O
Obwaller Adelheid G. - **O56, O65**
Oerther Sandra - **O65**
Oguz Gizem - **O36**
Oliva Gaetano - **O36, O38, P3**
Oliveira Alessandra G. - **O32, O64, P12, P28, P29, P30, P31, P32, P38, P43**
Oliveira Fabiano - **O6, O8, O44**
Oliveira Janaína Estevo - **P15**
Oliveira Lidiane N. C. - **O64**
Oliveira Mosqueira Gabriela - **O31**
Oliveri Eugenia - **P22, P37**
Omondi Zeph Nelson - **P4, P5**
Orshan Laor - **O10**
Orsini Stefania - **O36**
Osman Omran F. - **O23**
Osório Hugo - **O57, P2**
Otranto Domenico - **O13, O24, O55**
Ouali Radouane - **O28**
Ovallos Fredy - **O1**

Owino Barrack - **O5**
Özbel Yusuf - **O11, O33, O34, O35, O36, O38, O39, O43, P4, P5, P33, P40**
Özbilgin Ahmet - **O11**
P
Pacáková Lenka - **O2, P34**
Panaiotov Stefan - **O62**
Parreira Ricardo - **O36**
Passero Felipe - **O1**
Pataradool Thanapat - **O52**
Pavlou Christoforos - **O54**
Paz Adolfo - **O35, O36, O58**
Paz Shlomit - **O34, O40, O42, O43**
Pekagirbas Metin - **O33, O35, O36, O39**
Pereira Lais da S. - **P42**
Pérez-Cutillas Pedro - **O35, O36, O58**
Perles Lívia - **O24**
Pessoa Felipe - **O48**
Phommavanh Nothasine - **O53**
Piazza Carlotta - **P37**
Piegas Sofía - **P7**
Pimenta Paulo - **O19**
Pinto Israel S. - **O64**
Pinto Mara Cristina - **O30**
Pistillo O. Marco - **O13**
Platzgummer Katharina - **O35, O36, O56, O65**
Poeppl Wolfgang - **O56, O65**
Polanska Nikola - **O21, O37, P20**
Polat Ceylan - **O36**
Polina Alejandro - **O35, O36, O58**
Pomares Christelle - **O35, O36**
Pongsanarm Tavun - **O53**
Ponnusamy Loganathan - **O15**
Posada-Lopez Laura - **O17, O32**
Prasetyo Didot B. - **O50**
Preativatanyou Kanok - **P35**
Promrangsee Chulaluk - **P35**
Prudhomme Jorian - **O35, O36, O66**
Pružinová Katerina - **O5**
Puggioli Arianna - **O20**
Pugh Aaron - **O26**
R
Rahola Nil - **O50**
Ramírez Sebastián - **P41**
Ramos de Almeida Victor - **O48**
Ramos Leandro José - **P15**
Randrianambinintsoa J. Fano - **O35, O36, O50, O51, O53, P24, P36, P41**
Ratinier Maxime - **O21**
Reale Stefano - **P22, P37**
Rêgo Felipe D. - **O64**
Rehbergerova Marketa - **O21**
Reis Tânia - **O57, P2**
Rhim Adel - **O29**
Ribeiro José M. C. - **O44**
Rios Velasquez Claudia - **O48**
Risueño José - **O35, O36, O38, O58**
Robert-Gangneux Florence - **O34, O35, O36, O43, O66**
Roca-Geronès Xavier - **O35, O36, O58**
Rocha G. Elisene - **P38, P45**
Rocha Marília Fonseca - **O17**
Rodrigues Janneth - **O6**
Rodrigues L. Bruno - **O64, P18, P29**
Rogerio Luana A. - **O6**
Rugna Gianluca - **O20**
Ruiz-Fons Francisco - **O35, O36, 058**
Rungfar Boonserm - **P25**
Ruzickova Katerina - **02**
S
Sádlová Jovana - **O3, 05, O28, O34, O37, O43, O49, O61, O65, P20**
Saenchaiban Nattiya - **P39**
Sagi Orly - **O10**
Saini Prasanta - **O22**
Şakru Nermin - **P40**
Salmen Raffoul Gabriel Elias - **P1**
Salvato Sara - **O20**
Salvemini Marco - **O46**
Samaniego Luis - **O34, O43**
San Martin Daniel - **O34, O42, O43**
Sánchez Andrade Rita - **O35, O36, O58**
Sanchez-Ruiz Lorenzo - **O55**
Sandoval Carmen - **O45**
Sanjoba Chizu - **O33, P4, P10**
Santos R. O. Luiz Gustavo - **P32**
Santos Thiago Vasconcelos - **O64**
Sanum Pinpinat - **P39**
Saraiva Elanderson F. - **P28, P31**
Saranholi Bruno - **O27**
Sassù Fabiana - **O47**
Satoskar Abhay - **O23, P42**
Satta Giuseppe - **P26**
Sayadi Aida - **O25**
Scalone Aldo - **O36**
Schal Coby - **O15**
Schriefer Albert - **O27**
Scibetta Silvia - **P22, P37**
Scremin Mara - **O20, P9**
Secundino Nagila - **O19**

Serafim Tiago D. - **O6, O8, O26, O44**
Sevil Naser - **P33**
Sevila Julie - **O35, O36**
Sevim Kivanc O36
Shah Harish Kumar O22
Shaw Jeffrey - **O12, O45**
Sherifi Kurtesh - **O56**
Shinji Kasai - **O18**
Short Mara - **O44**
Sierra Romero G. Adolfo - **P1**
Silva F. C. Antonio - **P38, P43**
Silva Leonardo E.H. - **O64**
Silva Manuel - **O57, P2**
Silva Monique Pinheiro Maia - **P28**
Simov Nikolay - **O62**
Singh Kamaleshwar P. - **P42**
Singh Sanjay - **P42**
Siriyasatien Padet - **O3, O52, P25, P35, P39**
Sng Wendy - **O51**
Soares Patrícia - **O57**
Soares Rodrigo P. P. - **O45, O47**
Socorro Cruz Maria - **O32**
Soheili Majid - **O40, O42**
Somwang Puckavadee - **P35, P39**
Souguir-Omrani Hajer - **O29**
Souissi Olfa - **O7, P8**
Sousa Félix de Lima Mayra - **O31**
Souza Lencina Matheus Vitor - **P15**
Souza N. Rebeccah - **P32**
Späth Gerald F. - **O9, P17**
Spelta Guilherme - **O48**
Stefanovska Jovana - **O56**
Stejskalova Marketa - **O21, O29, O37, P20**
Studentsky Liora - **O10**
Sunantaraporn Sakone - **O52, P25, P39**
Sunter D. Jack - **O5**
T
Taddei Roberta - **O20**
Takashi Marcos Obara - **O31**
Tan Cheong Huat - **O51, P13**
Tan Denise - **O51, P13**
Tang Kristina - **O6, O8**
Tantely Luciano - **P36**
Tatsuki Sugi - **P10**
Tebai Abir - **O29**
Telleria L. Erich - **O46, O47**
Tempone Antonio J. - **O48**
Thiesson Adrien - **O21**
Tomokane Thaise - **O1**
Torno Majhalia - **O51, P13**
Torres-Sobrevilla Carla - **P41**
Töz Seray - **O11, O33, O35, O36, O38, O39, P4, P5, P33, P40**
Trájer Attila J. - **O56, O65**
Traub-Cseko Yara M. - **O48**
Trimèche Malek - **O25, P42**
Tsafarova Borislava - **O62**
Tuluso Mercy - **O23**
Tumminia Silvia - **P22, P37**
Tuñon Sandra O43
U
Ulloa Catalina - **P41**
Unchanam Isaraporn - **P39**
Urošević Vladimir - **O38**
V
Valenzuela Jesus G. - **O6, O8, O26, O44**
Van Langevelde Frank - **O34, O43**
Varikuti Sanjay - **P42**
Varotto-Boccazzi Ilaria - **O29, O55**
Vega-Rodriguez Joel - **O44**
Verger Lorenzo - **P6, P7**
Vianna Elisa - **O14**
Vieira Stahl Cecilia - **O46**
Vilares Anabela - **O57, P2**
Villari Sara M. - **P22**
Vincent Robert - **P36**
Vitale Fabrizio - **P22, P37**
Vojtkova Barbora - **O2, O3, O28, O37, O49, O61, O65, P20**
Volf Petr - **O2, O3, O5, O13, O21, O24, O28, O29, O34, O37, O39, O43, O46, O47, O49, O52, O60, O65, P14, P19, P20, P25, P34**
Volfová Věra - **O39**
Vomáčková-Kykalová B. - **O47**
Vongphayloth Khamsing - **O50, O51, 053**
Votypka Jan - **O3**
Vungkyly Veaky - **O53**
W
Waitz Yoni - **O40, O42**
Walochnik Julia - **O56, O65**
Wang Danyang - **O40, O42**
Wasserberg Gideon - **O15**
Werneck Guilherme L. - **O17, O32, P1, P12, P28, P31**
Weslati Marwa - **O7**
Willat Gabriela - **P6, P7**
Willen Laura - **O6**
Wilson Timothy - **O44**
Wongkhut Wilai - **P39**
Wright David - **O34, O43**

X
Xhekaj Betim - **O56**
Y
Yanase Ryuji - **O5**
Yasuyuki Goto - **P10**
Yetismis Kardelen - **O33, O35, O36, O39, P5**
Yilmaz Ayda - **O36**
Younis Brima M. - **O23**
Yuan Jin-Min - **O52**
Z
Zapata Sonia - **P41**
Zeni Valeria - **O13**
Zhang Wen-Wei - **P42**
Zhang Yue - **O6**
Zhioua Elyes - **O25, P42**
Zhou Guofa - **O23**
Zribi Lilia - **P3**
